# Genomic and Transcriptomic Determinants of Therapy Resistance and Immune Landscape Evolution during Anti-EGFR Treatment in Colorectal Cancer

**DOI:** 10.1016/j.ccell.2019.05.013

**Published:** 2019-07-08

**Authors:** Andrew Woolston, Khurum Khan, Georgia Spain, Louise J. Barber, Beatrice Griffiths, Reyes Gonzalez-Exposito, Lisa Hornsteiner, Marco Punta, Yatish Patil, Alice Newey, Sonia Mansukhani, Matthew N. Davies, Andrew Furness, Francesco Sclafani, Clare Peckitt, Mirta Jiménez, Kyriakos Kouvelakis, Romana Ranftl, Ruwaida Begum, Isma Rana, Janet Thomas, Annette Bryant, Sergio Quezada, Andrew Wotherspoon, Nasir Khan, Nikolaos Fotiadis, Teresa Marafioti, Thomas Powles, Stefano Lise, Fernando Calvo, Sebastian Guettler, Katharina von Loga, Sheela Rao, David Watkins, Naureen Starling, Ian Chau, Anguraj Sadanandam, David Cunningham, Marco Gerlinger

**Affiliations:** 1 Translational Oncogenomics Lab, The Institute of Cancer Research, 237 Fulham Road, London SW3 6JB, UK; 2 GI Cancer Unit, The Royal Marsden Hospital, London SW3 6JJ, UK; 3 Centre for Evolution and Cancer Bioinformatics Team, The Institute of Cancer Research, London SW3 6JB, UK; 4 Systems and Precision Cancer Medicine Lab, The Institute of Cancer Research, London SW3 6JB, UK; 5 Cancer Institute, University College London, London WC1E 6AG, UK; 6 Tumour Microenvironment Lab, The Institute of Cancer Research, London SW3 6JB, UK; 7 Department of Radiology, The Royal Marsden Hospital, London SW3 6JJ, UK; 8 Departments of Pathology and Histopathology, University College Hospital, London NW1 2PG, UK; 9 Barts Cancer Institute, Queen Mary University, London EC1M 6BQ, UK; 10 Division of Structural Biology, The Institute of Cancer Research, London SW3 6JB, UK

**Keywords:** cancer evolution, EGFR, drug resistance mechanisms, molecular subtype, colorectal cancer, cetuximab, predictive biomarker, immunotherapy, cancer genomics, cancer-associated fibroblasts

## Abstract

Despite biomarker stratification, the anti-EGFR antibody cetuximab is only effective against a subgroup of colorectal cancers (CRCs). This genomic and transcriptomic analysis of the cetuximab resistance landscape in 35 RAS wild-type CRCs identified associations of *NF1* and non-canonical RAS/RAF aberrations with primary resistance and validated transcriptomic CRC subtypes as non-genetic predictors of benefit. Sixty-four percent of biopsies with acquired resistance harbored no genetic resistance drivers. Most of these had switched from a cetuximab-sensitive transcriptomic subtype at baseline to a fibroblast- and growth factor-rich subtype at progression. Fibroblast-supernatant conferred cetuximab resistance *in vitro*, confirming a major role for non-genetic resistance through stromal remodeling. Cetuximab treatment increased cytotoxic immune infiltrates and PD-L1 and LAG3 immune checkpoint expression, potentially providing opportunities to treat cetuximab-resistant CRCs with immunotherapy.

## Highlights

• *NF1* and non-canonical *KRAS* and *BRAF* aberrations associate with cetuximab resistance
• Genetic resistance drivers are absent in most biopsies that acquired resistance
• Stromal remodeling is an alternative non-genetic mechanism of cetuximab resistance
• Cetuximab-mediated immune modulation may sensitize CRCs to immunotherapy

## Teaser

Woolston et al. show that in metastatic colorectal cancer cetuximab resistance can be conferred by genetic mechanisms, such as *NF1* loss or RAS/RAF alterations, or by transcriptomic changes that induce a stroma-rich phenotype. They also provide a rationale for combining cetuximab with immunotherapy.

## Significance

**Only 43% of patients had prolonged benefit from cetuximab in this trial despite treatment stratification by RAS mutations. The identified associations of *NF1*, non-canonical *KRAS* and *BRAF* aberrations with primary resistance, and of CMS2/TA transcriptomic subtypes with prolonged benefit may enable more effective treatment allocation and avoid toxicities from ineffective therapy. Genetic resistance drivers were not identified in the majority of metastases that had acquired resistance. Most of these had switches from the cetuximab-sensitive CMS2/TA subtype to a fibroblast- and growth factor-rich subtype. This challenges the paradigm that genetic drivers predominate at acquired resistance and suggests therapeutic approaches by targeting fibroblasts. Increased T cell infiltration and immune checkpoint upregulation following cetuximab responses warrant trials of checkpoint inhibitors.**

## Introduction

Anti-epidermal growth factor receptor (EGFR) antibodies (anti-EGFR-Ab) are effective in a subgroup of patients (pts) with metastatic colorectal cancer (CRC). Activating *KRAS* or *NRAS* mutations in codons 12, 13, 59, 61, 117, and 146 have been associated with primary resistance in randomized trials and anti-EGFR-Ab treatment should only be administered for tumors that are wild type (WT) at these loci (Allegra et al., 2016, Amado et al., 2008, Bokemeyer et al., 2012, Douillard et al., 2013, Tejpar et al., 2016). Despite this stratification, many pts do not benefit, indicating additional resistance mechanisms. *BRAF* V600E (Loupakis et al., 2009), *MAP2K1* (encodes for MEK1) (Bertotti et al., 2015), or *PIK3CA* (Sartore-Bianchi et al., 2009) mutations, amplifications (amp) of *KRAS* (Valtorta et al., 2013), and of the receptor tyrosine kinase (RTK) genes *ERBB2*, *MET*, and *FGFR1* (Bertotti et al., 2015), have been suggested as further drivers of primary resistance but are not recommended for routine use due to insufficient validation in clinical trials. Moreover, a recent transcriptomic classification of CRCs into distinct subtypes found an association of the transit amplifying (TA) subtype with cetuximab (CET) sensitivity (Sadanandam et al., 2013), suggesting that non-genetic molecular characteristics also influence anti-EGFR-Ab sensitivity.

Anti-EGFR-Ab acquired resistance (AR) almost invariably occurs in pts who initially benefit, and this has predominantly been studied retrospectively in circulating tumor DNA (ctDNA) (Bettegowda et al., 2014, Diaz et al., 2012, Misale et al., 2012). *KRAS* and *NRAS* (herein RAS) mutations, as well as EGFR exodomain mutations that alter the binding epitope for the anti-EGFR-Ab CET have been found in ctDNA from a large proportion of pts with AR. Amp of *MET* or *KRAS* evolved in some pts (Bardelli et al., 2013, Mohan et al., 2014, Siravegna et al., 2015). The high prevalence of RAS mutations supports the notion that mechanisms of primary and AR are often similar. A small number of studies assessed anti-EGFR-Ab AR in tumor biopsies (Misale et al., 2012, Van Emburgh et al., 2016). These also identified RAS and *EGFR* mutations, but their retrospective nature and the analysis of only a small number of candidate genes may have biased the results. Ligands for the RTKs EGFR and MET (Hobor et al., 2014, Liska et al., 2011) confer anti-EGFR-Ab resistance *in vitro* but their clinical relevance remains unknown. Detailed insights into resistance mechanisms may enable more precise therapy allocation to pts who are likely to respond and open therapeutic opportunities for CET-resistant CRCs.

## Results

**Figure 1:**
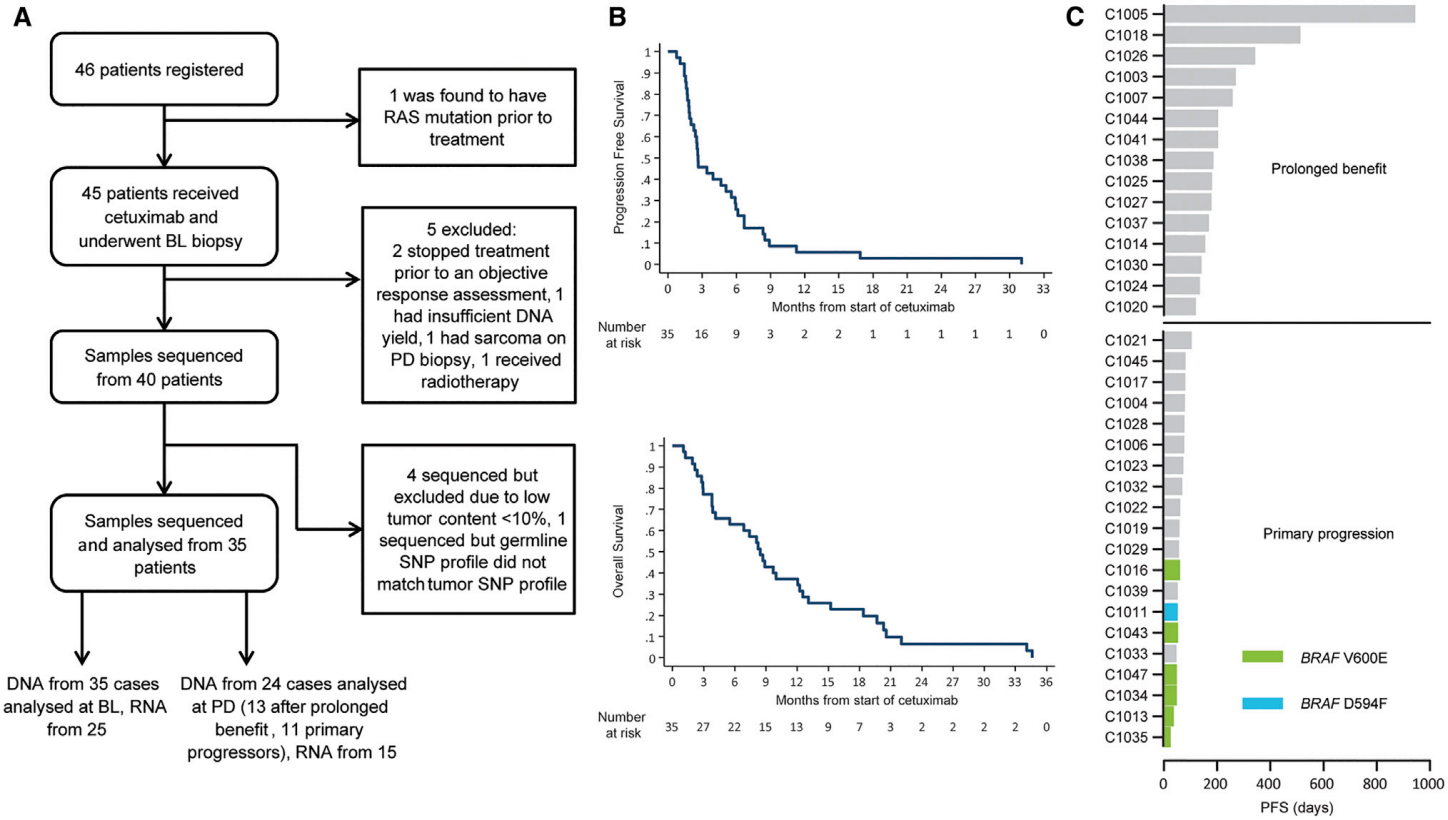
CONSORT Diagram and Survival Data (A) CONSORT diagram of 46 patients (pts) included and biopsy samples analyzed. BL, baseline; PD, progressive disease. (B) Kaplan-Meier survival analysis of 35 pts whose samples were subjected to molecular analysis. (C) Swimmer plot of progression-free survival (PFS) data and separation into pts with prolonged benefit and with primary progression. See also Tables S1 and S2.

**Figure 2:**
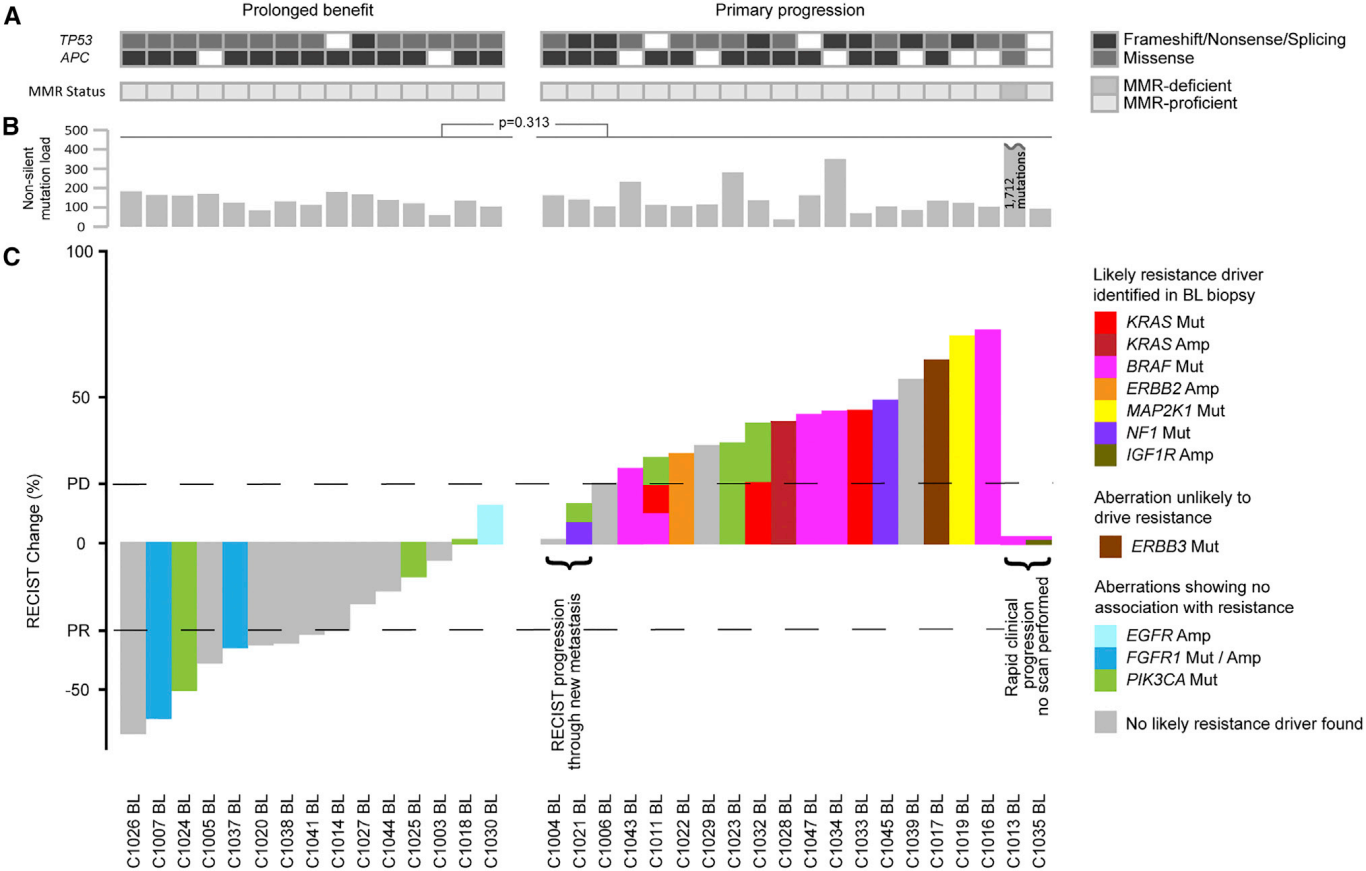
Molecular Profiles of 35 BL Biopsies Categorized into Cases with Prolonged Cetuximab Benefit and Primary Progressors (A) *TP53* and *APC* mutations and microsatellite instability status. (B) Non-silent mutation load. The p value was calculated using the Student's t test. (C) Waterfall plot of best radiological response and genetic aberrations of RAS/RAF pathway members or regulators and *PIK3CA*. Amp, amplification; Mut, mutation; PR, partial response; PD, progressive disease as per RECIST criteria. See also Data S1 and S2.

Forty out of 45 pts treated with single-agent CET could be assessed for treatment response and had sufficient biopsy material available for molecular analyses. Sequencing of baseline (BL) biopsies failed in 5 cases, leaving 35 for study (Figure 1A; Tables S1 and S2). The median progression-free survival (PFS) and overall survival of this cohort were 2.6 and 8.5 months, respectively (Figure 1B). Twenty pts showed primary progression at or before the first per protocol computed tomography scan (scheduled at week 12). The remaining 15 were classified as pts with prolonged clinical benefit (Figure 1C). As expected for CRC, *TP53* and *APC* mutations were common, and one tumor showed mismatch repair deficiency (Figure 2A). The mutation burden did not significantly differ between tumors with prolonged benefit (median = 134) and primary progressors (median = 120, Figure 2B). Progressive disease (PD) biopsies were taken after radiological progression (median 14 days after CET cessation) from 25/35 cases, and 24 were successfully exome sequenced. Sufficient RNA for RNA sequencing (RNA-seq) was obtained from 25 BL and 15 matched PD biopsies.

### Genetic Drivers of Primary Resistance

**Figure 3:**
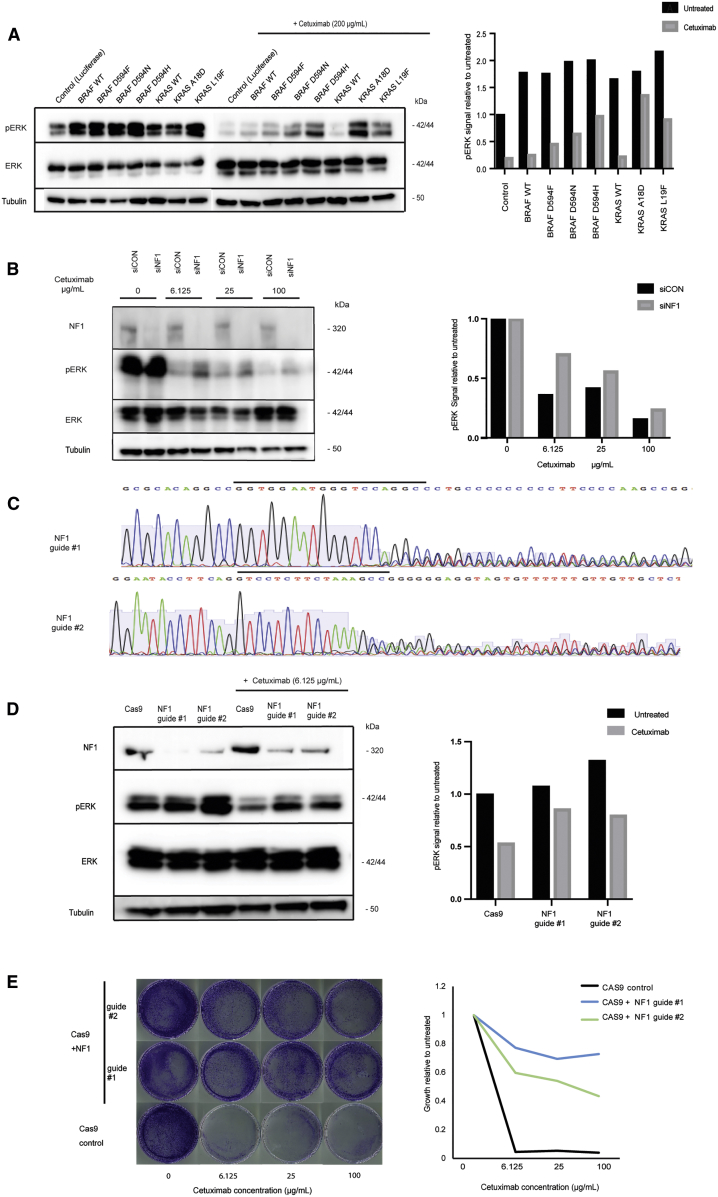
Functional Impact of RAS/RAF Mutations and *NF1* Inactivation on Cetuximab Sensitivity (A) Western blot of BRAF and KRAS mutants in DiFi cells. Quantification of pERK signal relative to total ERK as a loading control, and normalized to luciferase control. (B) Western blot following NF1 (siNF1) or control (siCON) small interfering RNA in LIM1215 cells. Quantification of pERK signal relative to total ERK, and normalized to untreated control. (C) Sanger sequencing of LIM1215 cells transduced with two CRISPR guide RNAs against *NF1*. Guide sequences are highlighted by a black bar. (D) Western blot of CRISPR-inactivated *NF1* and Cas9 control cells with/without 24 h cetuximab treatment. Quantification of pERK signal relative to total ERK and normalized to untreated Cas9 control. (E) Growth of CRISPR-inactivated *NF1* and Cas9 control cells by crystal violet staining (left) and quantification (right).

We first aimed to identify resistance drivers in BL biopsies from 20 primary progressors (Figure 2C). Oncogenic *BRAF* V600E mutations were present in six pts, one in combination with *IGF1R* amp (C1035BL, Data S1). No radiological response occurred in any of these and PFS was short, supporting previous data that *BRAF* V600E confers resistance to CET (Pietrantonio et al., 2015). C1011BL harbored a non-canonical *BRAF* D594F mutation, disrupting the DFG motif of the kinase site. This is predicted to lead to a kinase-impaired BRAF variant (Moretti et al., 2009), which has been shown to paradoxically hyperactivate downstream ERK phosphorylation (pERK) when combined with oncogenic RAS alterations (Heidorn et al., 2010). C1011BL indeed harbored a concomitant *KRAS* L19F mutation, which has an attenuated phenotype compared with canonical *KRAS* mutations (Smith et al., 2010). Stable expression of BRAF D594F or KRAS L19F in the CET-sensitive DiFi CRC cell line confirmed that each was individually able to maintain a moderate level of pERK despite CET treatment (Figure 3A), supporting a mechanistic role in resistance. It is conceivable that together both mutations further increase pERK signaling leading to fitness advantages that may explain co-occurrence in C1011BL. Another *KRAS* mutation (A18D), which confers an attenuated phenotype *in vitro* (Scholl et al., 2009), was encoded on all seven copies of the polysomic chr12p in C1033BL (Data S2), likely explaining resistance in this case. Introduction of KRAS A18D into DiFi cells promoted strong pERK during CET exposure (Figure 3A), providing biochemical support for its role in resistance. A *KRAS* G12D mutation was identified in C1032BL, which had been found to be *KRAS* WT before study entry, indicating either a false-negative result of the clinical assay or intratumor heterogeneity. A *KRAS* amp was present in C1028BL and an *ERBB2* amp in C1022BL (Data S1). C1019BL harbored a canonical activating *MAP2K1* mutation (K57N) and a concomitant *MAP2K1* mutation (S228A), which did not influence kinase activity in a previous study (Pages et al., 1994). Two tumors carried disrupting mutations in *NF1* (C1021BL, frameshift; C1045BL, nonsense). Both showed loss of heterozygosity of the *NF1* locus (Data S2), constituting biallelic inactivation of this tumor suppressor gene. *NF1* encodes for a negative regulator of KRAS and inactivation leads to EGFR inhibitor resistance in lung cancer (de Bruin et al., 2014). Small interfering RNA and CRISPR/Cas9 inactivation of *NF1* in CET-sensitive LIM1215 cells rescued a moderate level of pERK during CET treatment (Figures 3B–3D). CRISPR/Cas9 engineered *NF1* deficiency furthermore maintained cancer cell growth despite CET treatment (Figure 3E). These data suggest *NF1* inactivation as a driver of primary CET resistance in CRC. *ERBB3* was mutated (P590L) in C1017BL but this codon change had no impact on *in vitro* growth in a previous study (Liang et al., 2012), questioning whether it confers CET resistance.

In contrast to previous studies (Bertotti et al., 2015, Sartore-Bianchi et al., 2009), neither *PIK3CA* nor *FGFR1* aberrations clearly associated with resistance (Figure 2C): 4/20 pts (20%) with primary progression harbored activating *PIK3CA* mutations (2xE545K, G364R, and H1047R concomitant with *PIK3CA* amp; Data S2), but also 3/15 pts (20%) with prolonged benefit (2xV344G, H1047R). A tumor with a high level *FGFR1* amp (C1037BL) and one with an *FGFR1* R209H mutation (C1007BL), previously reported in Kallmann syndrome (Laitinen et al., 2011), had partial responses and prolonged benefit. An *EGFR* amp was found in one tumor (C1030BL) and this associated with prolonged benefit as described previously (Bertotti et al., 2015).

Together, oncogenic aberrations of RAS/RAF pathway genes or RTKs that could explain resistance were identified in 14/20 pts (70%) with primary progression.

### Validation of Transcriptomic Subtypes as Non-genetic Predictors of CET Benefit

**Figure 4:**
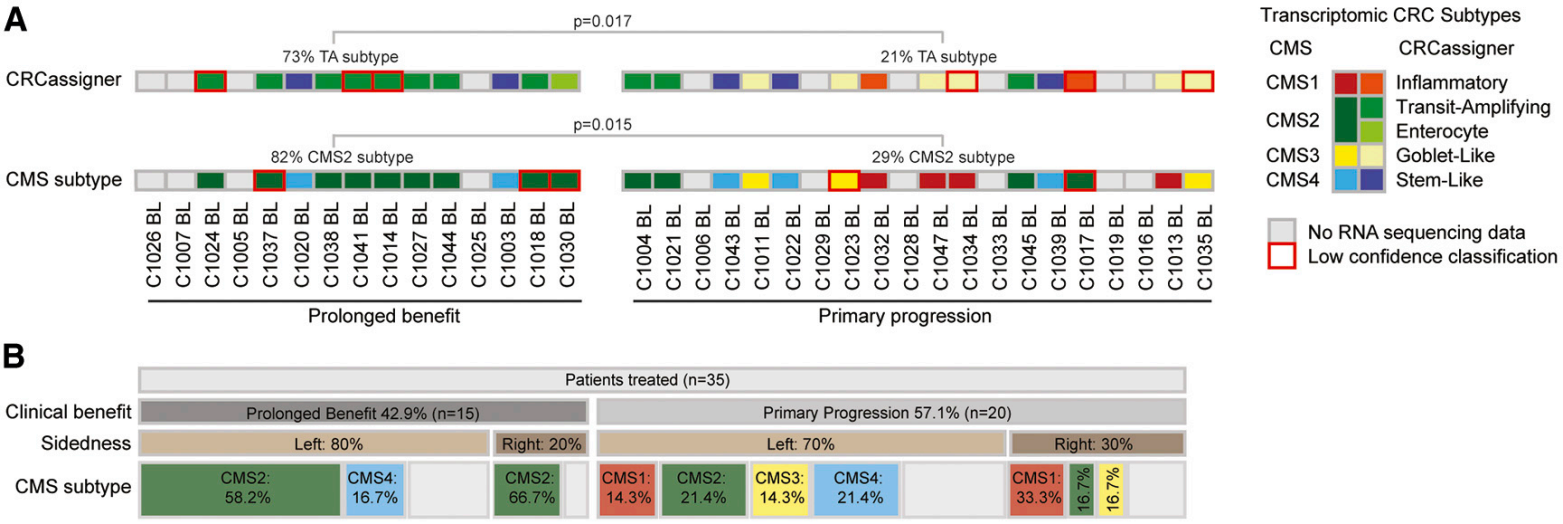
Transcriptomic Subtypes of BL Biopsies Categorized into Cases with Prolonged Cetuximab Benefit and Primary Progressors (A) Transcriptomic subtype assignment. The figure legend for the transcriptomic subtypes is arranged to show the most similar CMS and CRCassigner subtypes next to each other. Significance was assessed by the Fisher's exact test. (B) Association of clinical benefit with tumor sidedness and CMS subtype. See also Figure S1.

BL biopsies for which RNA-seq could be performed (n = 25) were next assigned to transcriptomic CRC subtypes using the CRCassigner (Sadanandam et al., 2013) and the consensus molecular subtype (CMS) classifications (Guinney et al., 2015) (Figure S1A). There are strong similarities between subtypes of both classifications, and 21/25 cases (84%) were assigned to matching subtypes, confirming robust performance (Figure 4A). The TA subtype has previously been associated with CET sensitivity (Sadanandam et al., 2013) and was 3.4-fold enriched (p = 0.017) among cases with prolonged benefit. The TA subtype is most similar to the CMS2 subtype, and was 2.9-fold enriched (p = 0.015) among pts with prolonged CET benefit. This validates the TA/CMS2 subtypes as non-genetic predictors of single-agent CET benefit. As described (Khambata-Ford et al., 2007), tumors with CET benefit also expressed higher levels of the EGFR ligands AREG and EREG (Figure S1B).

Pts with right-sided colon cancers do not benefit from first-line combination therapy with CET and chemotherapy, even if they are RAS/RAF WT, but whether right-sided tumors benefit from CET beyond first-line remains a matter of debate (Weinberg, 2018). Three pts with right-sided tumors showed prolonged benefit from single-agent CET in this trial (Figure 4B). CMS subtype information was available for two of these and both displayed the CET-sensitive CMS2. CMS subtype may be more relevant than sidedness for response prediction to single-agent CET beyond the first-line setting.

### Genetic Drivers of AR

**Figure 5:**
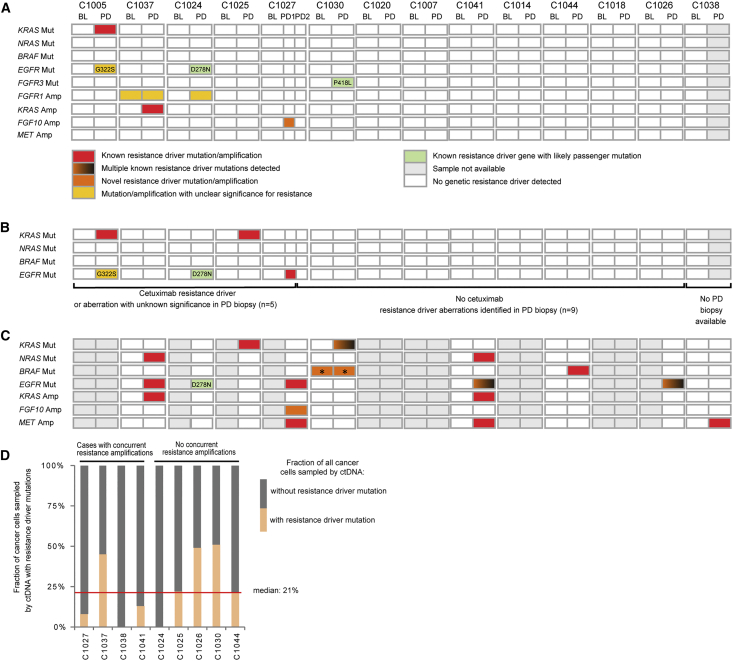
Genetic Alterations in RAS/RAF Pathway Members and Regulators at AR in 14 Cases (A) Mutations/amps identified by exome sequencing (158×) of biopsies. (B) Mutations identified by deep amplicon sequencing (2,179×) of *KRAS*, *NRAS*, *BRAF*, and *EGFR* in biopsies; color key as in (Figure 5A). (C) Mutations/amps identified by circulating tumor DNA (ctDNA) sequencing (1,048×); color key as in (A); ^∗^indicates present at BL but with substantial increase in mutation abundance at PD. (D) Fraction of cancer cells sampled by ctDNA that harbored a resistance driver mutation at PD. BL, baseline; PD, progressive disease. See also Figures S2, S3, Data S3, and Tables S4 and S5.

PD biopsies from 14 metastases (mets) that radiologically progressed after prolonged clinical benefit were successfully exome sequenced (Figure 5A), including biopsies from two different progressing mets in C1027. We first investigated genes with a known role in CET resistance. Only one *KRAS* mutation was acquired among these PD biopsies (C1005PD, G12C). This clonally dominant mutation (Data S2) was accompanied by an *EGFR* mutation (G322S), which has not previously been described and whose relevance is uncertain in the context of a well-characterized CET resistance mutation in *KRAS*. One biopsy acquired a *KRAS* amp (C1037PD). C1024PD acquired a clonally dominant *EGFR* mutation that has not previously been described (D278N), locating to the EGFR extracellular domain II (Schmiedel et al., 2008) but not affecting CET binding epitopes. Expression of EGFR D278N in the LIM1215 cells did not confer CET resistance and introduction into 3T3 fibroblasts showed no evidence of constitutive EGFR phosphorylation (Figures S2A and S2B), suggesting that this is a passenger mutation. No other *RAS*, *EGFR*, *BRAF*, or *ERK* mutations or amps were detected in PD biopsies.

Two further RTK genes acquired mutations at PD: *FGFR3* in C1030PD (P418L) (Figure 5A) and *ALK* in C1024PD (D626H) (Table S2). Neither is located to the well-defined mutational hotspots in these genes or has been reported in the COSMIC cancer mutation database (Forbes et al., 2010), indicating that these may be passenger mutations. Computational prediction showed a high driver score for *FGFR3* P418L (Tamborero et al., 2018), but functional analysis showed no rescue of pERK during CET treatment (Figure S2C). C1024PD acquired an *FGFR1* amp (Data S1). However, the presence of an *FGFR1* amp in C1037BL, who subsequently responded to CET (Figure 2C), questions whether this is sufficient to establish resistance. C1027PD1 acquired a narrow amp (1.58 Mbp, 60 DNA copies) encompassing *FGF10* (Figure S2D). *FGF10* encodes a ligand of the FGFR2 RTK, which is expressed in most CRCs (Otte et al., 2000). Recombinant FGF10 rescued growth and pERK in CRC cell lines treated with CET, supporting the notion that the acquired *FGF10* amp drives resistance in C1027PD1 (Figure S2E). FGF10-induced resistance could be reversed by treatment with a pan-FGFR inhibitor (FGFRi) (Figures S2F and S2G). Different contributions of FGFR1 and FGFR2 to CET resistance may result from differences in downstream signaling events (Pearson et al., 2016).

We also investigated genes that recurrently acquired mutations in PD biopsies to identify potential drivers of AR beyond the RAS/RAF pathway. Five genes had each acquired mutations in two PD biopsies (Table 1). All genes were large and we found no evidence of biallelic inactivation, which would be expected for tumor suppressor genes, or for recurrence of mutations in specific functional domains or amino acid positions, which would indicate gain-of-function mutations either in our samples or in the COSMIC mutation database. Thus, none of these genes were considered likely to confer CET resistance (Table S3).

**Table 1:** Recurrently Mutated Genes in PD Biopsies

Gene	Protein Size (Amino Acids)	Mutations Present in PD Samples but Absent in Matched BL Samples
*FAM120C*	1,096	C1025PD (G981S), C1030PD (R395X)
*DSPP*	1,301	C1014PD (S803fs), C1025PD (G260R)
*PTPN14*	1,187	C1020PD (L510P), C1030PD (D596H)
*NBEA*	2,946	C1007(M2285I), C1026PD (S189R)
*FAT3*	4,589	C1020PD (K4152T), C1025PD (P2099S)

See also Table S3.

### Genetic Drivers of AR Are Undetectable in Most PD Biopsies despite Ultra-deep Sequencing

CET AR is often polyclonal (Bettegowda et al., 2014), and sequencing of PD biopsies with a mean depth of 158× may have failed to detect resistance mutations in small subclones. We hence re-sequenced known CET driver hotspots in *KRAS*, *NRAS*, *BRAF*, *MEK1*, and *EGFR* by deep (2,179×) amplicon sequencing in order to call mutations with variant allele frequencies (VAFs) as low as 0.5% (Figure 5B; Table S4). This revealed a *KRAS* Q61H mutation in C1025PD (VAF, 4.9%) and an *EGFR* exodomain S492R mutation in C1027PD1 (VAF, 2.1%). Both are known to confer CET AR and were subclonal in these PD samples (Data S2).

Taken together, we identified known and not previously described CET resistance drivers in four PD biopsies. One case acquired an *FGFR3* mutation with unlikely relevance and one an *FGFR1* amp with unclear relevance for resistance. Importantly, no drivers of AR were found in 9/14 (64%) biopsied mets despite each radiologically progressing (Figure S3).

### Genetic Drivers of AR in ctDNA

The low prevalence of CET resistance drivers in PD biopsies was striking as it contrasts with results of ctDNA analyses of this trial and others that reported the evolution of RAS and *EGFR* aberrations in the majority of pts at the time of CET AR (Bettegowda et al., 2014, Khan et al., 2018). To assess the prevalence and clonality of resistance drivers in ctDNA, we applied a ctDNA sequencing (ctDNA-seq) assay targeting CET resistance and CRC driver genes (Table S5), which simultaneously infers genome-wide copy-number profiles (Mansukhani et al., 2018). This enabled us to correct VAFs for the influence of copy-number states and to then quantify the proportion of the cancer cells that harbored resistance drivers by comparison against *TP53* mutations, which are usually truncal in CRC (Brannon et al., 2014). Available ctDNA from nine pts that progressed after prolonged CET benefit (five BL/PD pairs, four PD only) was deep sequenced (1,048×). Known CET resistance mutations in *RAS*, *BRAF*, or *EGFR* were identified in 7/9 cases (78%) at PD (Figure 5C; Table S5). A kinase-impairing *BRAF* mutation (D594N) was detected in 6.8% of the cancer cell fraction in ctDNA at BL and this increased to 37.4% at PD in C1030 (Table S5). BRAF D594N rescued pERK in DiFi cells during CET treatment (Figure 3A). Together with the identification of a kinase-impairing *BRAF* mutation in a primary resistant tumor (C1011BL), this substantiates a role of *BRAF* D594 mutations in CET resistance. DNA copy-number profiles generated from ctDNA at PD furthermore identified amps of *MET* and *KRAS* in three and two cases, respectively (Figure 5C; Data S3). The *FGF10* amp found in the C1027PD1 biopsy was also identified at PD. Overall, ctDNA-seq revealed genetic drivers of AR in 8/9 pts (89%) and frequent polyclonal resistance, similar to published ctDNA results (Bettegowda et al., 2014). We next used *TP53* mutations, detected in all ctDNA samples, to estimate the fraction of the cancer cell population represented in the ctDNA that harbored AR mutations at PD (Table S5). All detected AR driver mutations taken together in each tumor were confined to a median 21% of the cancer cells in the population (Figure 5D). The fraction of cancer cells that harbor an amp cannot be estimated from ctDNA data as the absolute number of DNA copies in such subclones are unknown. Thus, only considering the five cases without concurrent AR amps in ctDNA, we still found a resistance gap with no detectable resistance mechanism in 49%–100% of cancer cells sampled by ctDNA (Figure 5D). Although ctDNA and amplicon deep sequencing may not identify very small subclones with genetic resistance drivers due to sensitivity limits, we hypothesized based on the ctDNA results and the inability to define genetic AR drivers in 64% of biopsies from radiologically progressing mets, that non-genetic resistance mechanisms may exist.

### Transcriptomic Characteristics and Their Association with AR

**Figure 6:**
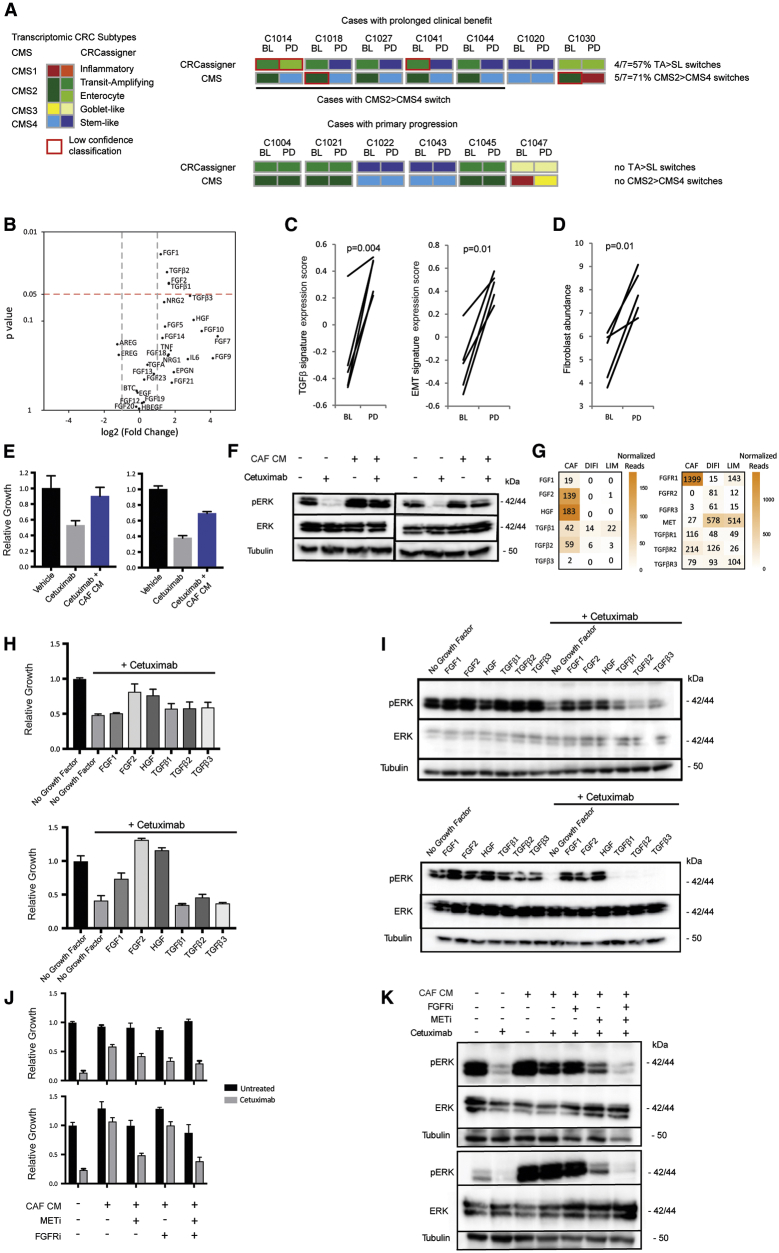
Transcriptomic CRC Subtypes and CAFs as Drivers of AR to Cetuximab (A) Transcriptomic subtypes in 13 BL and PD biopsy pairs. TA, transit amplifying; SL, stem-like. (B) Volcano plot showing differential expression of growth factors in 5 cases from (A) undergoing CMS2>4 switches. Significance was assessed by paired t test. (C) Changes in TGF-β and EMT transcriptomic signatures through CMS2>4 switches. (D) Changes in fibroblast abundance through CMS2>4 switches based on MCP-counter analysis. (E) Impact of CAF conditioned medium (CM) on the growth of DiFi (left panel) and LIM1215 (right panel) treated with 50 μg/mL CET for 5 days. (F) Western blot analysis showing CAF CM rescue of pERK in DiFi (left panel) and LIM1215 (right panel) treated with 200 μg/mL CET for 2 h. (G) mRNA expression (normalized counts) of growth factors (GFs) (left panel) and their receptors (right panel) in CAF, DiFi, and LIM1215 cells. (H) Growth assay with 200 μg/mL CET and recombinant GF at a concentration of 20 ng/mL (FGF1/2), 10 ng/mL (TGF-β) and 50 ng/mL (HGF) for 5 days in DiFi (top panel) and LIM1215 (bottom panel). (I) Western blot analysis of pERK with and without recombinant GF treatment in the presence or absence of 200 μg/mL CET in DiFi (top panel) and LIM1215 (bottom panel). (J) Growth assay with CAF CM and combinations of CET, pan-FGFR inhibitor (FGFRi), and MET inhibitor (METi) for 5 days in DiFi (top panel) and LIM1215 (bottom panel). (K) Western blot analysis of pERK after 2 h treatment with CAF CM and combinations of CET, FGFRi, and METi in DiFi (top panel) and LIM1215 (bottom panel). (E, H, and J) All error bars ± SD of six replicates. See also Figure S4 and Table S6.

Based on the observation that mechanisms of AR are often similar to those conferring primary resistance, we investigated whether transcriptomic subtypes have a role in AR. We first analyzed PD biopsies from tumors with prolonged benefit in which no genetic aberrations of CET resistance genes had been found. Strikingly, 5/7 cases (71%) showed a switch from the CET-sensitive CMS2 subtype to the CMS4 subtype (CMS2>4) and 4/7 (57%) showed a TA to stem-like (SL) subtype switch (TA > SL; Figures 6A and S1A). No CMS2/TA > CMS4/SL switches occurred in six pts with primary PD. CMS2>4 switching in the majority of PD biopsies without identifiable genetic resistance mechanisms suggested that this contributes to AR.

Transforming growth factor beta (TGF-β) expression is a defining characteristic of the CMS4/SL subtypes. TGF-β1 and TGF-β2 RNA expression significantly increased (3.1- and 2.9-fold increase in the means) following a CMS2>4 switch (Figure 6B). TGF-β3 mean expression increased 7.2-fold at PD but this did not reach significance. A high level of TGF-β activity in these samples was confirmed by the upregulation of a transcriptomic TGF-β signature and of an epithelial-to-mesenchymal transition (EMT) signature which can be induced by TGF-β (Figure 6C).

CMS4 CRCs are enriched with cancer-associated fibroblasts (CAFs), which are a major source of TGF-β and of mitogenic growth factors (GFs) (Becht et al., 2016a). Applying the MCP-counter algorithm (Becht et al., 2016b) to RNA-seq data bioinformatically confirmed a significant increase in CAF abundance in PD biopsies that had undergone a CMS2>4 switch (Figure 6D). Correspondingly, CMS2>4 subtype switches increased the expression of several GFs (Figure 6B), including FGF1 and FGF2 (2.3- and 3.1-fold increase in the means, respectively), which activate multiple FGFRs and of the MET ligand HGF, which increased 8.3-fold, although the latter was not significant. In contrast, the mean expression of the EGFR ligands AREG and EREG decreased 2.4- and 2.3-fold after subtype switching, but this was not significant.

Conditioned media (CM) from CAFs can confer CET resistance in CRC stem-like cells (Luraghi et al., 2014). We questioned whether CAFs also promote resistance in well-described CET-sensitive CRC cell lines. Treatment with CM from immortalized CRC CAFs indeed rescued growth and maintained pERK in DiFi and LIM1215 cells during CET treatment (Figures 6E and 6F). RNA-seq showed that CAFs expressed FGF1, FGF2, HGF, TGF-β1 and TGF-β2, and low levels of TGF-β3, and that the corresponding receptors were expressed in DiFi and LIM1215 cells (Figure 6G). Treatment of these cell lines with recombinant FGF1, FGF2, or HGF maintained growth and pERK during CET exposure (Figures 6H and 6I), whereas TGF-β1-3 had no consistent impact. We next assessed whether inhibitors of the corresponding GF receptors in combination with CET can reverse the resistance induced by CAF CM (Figures 6J and 6K). Combination of CET with FGFRi had minimal impact on pERK and cancer cell growth, whereas combination with a MET inhibitor (METi) showed a clear reduction of both. However, only the triple combination of CET with FGFRi and METi effectively repressed pERK and achieved the largest decrease in cancer cell growth during CAF CM treatment. Thus, FGF and HGF both contribute to CAF-mediated CET resistance.

Although these results support CMS2>4 switches and the associated increase in CAFs and mitogenic GF as a mechanism of CET AR, BL biopsies from two pts who subsequently achieved prolonged benefit from CET also displayed the CMS4 subtype. Thus, CMS4 identity does not invariably confer resistance. RNA-seq data from BL and PD biopsies were available from one of these cases (C1020) and showed that TGF-β2 (4.4-fold), TGF-β3 (4.2-fold), HGF (2.7-fold), and FGF2 (1.6-fold) all increased from BL to PD (Table S6). This suggests a model where a gradual increase in GF expression in a process associated with CAF infiltration and the acquisition of the CMS4 subtype promotes resistance.

This can evolve concurrently with genetic resistance in distinct subclones within the same pt, as demonstrated for cases that acquired CMS4 in a biopsy, whereas ctDNA showed the evolution of genetic resistance drivers, including RAS/RAF mutations, in subclones (C1027, C1041, and C1044). As anticipated, the triple combination of CET, METi, and FGFRi could not suppress the growth of *RAS-* or *BRAF*-mutant cell lines (Figure S4). The parallel evolution of molecularly diverse resistance mechanisms within pts, including currently undruggable *RAS* mutations, hinders the development of signaling pathway-targeting strategies to prevent or reverse resistance. The identification of new therapeutics that apply distinct selection pressures is hence a major need.

### CET Impacts the Cancer Immune Landscape

CET triggered immunogenic cell death and increased CRC immunogenicity in murine models (Pozzi et al., 2016). Yet, whether CET promotes CRC immune responses in pts is unclear. We investigated this to explore potential opportunities to target CET-resistant CRCs with immunotherapy.

**Figure 7:**
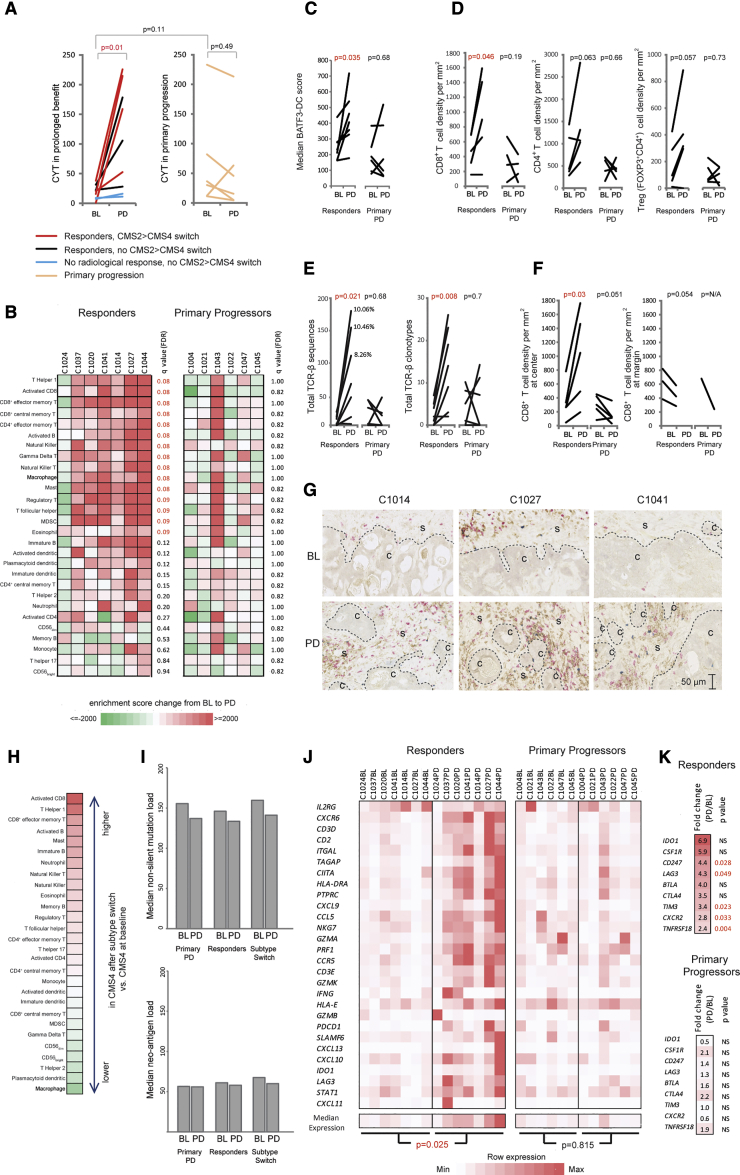
Impact of CET on the Tumor Immune Landscape (A) Cytolytic activity (CYT) change in paired BL and PD biopsies. (B) Single sample gene set enrichment analysis enrichment-score change for 28 immune cell subtypes from BL to PD. (C) Transcriptomic score estimating the abundance of BATF3^+^ dendritic cells (BATF3-DC). (D) Immuno-histochemical quantification of immune cell densities in formalin-fixed paraffin-embedded specimens. (E) Changes in the number of T cell receptor beta chain (TCR-β) sequences (left) and of clonotypes (right) from BL to PD. Percentages indicate the abundance of the largest TCR-β clonotype in samples with ≥100 TCR-β sequences. (F) Analysis of immune cell densities in the tumor center and at the margin in slides from (D). (G) Example of immune infiltrates before and after CMS2>4 subtype switches (red, CD8; brown, CD4; blue, FOXP3; C, cancer cell area; S, stroma). (H) Differences in immune cell abundance in biopsies that acquired CMS4 following a subtype switch and biopsies showing CMS4 at BL. Values were generated by subtracting median enrichment scores between the two groups. Higher abundance following CMS2>4 switch in red, lower abundance in green; color scale as in (B). (I) Median mutation and neoantigen loads (based on NetMHC rank <0.5%) at BL and PD. (J) Expression of a 28-gene T cell-associated inflammation signature. (K) RNA expression changes of targetable immune checkpoints and cytokine receptors. Statistical significance was assessed with the Mann-Whitney test followed by false discovery rate correction in (B) and with the paired Student's t test in all other panels. See also Figure S5.

We first applied the cytolytic activity (CYT) signature (Rooney et al., 2015), which estimates the abundance of cytotoxic immune cells from RNA-seq data (Figure 7A). The CYT did not differ between BL biopsies from tumors with prolonged benefit versus those with primary progression (Figure 7A). However, the mean CYT increased 5.9-fold from BL to PD in CRCs with prolonged benefit but not in those with primary progression, demonstrating that effective CET treatment increased cytotoxic immune infiltrates. CYT remained low in two tumors with prolonged benefit that showed no radiological shrinkage (C1018 and C1030), suggesting that cancer cell death induction is required to stimulate cytotoxic infiltrates. The largest CYT increases occurred in cases that switched from the CMS2 to the CMS4 subtype, which is associated with an inflamed phenotype (Guinney et al., 2015). However, the median CYT in PD biopsies of the five cases that switched to the CMS4 subtype was still 3-fold higher than in the five BL biopsies classed as CMS4 before CET exposure. Hence, increased CYT after CET therapy cannot be attributed to transcriptomic subtype changes alone.

Next, we bioinformatically inferred the abundance of 28 immune cell types from RNA-seq data (Charoentong et al., 2017). A significant increase in T cells that promote and execute adaptive immune responses, including all assessed CD8^+^ T cell subtypes, effector memory CD4^+^ and T helper type 1 (Th1) cells, was observed in PD biopsies taken after CET responses (Figure 7B). Some immune cell types that can dampen effective cancer immune responses, including regulatory T cells (Tregs) and myeloid-derived suppressor cells (MDSCs), also significantly increased. In contrast, immune cell infiltrates did not change in primary progressors. The presence of BATF3^+^ dendritic cells (DCs), which cross-present antigens from dying cancer cells to CD8^+^ T cells, is critical for immunotherapy efficacy in melanoma (Spranger et al., 2015). Applying a BATF3^+^ DC score (Spranger et al., 2017) showed a 1.7-fold increase (p = 0.035) at PD in tumors that had responded to CET but no change in primary progressors (p = 0.68, Figure 7C). Thus, several critical cell types for effective recognition of tumors by the adaptive immune system are enriched in tumors that responded to CET.

To ascertain changes in immune infiltrates, we stained CD8^+^ and CD4^+^ T cells, and Tregs (FOXP3^+^CD4^+^) in paired BL and PD formalin-fixed paraffin-embedded biopsies available from five pts with prolonged benefit and from five primary progressors (Figure 7D). CD8^+^ T cell densities increased significantly at PD compared with BL (2.0-fold change in means, p = 0.047) in pts who responded to CET. CD4^+^ and Treg numbers increased, but this was not significant (1.9-fold, p = 0.057 and 2.2-fold, p = 0.063), possibly because of the small number of cases in this analysis. Thus, CET treatment promotes T cell infiltration of CRCs that respond and these are present at the time of progression.

We furthermore assessed the number and diversity of rearranged T cell receptor beta chains (TCR-β) in RNA-seq data. A significant increase in the total number of TCR-β sequences and of distinct TCR-β clonotypes was apparent in PD samples of CET responders (Figure 7E), further validating the enrichment of T cells. The frequency of TCR-β clonotypes could only be assessed in three PD biopsies from CET responders because all other samples had insufficient total numbers of TCR-β sequences (<100). Although this needs to be interpreted with caution because of the small number of biopsies and TCR-β reads, the frequencies of the most abundant clonotype were between 8% and 10%, which may indicate that an oligoclonal T cell expansion occurred. B cell receptor chains showed a numerical increase at PD in CET responders but this was not significant (Figure S5A).

Our results show an increase in Th1 and CD8^+^ T cell infiltrates and CYT despite the high TGF-β levels in tumors that had undergone a CMS2>4 switch. This appears to contradict observations that show an important role of TGF-β in preventing T cell activation and differentiation in CRCs (Tauriello et al., 2018), and T cell migration into other tumor types (Mariathasan et al., 2018). To elucidate this further, we applied an approach similar to the CRC Immunoscore (Angelova et al., 2018), which assesses T cell infiltrates separately at the margin and in the tumor center. The tumor center could be identified in all paired biopsies from Figure 7D and margins were present in three paired biopsies from responders and in one from a primary progressor. CD8^+^ T cell infiltrates had specifically increased in the tumor center, whereas their density at the margin remained largely unchanged (Figures 7F and 7G). CD4^+^ T cells and Treg also predominantly increased in the tumor center, but this was not significant (Figure S5B). Comparison of immune cell infiltrates furthermore showed that activated CD8^+^, effector memory T cells, and Th1 cells most strongly increase and that Th2 subtype T cells are among the most strongly decreased in biopsies that switched from CMS2 to CMS4 compared with those showing the TGF-β-rich CMS4 subtype at BL (Figure 7H). Together, this suggests that the immune inhibitory effects of a TGF-β-rich environment may be less impactful following CET treatment than in untreated tumors (Tauriello et al., 2018). Importantly, tumor mutation load and neoantigen burden did not significantly differ between BL and PD biopsies, suggesting that the increase in T cell infiltrates was not the result of an increased antigenicity following CET exposure (Figure 7I).

We furthermore applied a signature of T cell-associated inflammation that is predictive for immune checkpoint inhibitor benefit in several cancer types (Ayers et al., 2017). This significantly increased from BL to PD in responders but not in primary progressors (Figure 7J). Effective CET therapy hence not only augments immune infiltrates including cytotoxic T cells, but also T cell-associated inflammation which may indicate enhanced T cell recognition of cancer cells. We finally questioned whether changes in immune infiltrates were accompanied by altered expression of immune checkpoints or chemokine receptors that can be targeted by current immunotherapy agents. The immune checkpoint proteins LAG3, PD-L1, TIM3, and GITR and the chemokine receptor CXCR2, which promotes myeloid cell infiltration, were significantly upregulated (Figure 7K). The upregulation of immune checkpoints may restrain T cell infiltrates and could provide opportunities to develop novel therapeutic strategies following CET failure.

## Discussion

This prospective trial revealed associations of biallelic *NF1* loss and of non-canonical RAS/RAF aberrations with primary resistance to single-agent CET. While *KRAS* A18D and L19F, and *BRAF* mutations other than V600E were rare in large CRC cohorts (each <1%) (Giannakis et al., 2016, TCGA, 2012), *NF1* mutations have been reported in ∼5% of cases and successful validation as a predictive marker in randomized trials could spare these pts ineffective treatment. Our results are supported by a study describing an association of *NF1* mutations with poor PFS with CET in combination with chemotherapy (Mei et al., 2018), although 3/4 were missense mutations with unknown effects on *NF1* function and there was no testing for loss of heterozygosity.

In contrast to previous reports (Bertotti et al., 2015, De Roock et al., 2010), neither *PIK3CA* mutations nor *FGFR1* aberrations clearly associated with primary resistance. *PIK3CA* exon 20 mutations have been particularly described to confer resistance to anti-EGFR-Ab in combination with chemotherapy; however, we found the exon 20 mutation H1047R in a responder but also in combination with a *PIK3CA* amp in a primary progressor. Concomitant copy-number aberrations or the use of single-agent CET may explain these differences. The small sample size furthermore warrants cautious interpretation of these results.

We found a strikingly lower frequency of AR driver mutations in RAS and *EGFR* in PD biopsies than anticipated based on the pervasive detection of these drivers in ctDNA from CET-treated pts (Bettegowda et al., 2014). The absence of CET resistance driver gene aberrations in 64% of PD biopsies was corroborated by ctDNA analysis, which did not detect AR drivers in 49%–100% of the sampled cancer cell population. This challenges the current paradigm that CET AR is almost exclusively mediated by genetic mechanisms. The majority of PD biopsies without identifiable genetic resistance drivers no longer displayed the CET-sensitive CMS2/TA subtype found before treatment initiation but rather the CMS4/SL subtype, which is rich in fibroblast and in GF, which conferred CET resistance *in vitro*. This strongly suggests that subtype switching and associated stromal remodeling is a mechanism of AR to single-agent CET. This could explain similar genetic results in a series of 37 PD biopsies that found no aberrations in RAS, *BRAF*, or *EGFR* in 46% of biopsies with anti-EGFR-Ab AR (Arena et al., 2015) and in a study of 22 pts in whom no genetic AR driver was found in 41% of biopsies, and those detected in the remaining biopsies were frequently subclonal (Pietrantonio et al., 2017).

These data demonstrate the limitations of ctDNA analysis, which is restricted to the identification of genetic resistance mechanisms and the importance of parallel tissue analyses with multi-omics approaches. They furthermore portray a CET resistance landscape resembling that of EGFR inhibitors in lung cancer or BRAF inhibitors in melanoma where non-genetic resistance can occur. Lung cancers can upregulate GFs that activate bypass signaling pathways or EMT as non-genetic resistance mechanisms (Sequist et al., 2011, Soucheray et al., 2015, Zhang et al., 2012) and fibroblast-mediated stromal remodeling can confer AR to BRAF inhibitors in melanoma (Hirata et al., 2015).

We showed that resistance induced by CAF CM or by FGF10 can be reversed through drug combinations *in vitro*. However, combinatorial drug treatments are challenging in pts, due to likely toxicities when attempting to combine multiple signaling pathway inhibitors and because of the inability to effectively target RAS mutant clones that evolved in 4/9 pts. However, strategies to delay resistance by preventing subtype switching, for example by inhibiting TGF-β, a master regulator of the CMS4/SL subtype, or by targeting CAFs (Kalluri, 2016) could be assessed.

Our analysis of the immune landscape in CRCs that responded to CET and then progressed shows significantly increased cytotoxic T cells but also of immune-suppressive cells, such as Tregs and MDSCs. This was accompanied by the upregulation of a signature that has been predictive of checkpoint inhibitor success in other cancer types, potentially indicating a role for immunotherapy. The significant upregulation of immune-suppressive checkpoints, such as PD-L1 and LAG3, defines testable strategies.

The paradoxical increase in immune infiltrates following CMS2>4 switches, despite the TGF-β-rich CMS4 phenotype, may be explained by the context-dependent effects of TGF-β and by the timing of events: TGF-β has been well documented to prevent differentiation of naive CD4^+^ T cells into Th1 and Th2 cells, and naive CD8^+^ T cells into cytotoxic T cells (Li and Flavell, 2008, Li et al., 2006). However, our data show low TGF-β expression in pre-treatment biopsies. It is likely that immunogenic cell death fosters T cell activation, priming and infiltration before resistance-associated stromal remodeling and the associated increase in TGF-β occur. The observed increase in CYT in tumors that underwent a CMS2>4 switch suggests that T cells remain active in the tumor. This can be explained by previous work demonstrating that TGF-β has little effect on activated T cells (Cottrez and Groux, 2001, Kim et al., 2005, Sung et al., 2003). Nevertheless, combining checkpoint and TGF-β inhibitors in clinical trials would be a rational strategy to test if inhibitory effects of TGF-β (Tauriello et al., 2018) still play a role.

Investigating how CET modulates CRC immune landscapes in additional trials is desirable, because tissue attrition, which is typical in biopsy studies, limited the number of cases amenable to immunophenotyping in this trial. Assessing larger series of CET-treated CRCs with multi-parametric immunofluorescence imaging could furthermore define the spatial distribution of various immune cell subtypes and the relationship to cells producing immune inhibitory cytokines in greater detail. A key result of our study is that drugs that are in routine clinical use can have a major impact on cancer immune landscapes. Mouse models such as those described by Tauriello et al. (2018) offer the opportunity to systematically investigate such interactions further and to delineate the role of cytokines and cell subtypes that are currently difficult to target in pts, such as Tregs or MDSCs. Exploring immunotherapies in CET-resistant CRCs may circumvent the limited clinical opportunities to directly target the frequently polyclonal and heterogeneous CET resistance mechanisms.

## STAR★Methods

### Key Resources Table

**Table:** 

REAGENT or RESOURCE	SOURCE	IDENTIFIER
**Antibodies**		

p-ERK	Cell Signaling Technologies	Cat# 9101; RRID:AB_331646
ERK	Cell Signaling Technologies	Cat# 9102; RRID:AB_330744
p-EGFR	Cell Signaling Technologies	Cat# 2236; RRID:AB_331792
EGFR	Cell Signaling Technologies	Cat# 2232; RRID:AB_331707
NF1	Cell Signaling Technologies	Cat# 14623
beta Tubulin antibody (HRP) - Loading Control	Abcam	Cat# ab21058; RRID:AB_727045
Cas9	Cell Signaling Technologies	Cat# 14697; RRID:AB_2750916

**Biological Samples**		

Human Metastatic Colorectal Cancer Patient Saples	PROSPECT-C Trial	clinicaltrials.gov/ct2/show/NCT02994888

**Chemicals, Peptides, and Recombinant Proteins**		

Cetuximab	Merck KG	
AMG-337	Selleckchem	Cat# S8167
BGJ-398	Selleckchem	Cat# S2183
Recombinant human FGF1 acidic	RnD Systems	Cat# 232-FA-025
Recombinant human FGF2 basic	RnD Systems	Cat# 233-FB-025
Recombinant human TGFβ1	RnD Systems	Cat# 240-B-002
Recombinant human TGFβ2	RnD Systems	Cat# 302-B2-002
Recombinant human TGFβ3	RnD Systems	Cat# 243-B3-002
Recombinant human FGF10	PeproTech	Cat# 100-26
Recombinant human HGF	PeproTech	Cat# 100-39H
Polybrene	Sigma-Aldrich	Cat# H9268
Blasticidin	Sigma-Aldrich	Cat# 15205
Puromycin	Sigma-Aldrich	Cat# P8833
Neomycin	Sigma-Aldrich	Cat# N1142
Phosphatase Inhibitor Cocktail 2	Sigma-Aldrich	Cat# P5726

**Critical Commercial Assays**		

All Prep DNA/RNA Micro Kit	Qiagen	Cat# 80284
GenePrint 10 kit	Promega	Cat# B9510
QIAamp DNA Blood Mini Kit (50)	Qiagen	Cat# 51104
Qubit dsDNA Broad Range Assay Kit	Thermo Fisher Scientific	Cat# Q32850
QIAmp Circulating Nucleic Acid Kit	Qiagen	Cat# 55114
SureSelectXT Human All Exon v5 Kit	Agilent	Cat# 5990-9857
NEBNext® Ultra™ Directional RNA Library Prep Kit	New England Biolabs	Cat# E7420S
RNeasy Mini Kit	Qiagen	Cat# 74104
Qubit RNA High Sensitivity Kit	Thermo Fisher Scientific	Cat# Q32852
QuantSeq 3’ mRNA-Seq Library Preparation Kit for Illumina (FWD)	Lexogen	Cat# 015.96
Bioanalyzer High Sensitivity DNA	Agilent	Cat# 5067-4626
CellTiter-Blue	Promega	Cat# G8080
QuikChange Lightning	Agilent	Cat# 5990-8816
TransIT-LT1	Mirus	Cat# MIR 2300
Lipofectamine 2000	Thermo Fisher Scientific	Cat# 11668019
siGenome	Dharmacon	
Lipofectamine™ RNAiMAX	Thermo Fisher Scientific	Cat# 13778030

**Deposited Data**		

Sequencing Data (Whole Exome, Whole Genome, mRNA, Targeted ctDNA)	European Genome-Phenome Archive	EGA: EGAS00001003367

**Experimental Models: Cell Lines**		

DIFI	N. Valeri, ICR	
LIM 1215	N. Valeri, ICR	
NIH-3T3	P. Huang, ICR	
HT29	ATCC	Cat# HTB-38 RRID:CVCL_0320
SW480	ATCC	Cat# CCL-228 RRID:CVCL_0546
SW620	ATCC	Cat# CCL-227 RRID:CVCL_0547
Human fibroblasts from rectal carcinoma (RC11)	D. Vignjevic, Institute Curie, France	

**Oligonucleotides**		

See Table S7 for details.		

**Recombinant DNA**		

R777-E053-Hs.EGFR	Addgene	Cat# 70337 RRID:Addgene_70337
R777-E015-Hs.BRAF	Addgene	Cat# 70299 RRID:Addgene_70299
R777_E087-Hs.FGFR3	Addgene	Cat# 70371 RRID:Addgene_70371
pDONOR223_BRAF_p.D594H	Addgene	Cat# 82816 RRID:Addgene_82816
KRAS (NM_004985) Human Tagged ORF Clone	OriGene	Cat# RC201958
pENTR1A	Invitrogen	Cat# A10462
pLX304	Addgene	#25890 RRID:Addgene_25890
pLenti-CMV-Puro-DEST	Addgene	Cat# 17452 RRID:Addgene_17452
pEZY3	Addgene	Cat# 18672 RRID:Addgene_18672
pLX304-LacZ	S.Whittaker	
pLenti-CMV-Puro-LUC	Addgene	Cat# 17477 RRID:Addgene_17477
pEZYegfp	Addgene	Cat# 18671 RRID:Addgene_18671
psPAX2	Addgene	Cat# 12260 RRID:Addgene_12260
pMD2.G	Addgene	Cat# 12259 RRID:Addgene_12259
lentiCas9-Blast	Addgene	Cat# 52962 RRID:Addgene_52962
pLentiguide-Puro	Addgene	Cat# 52963 RRID:Addgene_52963

**Software and Algorithms**		

BWA v0.7.12	Li and Durbin, 2009	http://bio-bwa.sourceforge.net
Picard v2.1.0		https://broadinstitute.github.io/picard/
SAMtools v0.1.19	Li et al, 2009	http://samtools.sourceforge.net
GATK v3.5-0	McKenna et al., 2010	https://software.broadinstitute.org/gatk/
fastqc v0.11.4		https://www.bioinformatics.babraham.ac.uk/projects/fastqc/
MuTect v1.1.7	Cibulskis et al., 2013	https://software.broadinstitute.org/cancer/cga/mutect
VarScan2 v.2.4.1	Koboldt et al., 2012	http://varscan.sourceforge.net
BAM-readcount V0.7.4		https://github.com/genome/bam-readcount
fpfilter.pl	Koboldt et al., 2013	https://github.com/genome/fpfilter-tool
Platypus v0.8.1	Rimmer et al., 2014	https://github.com/andyrimmer/Platypus
Annovar v20160201	Wang et al., 2010	http://annovar.openbioinformatics.org/en/latest/
CNVKit v0.8.1	Talevich et al, 2016	https://cnvkit.readthedocs.io/en/stable/
SnpEff v4.2	Cingolani et al, 2012	http://snpeff.sourceforge.net
Sequenza v2.1.2	Favero et al, 2015	http://cbs.dtu.dk/biotools/sequenza/
Bcl2fastq v1.8.4		http://emea.support.illumina.com/sequencing/sequencing_software/bcl2fastq-conversion-software.html
Tophat2 v2.0.7	Kim et al., 2013	https://ccb.jhu.edu/software/tophat/index.shtml
Bowtie2 v2.1.0	Langmead and Salzberg, 2012	http://bowtie-bio.sourceforge.net/bowtie2/index.shtml
Rsubread FeatureCounts v1.24.2	Liao et al, 2014	http://bioinf.wehi.edu.au/featureCounts/
DESeq2 v1.18.1	Love et al, 2014	https://bioconductor.org/packages/release/bioc/html/DESeq2.html
CMScaller v0.99.1	Eide et al, 2017	https://github.com/peterawe/CMScaller
CRCAssigner	Sadanandam et al., 2013	
MCP-counter v1.1.0	Becht et al., 2016b	https://github.com/ebecht/MCPcounter
Limma v3.34.9	Ritchie et al., 2015	https://bioconductor.org/packages/release/bioc/html/limma.html
MiXCR v3.0.5	Bolotin et al, 2015	https://mixcr.readthedocs.io
VEP	McLaren et al, 2016	https://www.ensembl.org/info/docs/tools/vep/index.html
Polysolver v1.0d	Shukla et al., 2015	https://software.broadinstitute.org/cancer/cga/polysolver
netMHCpan-4.0	Jurtz et al., 2017	http://www.cbs.dtu.dk/services/NetMHCpan/
deconstructSigs v1.8.0	Rosenthal et al., 2016	https://github.com/raerose01/deconstructSigs
Ion Torrent Suite v5.2.2	ThermoFisher Scientific	
SureCall v4.0.1.45	Agilent	
FWD Human (GRCh38) Lexogen QuantSeq 2.2.3	BlueBee Cloud	https://www.bluebee.com
Lexogen QuantSeq DE 1.3.0	BlueBee Cloud	https://www.bluebee.com
QuikChange Primer Design (Lightning)	Agilent	https://www.agilent.com/store/primerDesignProgram.jsp

### Contact for Reagent and Resource Sharing

Further information and requests for resources and reagents should be directed to and will be fulfilled by the Lead Contact, Marco Gerlinger (marco.gerlinger@icr.ac.uk). DNA and RNA sequencing data have been deposited in the European Genome Phenome short read archive and access can be obtained after signing a material transfer agreement which protects patient confidentiality and prohibits any attempts to re-identify patients.

### Experimental Models and Subject Details

#### Trial Design and Samples

The Prospect-C trial is a prospective translational study investigating biomarkers of response or resistance to anti-EGFR-Ab-therapy in *KRAS* WT chemo-refractory metastatic CRC. No *NRAS* mutant cases were enrolled as the licensed cetuximab (CET) indication changed to *KRAS* and *NRAS* WT CRC during the trial. Pts who were at least 18 years old and had a World Health Organization performance status of 0-2, were eligible if: all conventional treatment options including fluorouracil, irinotecan, oxaliplatin were exhausted or pts were intolerant/had contraindications for oxaliplatin/irinotecan-based chemotherapy; they had metastatic cancer amenable to biopsy and repeat measurements with computed tomography (CT) scanning. See Table S1 for pts characteristics including gender and age.

Written informed consent was obtained from all pts. The study was carried out in accordance with the Declaration of Helsinki and approved by the national UK ethics committee (UK Research Ethics Committee approval: 12/LO/0914). All participants were required to have mandatory image-guided pre-treatment biopsies (targeted to the CT identified index lesion), and mandatory biopsies at the time of RECIST-defined progression (from one or 2 suitable progressing metastatic sites). Treatment consisted of single-agent CET at a dose of 500 mg/m^2^ administered every other week until progression or intolerable side effects.

The identification of biomarkers of primary and acquired resistance to CET therapy in DNA and RNA from CRC tumor biopsies was the primary endpoint of the study. The study recruited to the recruitment target of 30 pts that had been treated and had BL and PD samples available for genetic analyses. After removing cases with insufficient DNA yield or tumor content based on sequencing results, data from 24 paired BL and PD samples was available for mutation and copy number analysis. 11 cases from which only a BL biopsy was available were included in the analysis. Secondary endpoints included the identification and validation of biomarkers for resistance and response to CET in RNA and ctDNA. The trial protocol also permitted further exploratory molecular analyses.

The efficacy parameters including partial response and stable disease were measured using RECIST v1.1 criteria. Progression free survival (PFS) was measured from start of treatment to date of progression or death from any cause. Overall survival (OS) was defined as time from start of treatment to death of any cause. Pts without an event were censored at last follow up before PFS and OS were estimated.

The cohort was dichotomized into primary progressors who had PD before or on the first per protocol CT scan, scheduled at 12 weeks from the start of CET treatment. This was performed at a median of 12 weeks with a range of 9-16 weeks on treatment. Pts with prolonged benefit were defined as those who remained progression free at the time of this scan. Samples from healthy donors were collected for ctDNA sequencing after obtaining written informed consent through the ‘Improving Outcomes in Cancer’ biobanking protocol at the Barts Cancer Centre (PI: Powles), which was approved by the UK national ethics committee (Research Ethics Committee approval: 13/EM/0327).

#### Cell Lines

DiFi and LIM1215 cell lines were a gift from the Valeri Lab at ICR. Mouse NIH-3T3 cells were a gift from the Huang Lab at ICR. HT29, SW480 and SW620 were obtained from ATCC. DiFi cells were cultured in RPMI-1640 (Gibco), GlutaMax (Gibco), 5% FBS. LIM1215 cells were cultured in RPMI-1640, 10% FBS, hydrocortisone (Gibco), 1-thioglycerol (Sigma) and insulin (Gibco). NIH-3T3 and HT29 cells were cultured in DMEM (Gibco), GlutaMax (Gibco) and 10% FBS. SW480 and SW620 were cultured in L15 (Gibco), GlutaMax (Gibco) and 10% FBS. Human fibroblasts from rectal carcinomas which have been immortalized using hTERT virus (pCSII vector backbone, RC11) were a gift from Fernando Calvo, initially provided by Danijela Vignjevic (Institute Curie, France)(Glentis et al., 2017). Fibroblasts were cultured in DMEM (Sigma), GlutaMax (Gibco), 10% FBS, 1% insulin-selenium-transferrin. All cell lines were grown at 37°C. RC11 was cultured at 10% CO_2_, DiFi, LIM1215, HT29 and NIH-3T3 were all cultured in 5% CO_2_ and SW480 and SW620 were cultured in 0% CO_2_. Human cell lines have been authenticated by STR profiling using the GenePrint 10 kit (Promega). The DiFi cell line has no available STR profile, but the cells were confirmed as identical at start and end of this study. DiFi and HT29 cell lines are female. LIM1215, SW480, SW620 and NIH-3T3 cell lines are male.

### Method Details

#### Sample Preparation

DNA and RNA were extracted simultaneously from snap frozen biopsies using the Qiagen All Prep DNA/RNA Micro Kit following the manufacturer’s instructions. Matched normal DNA was extracted from blood samples using the Qiagen DNA Blood Mini Kit. DNA concentration was measured using the Qubit dsDNA Broad Range Assay Kit, and integrity checked by agarose gel electrophoresis. A minimum quantity of 500 ng, and where available 2 μg of DNA, was used for next generation sequencing. RNA from biopsies which were successfully DNA sequenced was subjected to RNA-Sequencing if a sufficient quantity (>125 ng) and quality (RIN>5.5) was confirmed by electrophoresis on the Agilent 2100 Bioanalyzer. Blood for circulating tumor DNA analysis was collected in EDTA tubes and centrifuged within 2 hours (10 min, 1600g) to separate plasma, which was stored at −80°C. Upon thawing, samples were further centrifuged (10 min, 16000g, 4°C). ctDNA was extracted from up to 4 mL plasma per patient and from 2x4 mL from healthy donors using the Qiagen QIAamp Circulating Nucleic Acid Kit. ctDNA was quantified on the Agilent 2100 Bioanalyzer.

#### Whole Exome/Genome DNA Sequencing

Biopsy samples were sequenced by the NGS-Sequencing facility of the Tumour Profiling Unit at the Institute of Cancer Research (ICR) or at the Beijing Genome Institute (BGI). Exome sequencing libraries were prepared from a minimum of 500 ng DNA using the Agilent SureSelectXT Human All Exon v5 kit according to the manufacturer’s protocol. Paired-end sequencing was performed on the Illumina HiSeq 2000 or 2500 platform with a target depth of 100X for exomes (BGI/ICR) and on the Illumina HiSeq X10 platform with 70X for genomes (BGI).

#### Bioinformatics Analysis of DNA Sequencing Data

BWA-MEM (Li and Durbin, 2009) (v0.7.12) was used to align the paired-end reads to the hg19 human reference genome to generate BAM format files. Picard Tools (http://picard.sourceforge.net) (v2.1.0) *MarkDuplicates* was run with duplicates removed. BAM files were coordinate sorted and indexed with SAMtools (Li et al., 2009) (v0.1.19). BAM files were quality controlled using GATK (McKenna et al., 2010) (v3.5-0) *DepthOfCoverage*, Picard *CollectAlignmentSummaryMetrics* (v2.1.0) and fastqc (https://www.bioinformatics.babraham.ac.uk/projects/fastqc/) (v0.11.4).

#### Somatic Mutation Analysis

Tumor and germline DNA sequencing results were assessed for matching SNP profiles to check for potential sample swaps. This identified one case where germline DNA and tumor DNA SNP profiles differed and this was removed from the analysis. For single nucleotide variant (SNV) calls we used both MuTect (Cibulskis et al., 2013) (v1.1.7) and VarScan2 (Koboldt et al., 2012) (v2.4.1). SAMtools (v1.3) *mpileup* was run with minimum mapping quality 1 and minimum base quality 20. The pileup file was inputted to VarScan2 *somatic* and run with a minimum variant frequency of 5%. The VarScan2 call loci were converted to BED file format and BAM-readcount (https://github.com/genome/bam-readcount) (v0.7.4) run on these positions with minimum mapping quality 1. The BAM-readcount output allowed the VarScan2 calls to be further filtered using the recommended *fpfilter*.*pl* accessory script (Koboldt et al., 2013) run on default settings. MuTect was run on default settings and post-filtered for minimum variant allele frequency 5%. Indel calls were generated using Platypus (Rimmer et al., 2014) (v.0.8.1) *callVariants* run on default settings. Calls were filtered based on the following FILTER flags - ‘GOF, ‘badReads, ‘hp10,’ MQ’, ‘strandBias’,’ QualDepth’,’ REFCALL’. We then filtered for somatic indels with normal genotype to be homozygous, minimum depth ≥10 in the normal, minimum depth ≥20 in the tumor and ≥5 variant reads in the tumor. Exonic regions were analyzed in whole genome sequenced samples to assure comparability to the whole exome sequenced samples. Mutation calls were further filtered with a cross-normal filter by running bam-readcount on the bed file of merged variants for all sequenced matched normal (blood) samples. For both SNV and Indel calls we used a threshold of ≥2% of the total number of reads at the call loci. If the alternate allele count is equal to or greater than this threshold the variant is flagged as present in the normal sample. A call is rejected if the variant is flagged in 5% or more of the normal samples in our cohort to remove common alignment artifacts or those arising recurrently at genomic positions that are difficult to sequence.

Mutation calls were merged and annotated using annovar (Wang et al., 2010) (v20160201) with hg19 build version. The allele counts were recalculated using bam-readcount with minimum base quality 5 (in line with minimum default settings of the joint SNV callers). The calls were then filtered on minimum variant allele frequency ≥5%, minimum depth ≥20 in a called sample and a maximum of 2 variant alleles in the matched normal sample.

#### DNA Copy Number Aberration Analysis

CNVKit (Talevich et al., 2016) (v0.8.1) was run in non-batch mode for copy number evaluation. We first identified high confidence SNP locations using bcftools *call* (Li et al., 2009) (v1.3) with snp137 reference and SnpEff *SnpSift* (Cingolani et al., 2012) (v4.2) to filter heterozygous loci with minimum depth 50. We further extracted positions spaced 500 bp apart in the whole genome samples. VarScan2 was used to call the tumor sample BAMs at these locations to generate B-Allele Frequency (BAF) data as input for CNVKit.

We generated basic *access* and *antitarget* files to indicate the accessible sequence regions. This excluded blacklisted regions suggested by CNVKit and the HLA region. We then generated a pooled normal sample and used the *winsorize* and *pcf* functions within copynumber (Nilsen et al., 2012) to identify further outlier positions and regions of highly uneven coverage. These regions were merged to ensure consistency across all data.

CNVKit was run with matched normals along with the adjusted access and antitarget files. For the segmentation step we ran *pcf* from the R-package copynumber. Breakpoints from this segmentation step were then fed into Sequenza (Favero et al., 2015) (v2.1.2) to calculate estimates of purity/ploidy and these values were used as a guide to recenter and scale the LogR profiles in CNVKit. BAF and LogR profiles were also manually reviewed by 2 researchers to determine their likely integer copy number states. Adjustments were made in cases where both manual reviews identified a consensus solution that differed from the bioinformatically generated integer copy number profile. Furthermore, BL/PD sample pairs where the ploidy of one sample was close to double the ploidy of the other sample and copy number profiles were highly similar (suggestive of a genome doubling event), the sample with lower ploidy was adjusted to the likely genome-doubled higher state to facilitate a direct comparison of copy number changes, unless clear evidence of BAF and LogR profiles suggested otherwise. These adjustments were made in samples C1004PD, C1022PD, C1025PD, C1027PD1, C1030PD, and C1043BL where both manual reviews supported a different solution to Sequenza.

#### Analysis of Gene Amps

Amps were defined as a 3-fold or greater increase on the ploidy of a sample, a substantial loss event as a 3-fold or greater decrease on the ploidy state and a homozygous deletion as CN=0. Amp and loss threshold values were rounded to the nearest integer copy number state. Ploidy was estimated as follows,

$$\text{Ploidy}\;=\;\left({\text{CN}}_{\text{Absolute}}\times \text{SegmentLength}\right)/\sum \left(\text{SegmentLength}\right)$$

with CN_Absolute_ representing the unrounded copy number estimate and SegmentLength the genomic length between segment break points. BL and PD biopsy pairs were compared to identify which cases had acquired amps at PD that were absent at BL.

#### Deep Amplicon Sequencing

Ampliseq libraries were prepared by the ICR-TPU using the Ion Chef from 800 ng DNA extracted from BL/PD biopsies, and from matched germline samples. A custom amplicon panel comprising a single pool of 77 amplicons (Table S4 for amplicon positions) was designed to cover mutational hotspots and known CET resistance drivers in *KRAS*, *NRAS*, *BRAF*, *EGFR* and *MAP2K1* and several mutations identified by exome sequencing in each sample (including any *TP53* and *APC* mutations) to enable subclonality estimates. Up to 32 samples were pooled and sequenced on PGM 318 chips (v2) with 500 flows. Deep amplicon sequencing data was aligned and somatic mutations were called using the Ion Torrent Suite software (v5.2.2). run with a minimum variant frequency of 0.5% and 3 supporting variant reads.

#### ctDNA-sequencing

Ultra-deep circulating tumor DNA (ctDNA) sequencing with molecular barcode error correction (Mansukhani et al., 2018) was applied to cases with prolonged benefit from CET and which had at least 25 ng of ctDNA. Libraries were prepared from 25 ng ctDNA using the Agilent SureSelect^XT-HS^ kit and hybridized to a CRC panel targeting up to 40 genes (Table S4) using our optimized protocol (Mansukhani et al., 2018). Libraries were pooled and sequenced on an Illumina HiSeq2500 in 75 bp paired-end mode, generating a median of 125.7 M reads/sample.

The resulting data was aligned and molecular barcode-deduplicated in order to reduce false positive sequencing errors using Agilent SureCall, with variants called using the integrated SNPPET caller. To call very low frequency variants, bam-readcount was used to interrogate targeted hotspot positions in *KRAS*, *NRAS*, *BRAF*, *MAP2K1* and *EGFR* (Table S4). In order to maximize the sensitivity for the detection of resistance mutations, these were called if at least 2 independent variant reads were identified at a mutational hotspot position and encoded for a recurrently observed amino acid change in the specific gene. Genome-wide copy number profiles were constructed using CNVKit run in batch mode with Antitarget average size 30 kb as described (Mansukhani et al., 2018). ctDNA sequenced from healthy donors (Mansukhani et al., 2018) was used as the normal reference dataset. Copy number profiles generated from ctDNA were aligned with copy number profiles showing absolute copy numbers from matched biopsies and the closest integer copy number was assigned to *TP53* and mutated CET resistance driver genes for the subclonality analysis.

#### RNA-sequencing of Biopsies

NEB polyA kit was used to select the mRNA. Strand specific libraries were generated from the mRNA using the NEB ultra directional kit. Illumina paired-end libraries were sequenced on an Illumina HiSeq2500 using v4 chemistry acquiring 2 x 100 bp reads. Bcl2fastq software (v1.8.4, Illumina) was used for converting the raw basecalls to fastq format and to further demultiplex the sequencing data.

Tophat2 spliced alignment software (Kim et al., 2013) (v2.0.7) was used to align reads to the GRCh37 (hg19) release-87 human reference genome in combination with Bowtie2 (Langmead and Salzberg, 2012) (v2.1.0). FeatureCounts (Liao et al., 2014) was used to perform read summarization. Sample QC was performed using Picard Tools *CollectRnaSeqMetrics*. We excluded 2 samples (C1006BL and C1007BL) with fewer than 10% of reads aligning to exonic regions. Lowly expressed genes were filtered using a cpm threshold equivalent to 10/L, where L is the minimum library size in millions (Chen et al., 2016). Sample batch effects were assessed using principal component analysis and did not require corrective action. Counts were normalized for library size using *estimateSizeFactors* in Deseq2 (Love et al., 2014). FPKM data were generated using the *fpkm* function in Deseq2. For downstream analysis all data were filtered for protein coding genes using the GTF annotation file and filtering on the *gene_biotype* column.

#### RNA Sequencing of Cell Lines and CAFs

RNA was extracted using the Qiagen RNeasy kit and quantified using Qubit RNA High Sensitivity kit. 224 ng RNA was used as input for Lexogen QuantSeq 3’ mRNA-Seq Library Preparation kit for Illumina (FWD), and libraries were prepared according to the manufacturer’s protocol, with optimal 15 cycles of PCR determined by qPCR. Final libraries were quantified with both Qubit and Bioanalyzer DNA High Sensitivity kits and equimolar pools were sequenced on an Illumina HiSeq2500 in Rapid 100 bp single-end mode with dual indexing, generating a median of 7.2 M reads per sample. Sequencing data was analysed using the FWD Human (GRCh38) Lexogen QuantSeq 2.2.3 and Lexogen QuantSeq DE 1.3.0 pipelines on the BlueBee cloud platform.

#### Cancer Cell Content Analysis

The cancer cell content of each sequenced sample was assessed based on the variant allele frequency (VAF) of somatic mutations and samples with an estimated cancer cell content below 10% were removed from the analysis as the sequencing depth was insufficient to accurately detect mutations in these samples (Cibulskis et al., 2013). As the majority of mutations are heterozygous and hence present in half of the DNA copies of the cancer cells, 2xVAF can be used to approximation the fraction of cancer cells in a sample. This led to the exclusion of 4 samples (C1001BL, C1009BL, C1010BL, C1042BL) as shown in the CONSORT diagram (Figure 1A). The median estimated cancer cell content across the remaining 60 samples was 41% (Table S1).

#### Subclonality Analysis Exome Sequencing Data

The clonal status of mutations was assessed using the allele specific copy number generated in the CNVKit solution. We estimated the cancer cell fraction (CCF) using the phyloCCF method as described (Jamal-Hanjani et al., 2017). We then inferred the mutation copy number (i.e. the number of alleles harboring the mutation) and assigned clonal/subclonal status to each variant using the criteria described by McGranahan et al. (McGranahan et al., 2015).

#### Subclonality Analysis in ctDNA and Amplicon Sequencing Data

Variant allele frequencies of *TP53* mutations, of hotspot resistance driver mutations in *KRAS*, *NRAS*, *BRAF* and *EGFR* and of the *EGFR* mutation D278N were extracted from ctDNA BAM files. *TP53* mutation VAFs were used to calculate what fraction of the ctDNA was of cancer cell origin by correcting for the influence of copy number aberrations using the following formula:

$$\text{CCF}\;=2^{*}\text{VAF}/\left({\text{Copies}}_{\text{mutated}}+\;2^{*}\text{VAF}\;-{{\text{VAF}}^{*}\text{Copies}}_{\text{total}}\right)$$

with CCF indicating the cancer cell fraction, Copies_mutated_ the number of copies that harbored the *TP53* mutation and Copies_total_ the absolute copy number of the *TP53* locus. Clonality analysis of *TP53* mutation showed clonal mutations and loss of heterozygosity of the *TP53* locus for all tumor biopsies with the exception of C1027 which harbored 2 *TP53* mutations, one present on 4 copies of chromosome 17p and one on 2 copies, suggesting biallelic inactivation through 2 distinct mutation events. *TP53* Copies_mutated_ and Copies_total_ were equal for tumors with *TP53* LOH and in 1027 the VAFs of both *TP53* mutations were taken together and the sum of all chromosome 17p copies were used to estimate CCF.

The same formula was then resolved to calculate the expected VAF of a clonal mutation given the CCF of the ctDNA sample and the local copy number state of this mutation:

$$\text{VAF}\;=\left({{\text{CCF}}^{*}\text{Copies}}_{\text{mutated}}\right)/\left({{\text{CCF}}^{*}\text{Copies}}_{\text{total}}+2-2^{*}\text{CCF}\right)$$

Copies_total_ for all mutations were inferred from ctDNA copy number profiles that had been close matched to the integer copy number states of biopsies (Data S3). For subclonality calculation, we furthermore assumed that resistance drivers were only mutated on a single gene copy (i.e. Copies_mutated_=1, which is likely as they are thought to have a dominant effect). This assumption furthermore maximized the estimated fraction of cancer cells that harbor a resistance driver mutation, hence providing a conservative measure of the resistance gap. The fraction of the total CCF in ctDNA that harbors an observed resistance driver mutation was then calculated by dividing the observed VAF by the expected VAF for a mutation that is 100% clonal. We then estimated the maximum fraction of all cancer cells that harbored resistance driver mutations in a sample as the sum of the CCF values of all individual resistance driver mutations in that sample.

#### Colorectal Cancer Subtyping

Consensus Molecular Subtypes (Guinney et al., 2015) were assigned using CMScaller (Eide et al., 2017). The CMScaller function was run with raw count data and setting ‘RNASeq=TRUE’. Each sample was assigned the subtype with the shortest distance according to the inbuilt nearest template prediction (NTP) (Hoshida, 2010). The CMScaller classification was considered low confidence if FDR >0.01. Samples were also assigned to the molecular CRC subtypes, as described (Sadanandam et al., 2013). To minimize technical differences in subtype assignment we generated data normalized using the same approach as CMScaller (limma::normalizeQuantiles(log2(x+.25))). The data were then row median centered and correlated with the PAM centroids, as defined by the published 786-gene CRCassigner signature. Each sample was then assigned to the CRC subtype with the highest correlation. If the correlation coefficient is <0.15 or the difference with the second highest coefficient is <0.06 then the sample is considered low confidence (Guinney et al., 2015). The EMT and TGFβ expression signatures were generated by the Camera Gene Set Analysis in CMScaller for each sample.

The subtyping showed a high level of agreement between the classification approaches. This was true even of assignments considered low confidence by the published criteria.

#### Immune Cell Infiltrate Analysis

The cytolytic activity (CYT) was calculated as the geometric mean of the *GZMA* and *PRF1* genes (normalized expression values as input, offset by 1.0). The BATF3-DC signature was calculated as the mean of the normalized expression values of the genes in this signature. FPKM normalized RNA sequencing data and published immune cell metagenes (Charoentong et al., 2017) were used as input into the single sample gene set enrichment analysis (ssGSEA) algorithm using default settings to determine immune cell enrichments in each sample as described (Barbie et al., 2009).

The Microenvironment Cell Populations (MCP)-counter algorithm (Becht et al., 2016b) was used as an independent bioinformatics tool to assess immune cell enrichment. Data were normalized using limma *voom* (Ritchie et al., 2015) and the MCP-counter function run with *HUGO_symbols* chosen as *featuresType*.

#### Quantifying Clonotypes for T and B Cell Populations

MiXCR (v3.0.5) (Bolotin et al., 2015) was used to extract B and T cell receptor repertoire data from RNA-seq data using the ‘analyze shotgun’ command, selecting for ‘--starting-material rna’, ‘—species hs’ and ‘–only-productive’. Data were exported for T cell receptor β and B cell heavy (IGH) and light (IGL) chain clonotypes.

#### Neoantigen Prediction

Our protocol for annotating neoantigens requires germline and somatic variant calls and prediction of pts’ HLA-types. A similar protocol has been described before (Heindl et al., 2018), however, both for completeness and because of some differences, we summarize it again in the following.

We take our somatic variant list as shown in Table S2. Germline variants are called using Platypus (Rimmer et al., 2014) (using ucsc.hg19.fasta as reference file and default parameters). We retain only those variants that have a PASS in the FILTER column of the Platypus output, genotype quality GQ≥10, germline sample genotype different from “0/0”, germline coverage ≥10 and at least one germline variant read. If more than one alternative variant satisfies these conditions and appears in the Platypus-assigned genotype, we consider only the one with the highest allele frequency. We filter out variants found in segmental duplication regions (as found in the genomicSuperDups.bed.gz file (Bailey et al., 2002) (Bailey et al., 2001) downloaded from the UCSC Genome Browser website). Somatic mutation annotation was as described in the ‘Somatic mutation analysis’ methods. Germline variants are annotated running VEP (McLaren et al., 2016) on the cache refseq file homo_sapiens_refseq_vep_84_GRCh37 (updated 2016-02-26). Transcript sequences for both somatic and germline variants are taken from the refseq_cds.txt file (GRCh37/hg19 Feb 2009). Note that we discard approximately 1.5% of all variants because of inconsistencies between the variant annotation and the refseq_cds.txt file sequences (either the variant’s transcript ID is missing altogether or the variant annotation is not consistent with the sequence found in the refseq_cds.txt file).

For neopeptide generation, we consider the following protein–modifying germline and somatic variants: missense variants, in-frame deletions, in-frame insertions, frameshift, start lost, stop lost, stop gained and synonymous variants. Synonymous variants are only considered for their potential effect on other protein modifying mutations e.g. upstream frameshift mutations. When the genomic positions of 2 variants overlap we retain only one of the 2. For each transcript *T* carrying at least one somatic variant of the type above (transcripts with only synonymous variants are excluded for obvious reasons), we produce 2 mutated CDS files, one carrying all germline variants (germline transcript, *T*_*germ*_) and the other carrying all germline and somatic variants (tumor transcript, *T*_*tum*_). Note that, for simplicity, we consider all germline and somatic variants to be *in-phase*. We then translate the CDS sequences into amino acid sequences *S*_*germ*_ and *S*_*tum*_, respectively, and generate all associated peptides of length 8 to 11. Neopeptides associated to variants in *T* are all those generated by *S*_*tum*_ that are not generated by *S*_*germ*_. Note that since we work with CDS sequences (i.e., no UTR regions), start and stop lost variants are equivalent to missense variants that change the first and last amino acid of the protein sequence, respectively. The in-house python scripts that we use to generate neopeptides are available upon request.

We predict the pts’ HLA class I types by running the program Polysolver (Shukla et al., 2015) (version 1.0d) on normal samples (we set race=Unknown, includeFreq=1 and insertCalc=0).

Finally, we predict neopeptide likelihood of binding to HLA complexes using the program netMHCpan4.0 (Jurtz et al., 2017). For each sample, we run netMHCpan-4.0 against the corresponding neopeptide list as many times as the number of different HLA-types of the patient. We then collect the neopeptides’ HLA-specific eluted ligand likelihood percentage rank scores and the associated interaction core peptides. The interaction core peptide (*Icore* in the netMHCpan output) is the portion of the neopeptide that is predicted by netMHCpan to span the full length of the HLA binding site and thus represents the peptide most likely to be presented to T-cells. About 12.6% of all our neopeptides are predicted to have a core peptide that is shorter than the original neopeptide. For each core peptide, we store only the best (i.e., lowest) HLA percentage rank observed in the sample. Finally, we calculate the neoantigen burden in a sample as the number of core peptide high binders (%rank<0.5). Note that core peptide binders that are shorter than their corresponding neopeptides may be devoid of mutated amino acids, i.e. they may correspond to wild type peptides; these cases are excluded from the above binders’ counts.

#### Immunohistochemistry

5 μm slides were cut from FFPE blocks and triple stained as described (Gerlinger et al., 2013). 5 representative tumor areas of 0.05 mm^2^ were identified per slide and CD8^+^, FOXP3^+^ CD4^+^ cells, and CD4^+^ FOXP3^-^ T cells were quantified in each of the selected areas at 40x magnification using ImageJ software. Densities were calculated as cells/mm^2^. Immune cell scoring was performed blinded. For center and margin analysis representative areas were selected per slide, 2 areas from the invasive margin and the other 2 from the center of the tumor. Invasive margin was identified as the border region separating normal tissue from the malignant tumor cells.

#### Testing for Mismatch Repair Deficiency (dMMR) / Microsatellite Instability (MSI)

Immunohistochemistry had been performed on 18 BL biopsies to test for loss of expression of the MMR proteins MLH1, MSH2/6 and PMS2. None of these 18 biopsies showed evidence for dMMR. In addition, we considered mutation load, somatic mutation status of the MMR genes and the presence of COSMIC MSI signatures (Sig.6, Sig.15, Sig.20 and Sig.26). Mutation signature analysis was run using the R package ‘deconstructSigs’ (Rosenthal et al., 2016) (v1.8.0). Evidence of MSI was found only for C1013 based on a high mutation load and dominance of MSI mutational signatures.

#### Drug Assays

Growth Factor rescue experiments were performed in DiFi and LIM1215 colorectal cancer cell lines treated with CET (provided by Merck KG), AMG-337 and BGJ-398 (Selleckchem), FGF1, FGF2, TGFβ1, TGFβ2 and TGFβ3 (RnD Systems) and HGF and FGF10 (Peprotech) for 5 days (7 days for FGF10). Treatments were replenished with fresh media after 3 days in 7 day assays. EGFR mutant experiments were performed in LIM1215 cells. Cells were treated with CET for 5 days. DiFi and LIM1215 cells were seeded in standard media or CAF CM and treated with CET for 5 days. All experiments were performed in 6 replicates. Viability was assessed using CellTiter Blue reagent (Promega) for all assays.

#### DNA Constructs and Site Directed Mutagenesis

The Gateway Entry clones R777-E053-Hs.EGFR, R777-E015-Hs.BRAF and R777-E087-HsFGFR3 (Addgene plasmids #70337, #70299, #70371 respectively) were a gift from Dominic Esposito. Entry clone pDONR223_BRAF_p.D594H (Addgene #82816) was a gift from Jesse Boehm, Matthew Meyerson and David Root. RC201958 KRAS TrueORF gold clone was purchased from Origene and subcloned into the Gateway entry vector pENTR1A (Invitrogen) using a BamH1/EcoRV double digest. Site directed mutagenesis was performed using QuikChange Lightning (Agilent) and custom designed primers (Table S7) to generate the following mutants: EGFR D278N, FGFR3 P418L, BRAF D549N, BRAF D594F, KRAS-STOP (to remove the C-terminal tag), KRAS A18D, KRAS L19F. The full-length sequence of each clone was assessed using Sanger sequencing to confirm presence of the intended mutation and that no other mutations had been inserted. LR Gateway recombination was used to generate expression constructs using the following destination vectors: the lentiviral expression construct pLX304 (Addgene #25890, a gift from David Root), the lentiviral expression construct pLenti-CMV-Puro-DEST (Addgene #17452, a gift from Eric Campeau and Paul Kaufman) and the transient expression vector pEZY3 (Addgene #18672, a gift from Yu-Zhu Zhang). pLX304-LacZ (a gift from Steven Whittaker), pLenti-CMV-Puro-LUC (Addgene #17477, a gift from Eric Campeau and Paul Kaufman), and pEZYegfp (Addgene #18671, a gift from Yu-Zhu Zhang) were used as control vectors.

#### Transfection and Transduction

HEK293T cells were transfected with pLX304 or pLenti-CMV-Puro-DEST lentiviral constructs in combination with packaging plasmids psPAX and pMD2.G (a gift from Didier Trono, Addgene #12260 and #12259 respectively) using TransIT-LT1 (Mirus). DiFi, LIM1215 and NIH-3T3 cells were transduced with the resultant viral supernatants in the presence of Polybrene (8 μg/mL), and selected with 5 μg/mL Blasticidin (pLX304) or 5 μg/mL Puromycin (pLenti). DiFi and LIM1215 cells were transiently transfected with pEZY constructs using Lipofectamine2000 (Invitrogen) according to the manufacturer’s protocol and selected with 0.5 μg/mL Neomycin.

siRNA mediated knockdown of *NF1* in DiFi and LIM1215 cells was performed using Dharmacon siGenome pool and Lipofectamine RNAiMAX (Invitrogen) according to the manufacturer’s recommended protocol.

#### CRISPR Mediated *NF1* Inactivation

LIM1215 cells were transduced with Cas9 viral particles (a gift from Feifei Song, Stephen Pettitt and Chris Lord, derived from lentiCas9-Blast (Addgene # 52962, a gift from Feng Zhang)) in the presence of Polybrene (8 μg/mL) and selected with 5 μg/mL Blasticidin to create constitutively expressing Cas9 lines, confirmed by Western blotting using Cas9 (7A9-3A3) antibody (Cell Signalling Technologies #14697). To produce lentiviral guide RNAs targeting *NF1*, HEK293T cells were transfected with pLentiguide-NF1#1 and pLentiguide-NF1#2 (a gift from Stephen Pettitt and Chris Lord, customized from pLentiguide-Puro (Addgene #52963, a gift from Feng Zhang)) in combination with packaging plasmids psPAX and pMD2.G. LIM1215-Cas9 cells were transduced with the resultant viral gRNA supernatants in the presence of Polybrene (8 μg/mL).

#### Western Blotting

Total cell lysates were prepared using NP-40 buffer supplemented with protease and phosphatase inhibitors (Sigma). Samples were resolved by electrophoresis on SDS-PAGE gels for Western blotting. Primary antibodies used were p-ERK (Cell Signalling Technologies #9101), ERK (Cell Signalling Technologies #9102), p-EGFR (Cell Signalling Technologies #2236), EGFR (Cell Signalling Technologies #2232) and NF1 (Cell Signalling Technologies #14623). HRP-conjugated anti-beta Tubulin antibody (Abcam #ab21058) was used as a loading control. Bands were detected using HRP-labelled secondary antibodies and ECL Prime (GE Healthcare), followed by visualisation on an Azure Biosystems C300 detection system.

### Quantification and Statistical Analysis

Statistical analyses were performed using R (v3.4.0) and STATA13. The Fisher’s exact test was used to examine association of categorical variables in 2x2 contingency tables. The Student’s t-test was applied to examine means of continuous data (e.g. normalized RNA-Sequencing counts, cytolytic activity scores, median expression values of the T cell associated inflammation signature, immunohistochemical immune cell densities and MCP-counter (Becht et al., 2016b) fibroblast infiltrate scores from non-paired sample groups). The paired Student’s t-test was applied to these datasets when comparing paired (BL and PD) data. p values ≤0.05 were considered statistically significant. The Kaplan-Meier method was used to estimate OS and PFS probability. The Mann-Whitney statistical test was applied to compare ssGSEA rank scores of 28 immune cell populations followed by False Discovery Rate correction and a q value ≤ 0.1 was considered statistically significant.

### Data and Software Availabilty

#### Sequencing Data Deposition in Public Repositories

The accession number for the DNA and RNA sequencing data reported in this paper is (EGA: EGAS00001003367). Datasets are password protected and will be shared with researchers subject to signing a data sharing agreement.

### Additional Resources

Prospect-C trial information on ClinicalTrials.gov identifier: clinicaltrials.gov/ct2/show/NCT02994888.

## Supplemental Information

Document S1. Figures S1–S5 and Tables S6 and S7

Table S1. Patient Characteristics and Sample Data, Related to Figure 1

Table S2. Somatic Mutations Identified for Each Patient, Related to Figure 1

Table S3. Data Supporting the Classification of Recurrently Mutated Genes in Progression Biopsies as Likely Passengers, Related to Table 1

Table S4. Amplicon Sequencing of Tissue Biopsies, Related to Figure 5

Table S5. Targeted Sequencing of ctDNA, Related to Figure 5

Data S1. Genome-wide DNA Copy-Number Profiles of BL and PD Biopsies, Related to Figure 2

Data S2. Mutation Clonality Assessment, Related to Figure 2

Data S3. Copy-Number Profile Comparison of Biopsy and ctDNA Samples, Related to Figure 5

Document S2. Article plus Supplemental Information
