# Supplementary material for: Genomic and Transcriptomic Determinants of Therapy Resistance and Immune Landscape Evolution during Anti-EGFR Treatment in Colorectal Cancer
Source: Cancer Cell. 2019 Jul 8;36(1):35–50.e9. doi: 10.1016/j.ccell.2019.05.013 (PMC6617392; doi:10.1016/j.ccell.2019.05.013)
Supplement: Document S1. Figures S1–S5 and Tables S6 and S7 [file mmc1.pdf]

Cancer Cell, Volume *36*

Supplemental Information

Genomic and Transcriptomic Determinants of Therapy Resistance and Immune Landscape Evolution during Anti-EGFR Treatment in Colorectal Cancer

Andrew Woolston, Khurum Khan, Georgia Spain, Louise J. Barber, Beatrice Griffiths, Reyes Gonzalez-Exposito, Lisa Hornsteiner, Marco Punta, Yatish Patil, Alice Newey, Sonia Mansukhani, Matthew N. Davies, Andrew Furness, Francesco Sclafani, Clare Peckitt, Mirta Jiménez, Kyriakos Kouvelakis, Romana Ranftl, Ruwaida Begum, Isma Rana, Janet Thomas, Annette Bryant, Sergio Quezada, Andrew Wotherspoon, Nasir Khan, Nikolaos Fotiadis, Teresa Marafioti, Thomas Powles, Stefano Lise, Fernando Calvo, Sebastian Guettler, Katharina von Loga, Sheela Rao, David Watkins, Naureen Starling, Ian Chau, Anguraj Sadanandam, David Cunningham, and Marco Gerlinger

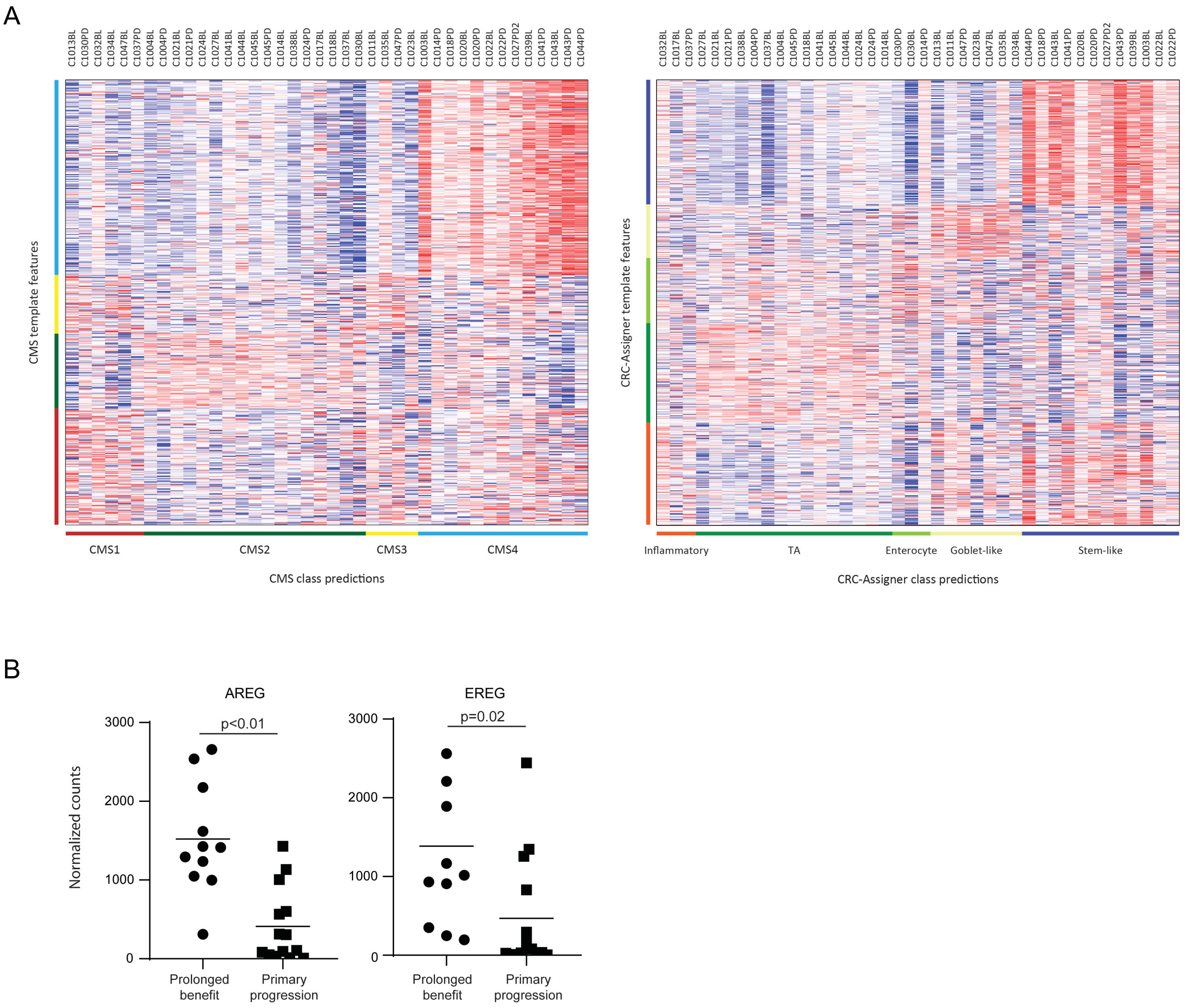

Figure S1: Transcriptomic CRC-subtype assignments. Related to Figure 4. **(A)** Heatmap of transcriptomic CRC subtype assignments. **(B)** Expression of EGFR ligands AREG and EREG in BL biopsies. Line indicates mean. Student's t-test was used to determine significance.

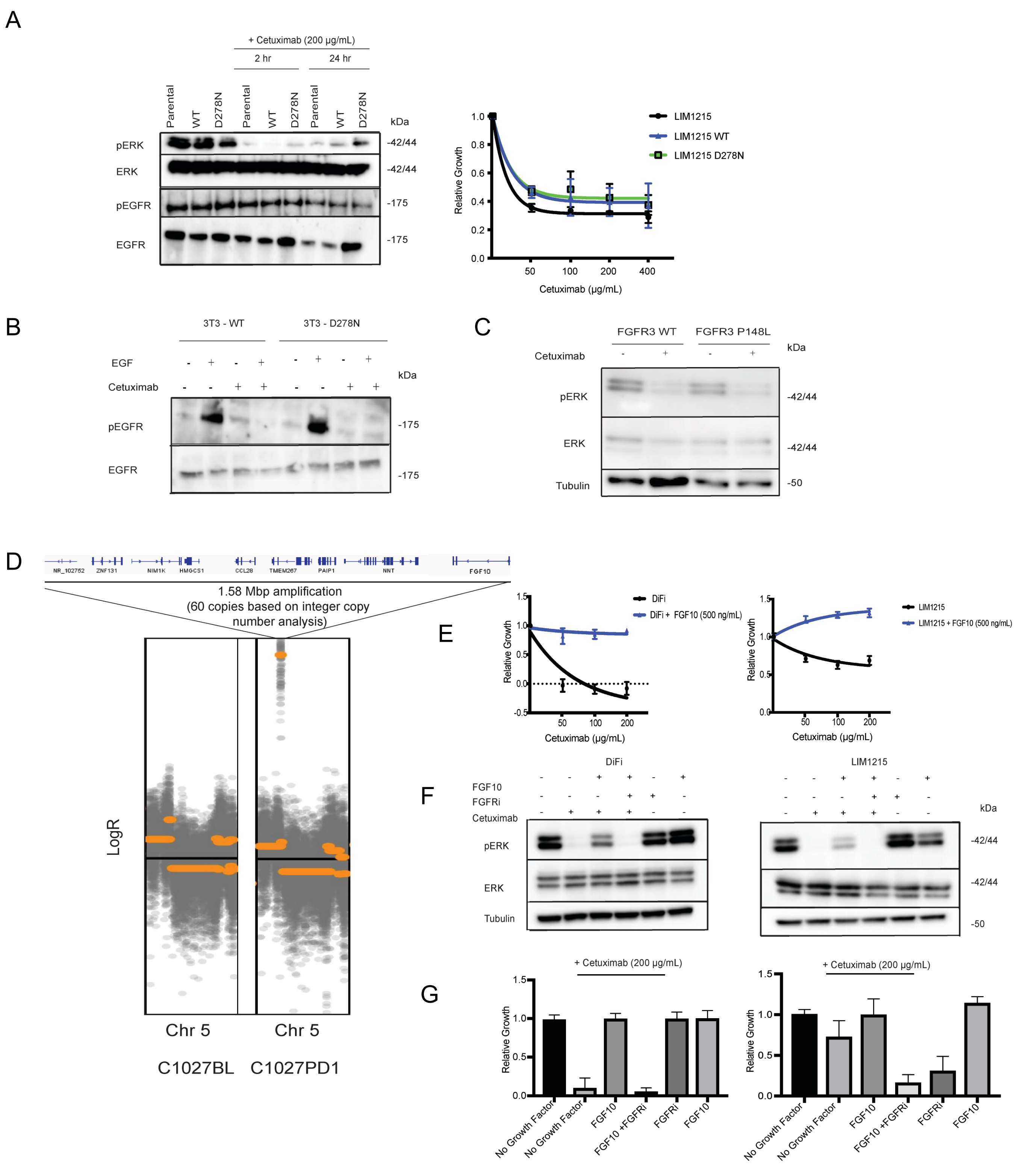

Figure S2: Functional analyses of acquired genetic alternations. Related to Figure 5. **(A)** Western blot and growth analysis (5 day treatment) of EGFR D278N mutation in LIM1215 cells treated with cetuximab. Error bars represent ±SD. **(B)** Western blot analysis of pEGFR in NIH-3T3 cells with D278N mutation. Cells were stimulated with EGF (100 ng/mL) for 5 min before 2 hr Cetuximab treatment. **(C)** Western blot analysis of pERK expression in FGFR3 WT- and P418L following 2 hr Cetuximab treatment (200 µg/mL) in DiFi cells. **(D)** High level amplification encompassing FGF10 in C1027PD1. **(E)** Growth analysis of DiFi and LIM1215 cell lines treated with recombinant human FGF10 (500 ng/mL) and cetuximab for 7 days. Error bars represent ±SD. **(F)** Western blots showing pERK expression in DiFi and LIM1215 treated with 200 µg/mL cetuximab, FGF10 and 1mM FGFR-inhibitor (FGFRi) (BGJ-398) for 2 hr alone or in combination. **(G)** Growth analysis in DiFi and LIM1215 treated with 200 µg/mL Cetuximab, FGF10 and 1mM FGFRi (BGJ-398) for 5 days alone or in combination. All error bars represent ±SD of 6 replicates.

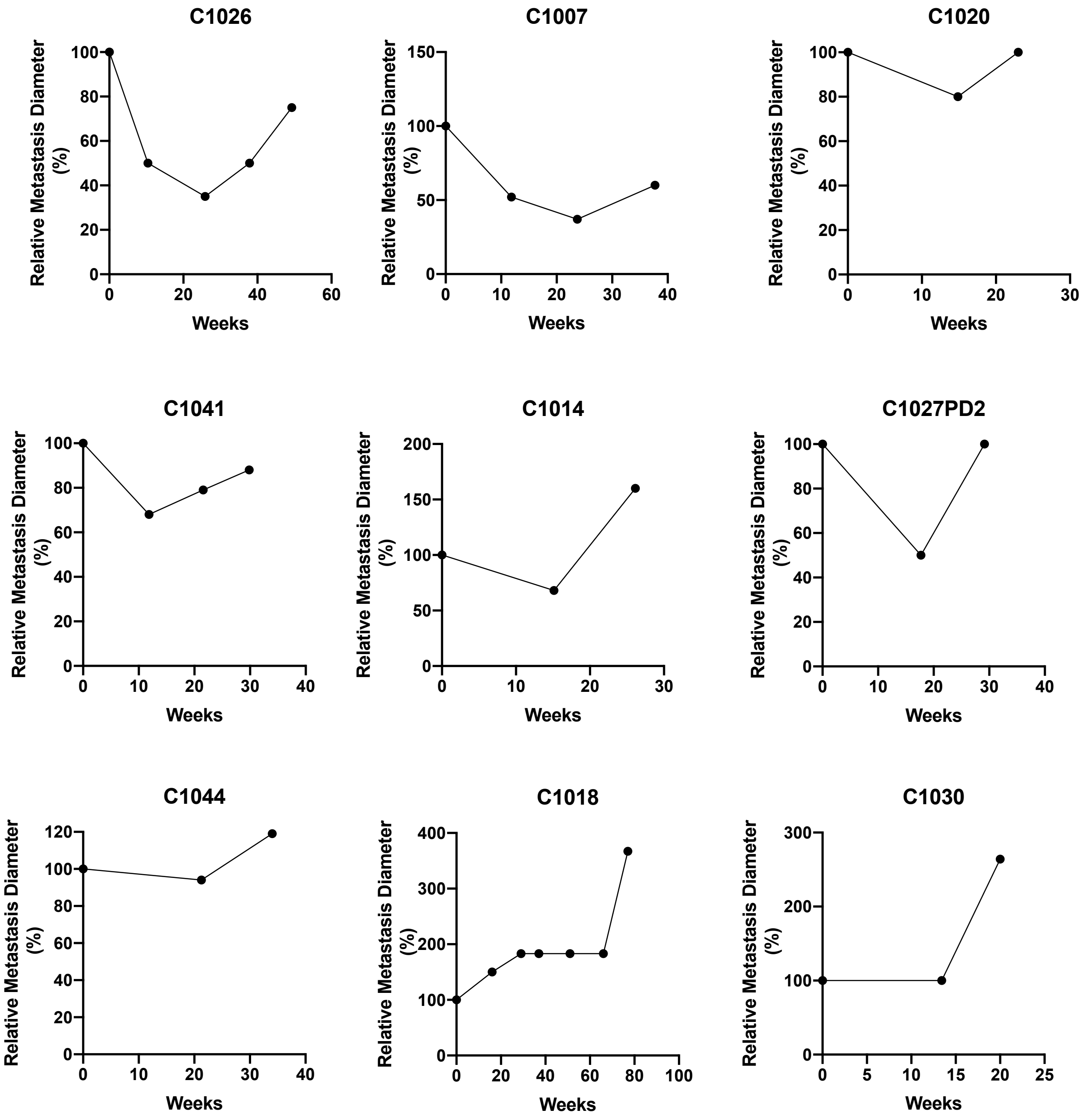

Figure S3: Radiological responses of metastases in patients who achieved prolonged benefit but had no genetic resistance driver when biopsies at PD. Related to Figure 5. Changes in diameter are relative to the BL scan.

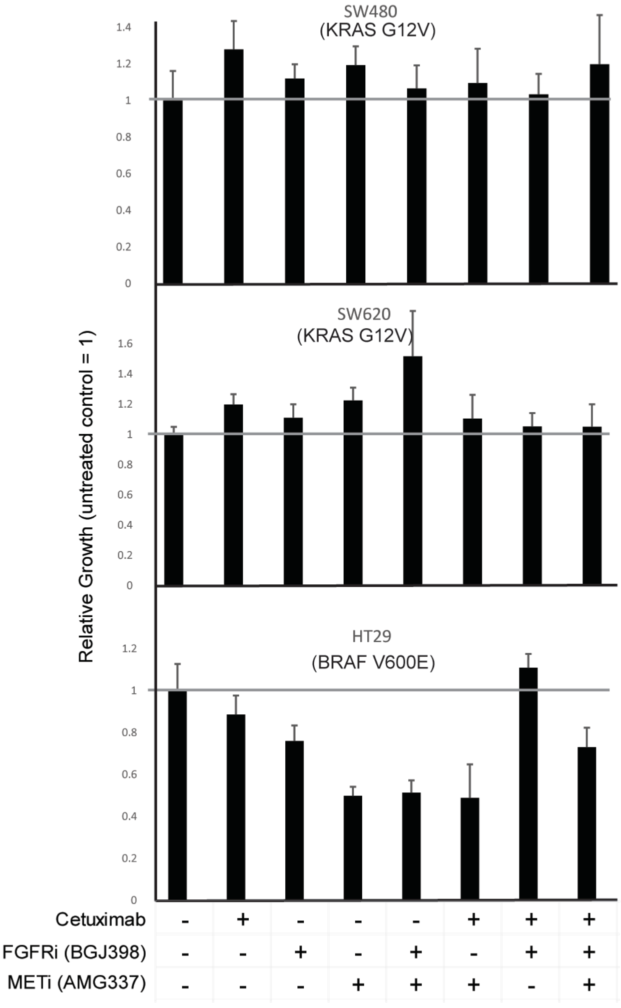

Figure S4: **Growth analysis of KRAS and BRAF mutant cell lines treated with cetuximab**. Related to Figure 6. Cell lines were treated with Cetuximab (200 µg/mL), MET- (100 nM) and pan-FGFR-inhibitors (1mM) alone or in combination for 5 days. Error bars indicate ± SD.

Table S6: RNA sequencing gene expression counts (TGFβ1-3, HGF and FGF1-2) for C1020. Related to Figure 6.

	BL	PD	Fold Change (PD/BL)
TGFB1	909	1166	1.3
TGFB2	230	1015	4.4
TGFB3	148	617	4.2
HGF	181	483	2.7
FGF1	76	64	0.8
FGF2	44	69	1.6

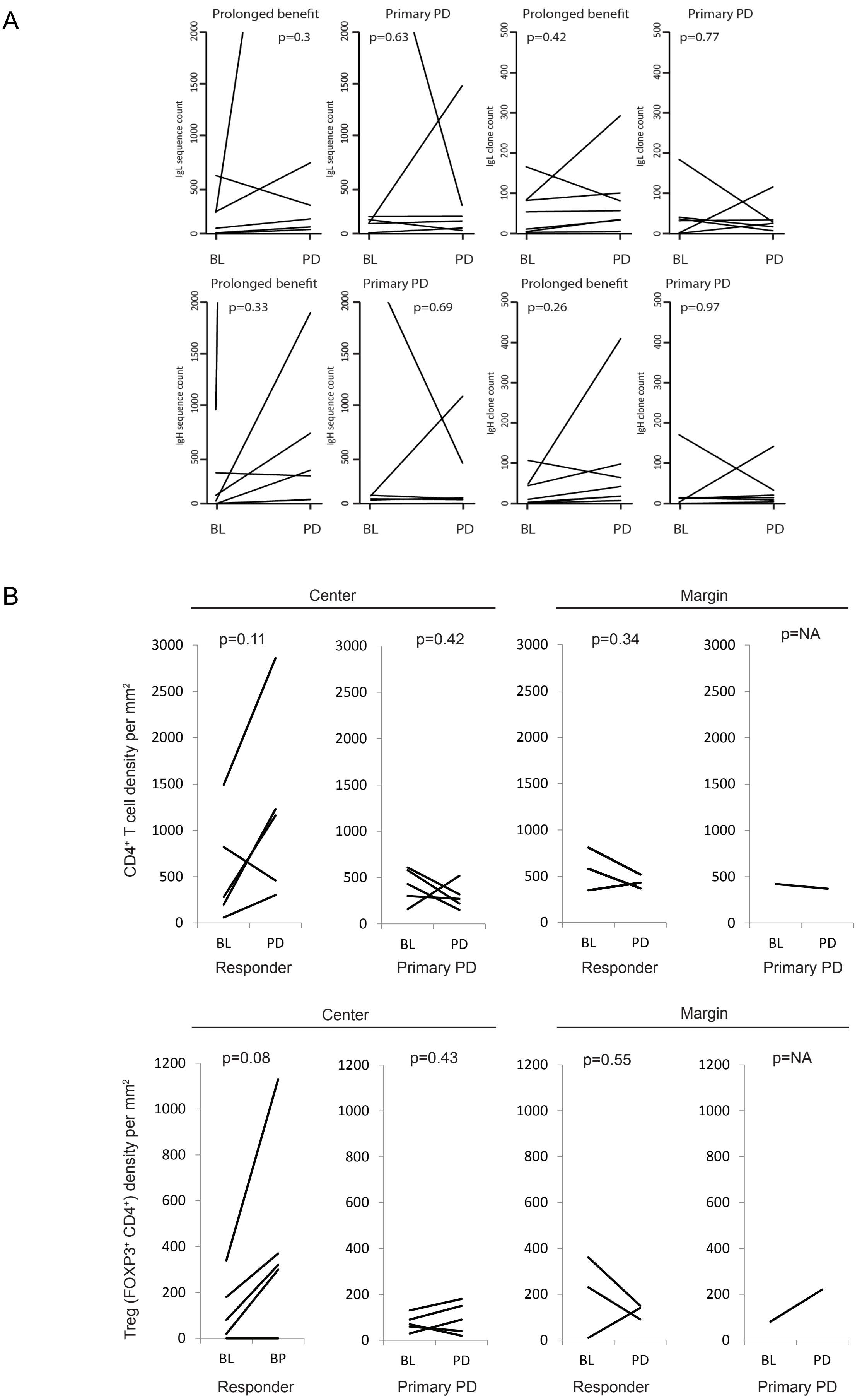

Figure S5: Assessment of immune cell infiltrates. Related to Figure 7. **(A)** B cell receptor light (IgL) and heavy chain (IgH) sequence and clonotype counts. The paired Student's t-test was used to assess statistical significance. **(B)** Density of $CD4^+$ and Tregs ($FOXP3^+CD4^+$) in the tumor center and at the margin.

Table S7: Custom designed primer sequences for QuikChange mutagenesis. Related to STAR Methods.

**Variant**	**QuikChange primer ID**	**Primer sequence**
EGFR-D278N	EGFR_QCh_D278N_F	cctcggggttcacattcatctggtacgtggt
	EGFR_QCh_D278N_R	accacgtaccagatgaatgtgaaccccgagg
FGFR3-P148L	FGFR3_QCh_P418L_F	cctgtcgcttgagcaggaagcgggagatc
	FGFR3_QCh_P418L_R	gatctcccgcttcctgctcaagcgacagg
BRAF-D594N	BRAF_QCh_D594N_F	tcactgtagctagaccaaaattacctatttttactgtgaggtc
	BRAF_QCh_D594N_R	gacctcacagtaaaaataggtaattttggtctagctacagtga
BRAF-D594F	BRAF_QCh_D594F_F	gatttcactgtagctagaccaaaaaaacctatttttactgtgaggtcttca
	BRAF_QCh_D594F_R	tgaagacctcacagtaaaaataggtttttttggtctagctacagtgaaatc
KRAS-STOP	KRAS_MUTSTOP_F	gagcggccgcgtacgctacataattacacactttgtctttg
	KRAS_MUTSTOP_R	caaagacaaagtgtgtaattatgtagcgtacgcggccgctc
KRAS-A18D	KRAS_QCh_A18D_F	tagctgtatcgtcaagtcactcttgcctacgcc
	KRAS_QCh_A18D_R	ggcgtaggcaagagtgacttgacgatacagcta
KRAS-L19F	KRAS_QCh_L19F_F	attagctgtatcgtaaaggcactcttgcctacgc
	KRAS_QCh_L19F_R	gcgtaggcaagagtgcctttacgatacagctaat
