# Supplementary material for: Genomic and Transcriptomic Determinants of Therapy Resistance and Immune Landscape Evolution during Anti-EGFR Treatment in Colorectal Cancer
Source: Cancer Cell. 2019 Jul 8;36(1):35–50.e9. doi: 10.1016/j.ccell.2019.05.013 (PMC6617392; doi:10.1016/j.ccell.2019.05.013)

## Data S1: Genome wide DNA copy number profiles of BL and PD biopsies. Related to Figure 2. Integer copy number profiles are shown for all samples where tumor content was sufficient for analysis with the Sequenza algorithm and logR data is shown for five biopsies where tumor content was insufficient. Amplifications acquired at PD and harboring genes encoding for RTKs or RAS/RAF pathway members are labelled with the contained member of the RTK-RAS/RAF pathway. Amplifications acquired at PD but not containing RTKs or RAS/RAF pathway members are labelled with gene symbol or with the genomic coordinates of the amplified segment.


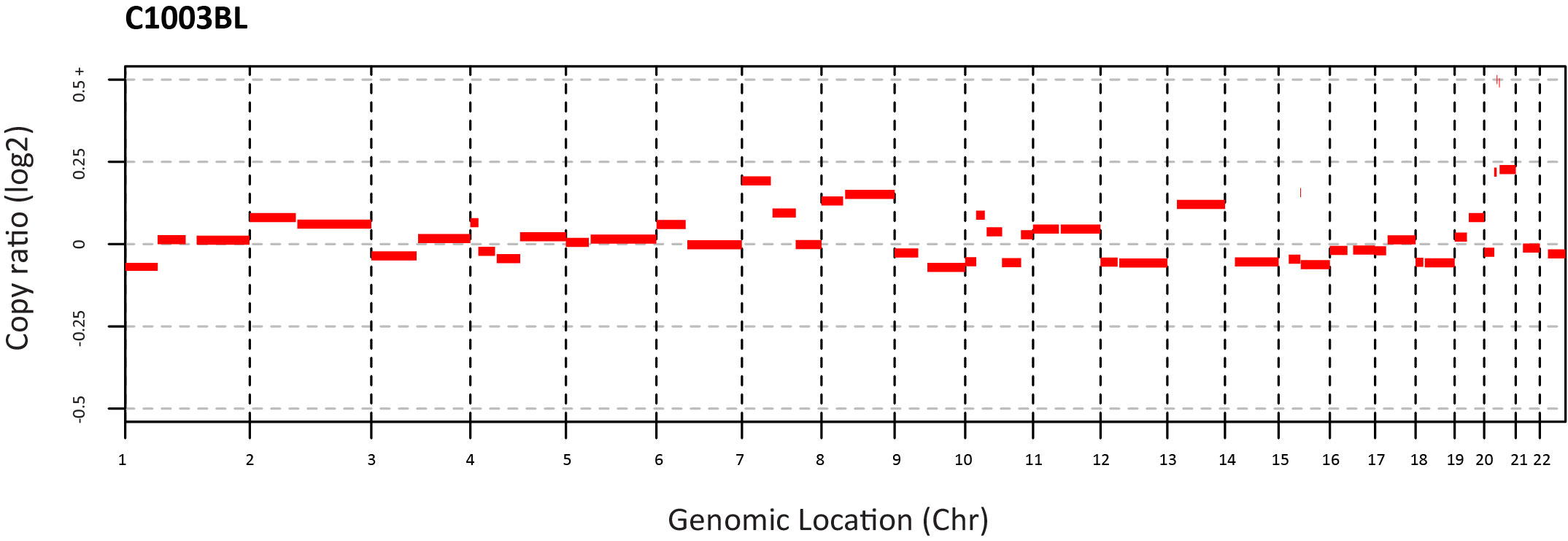


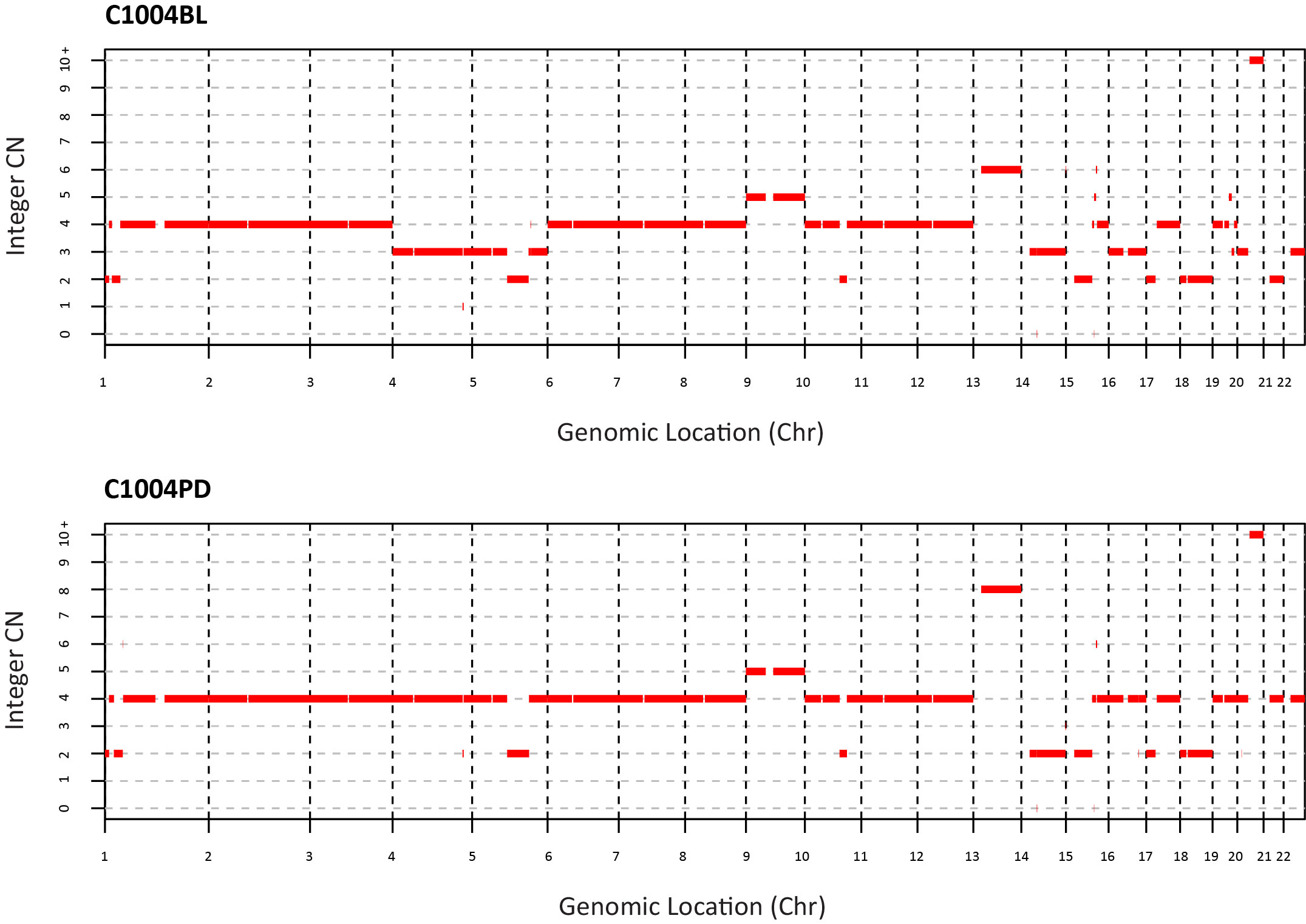


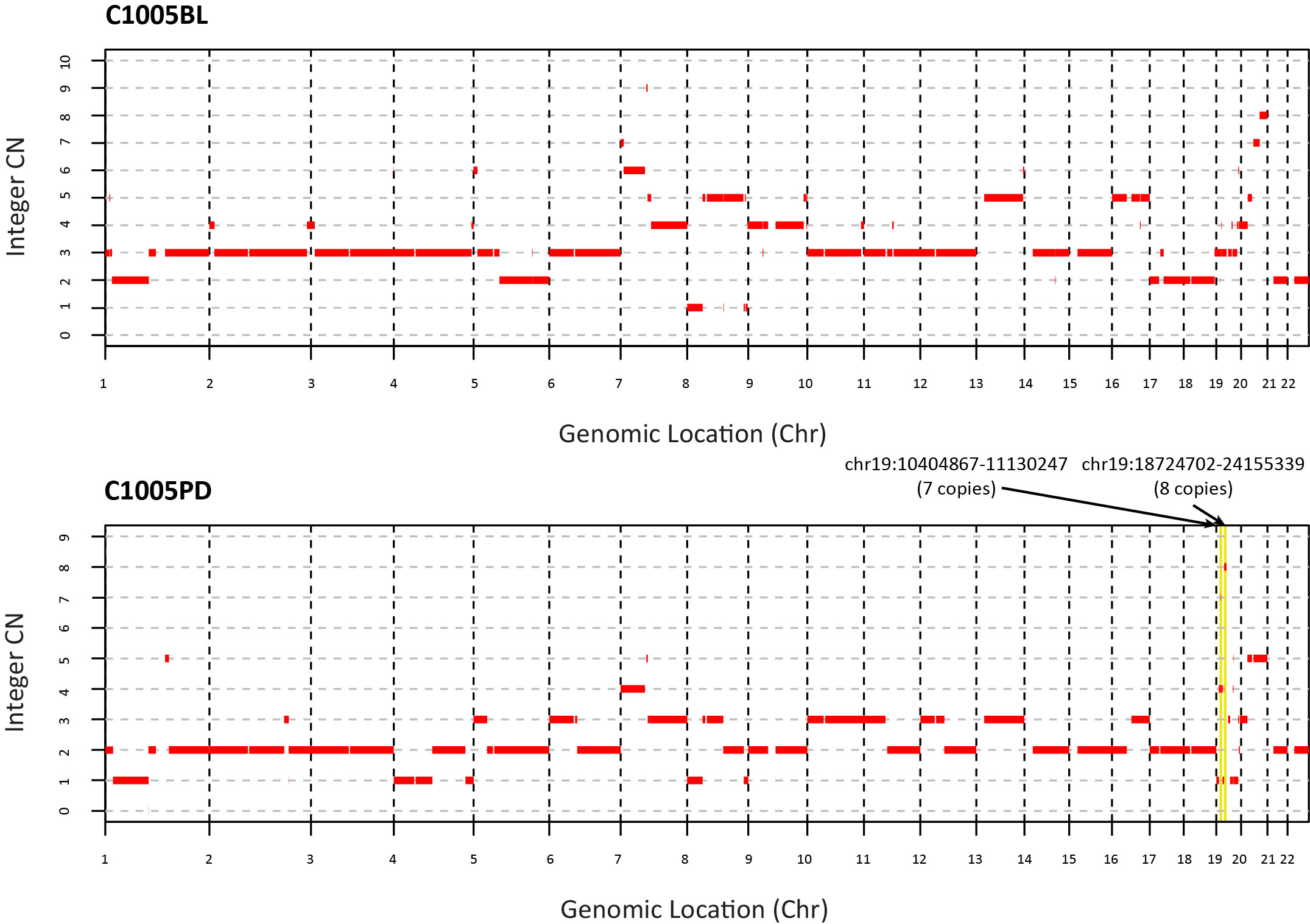


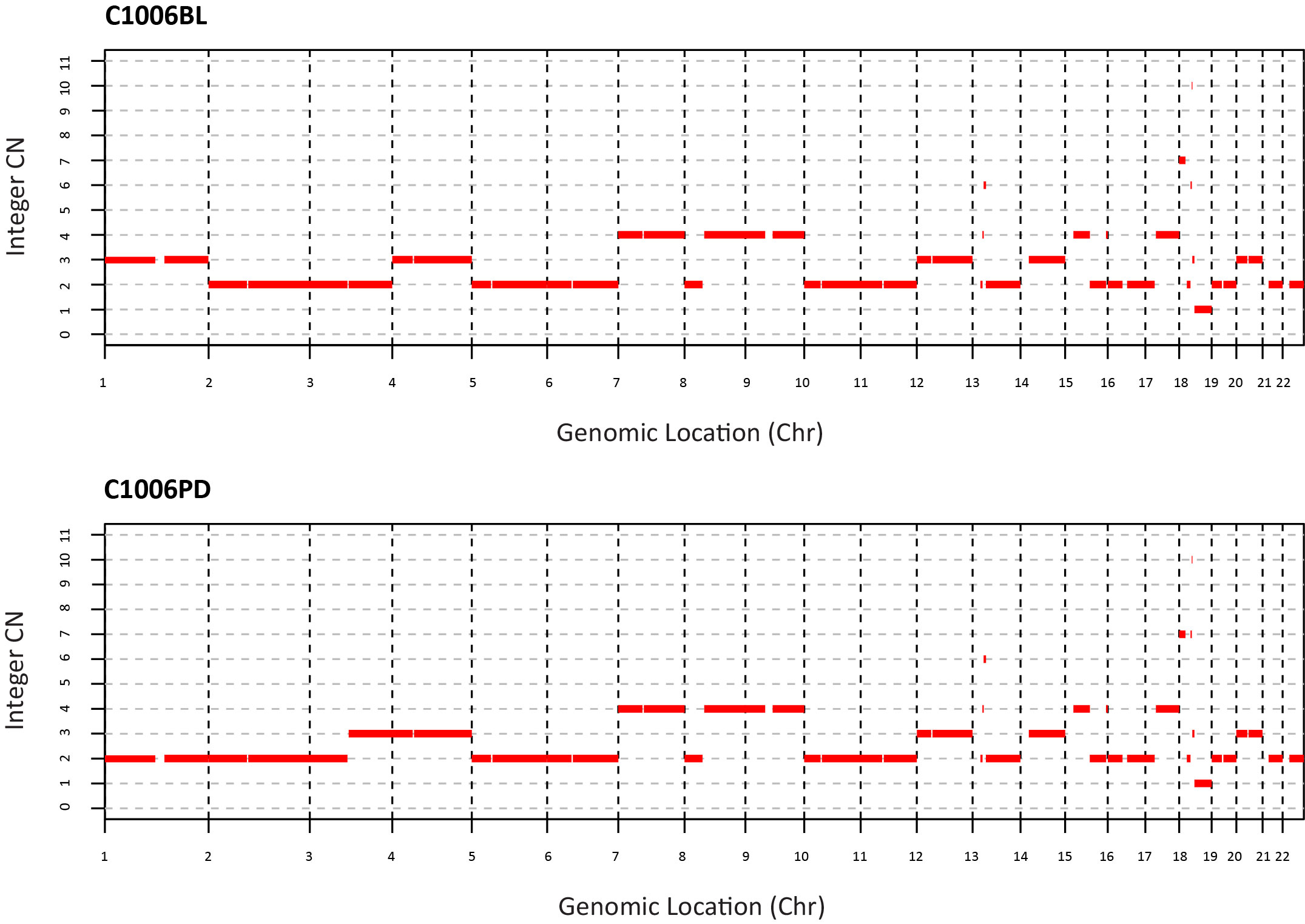


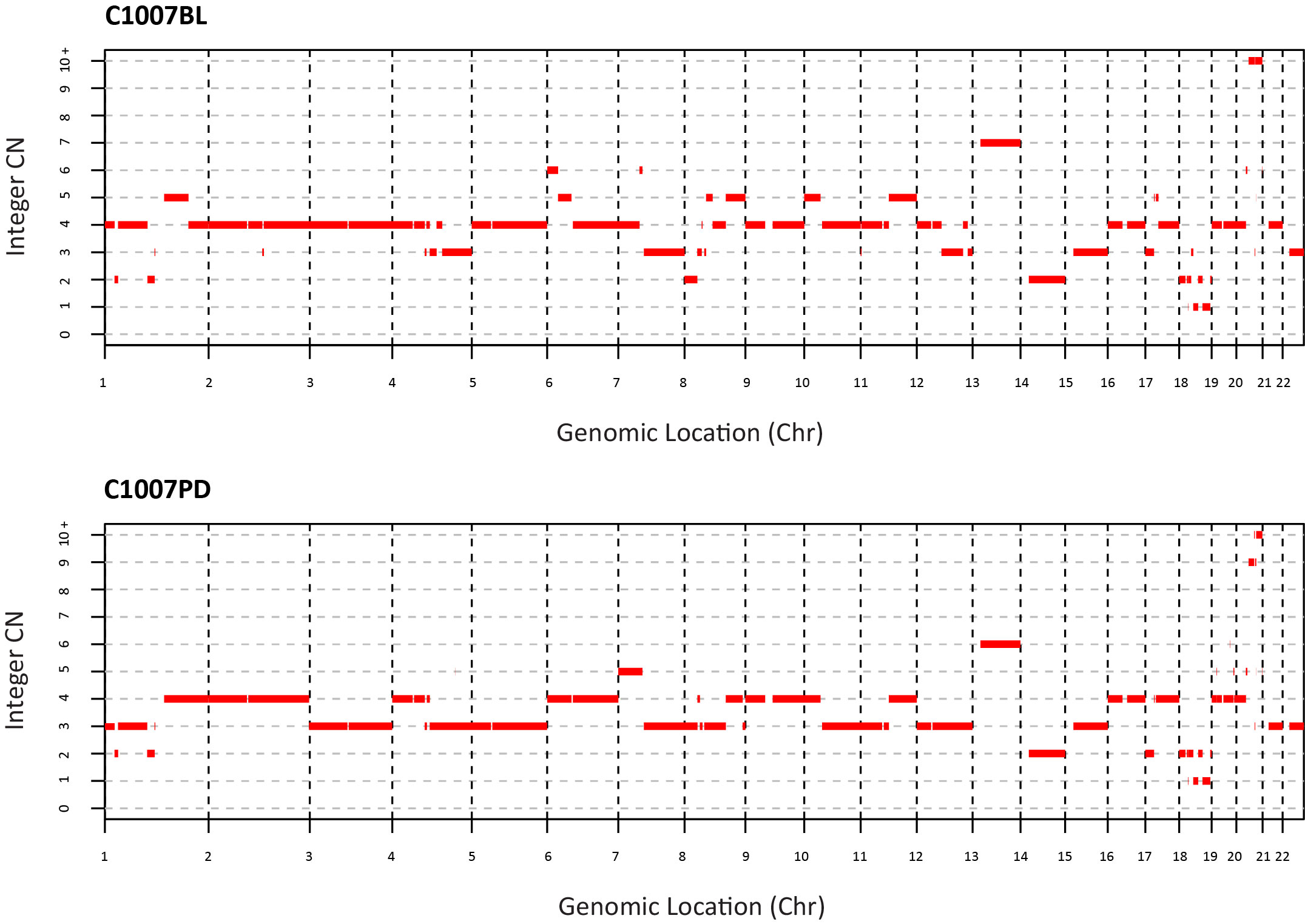


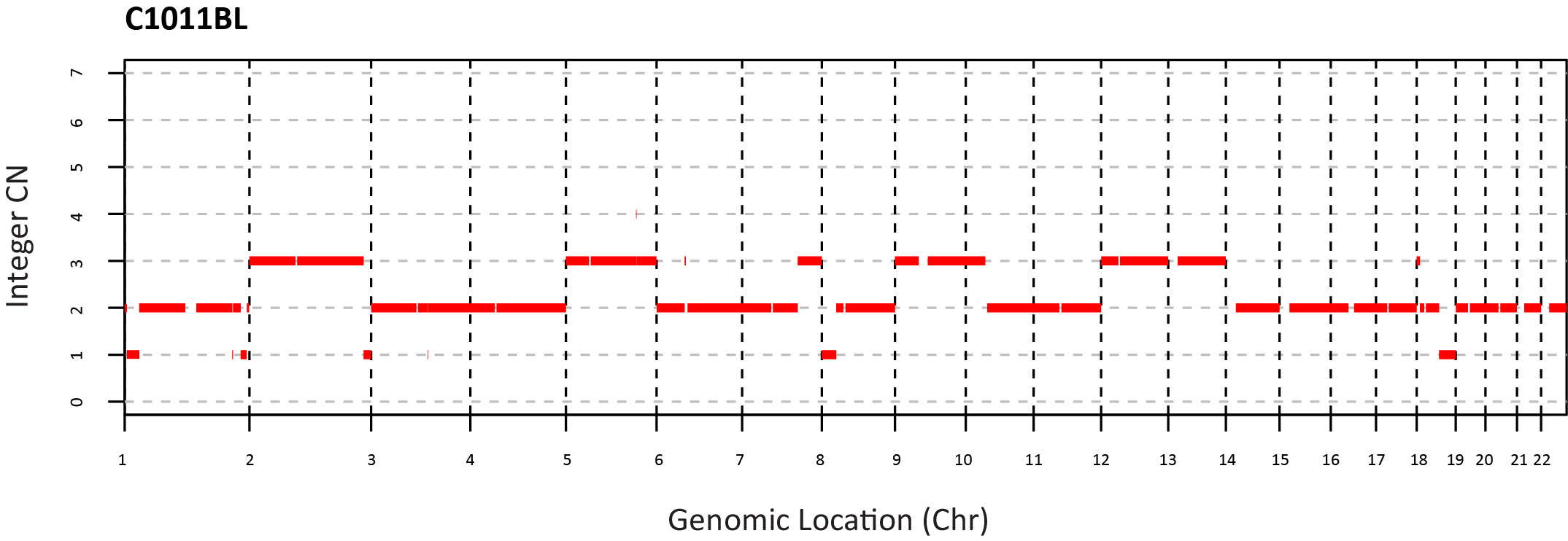


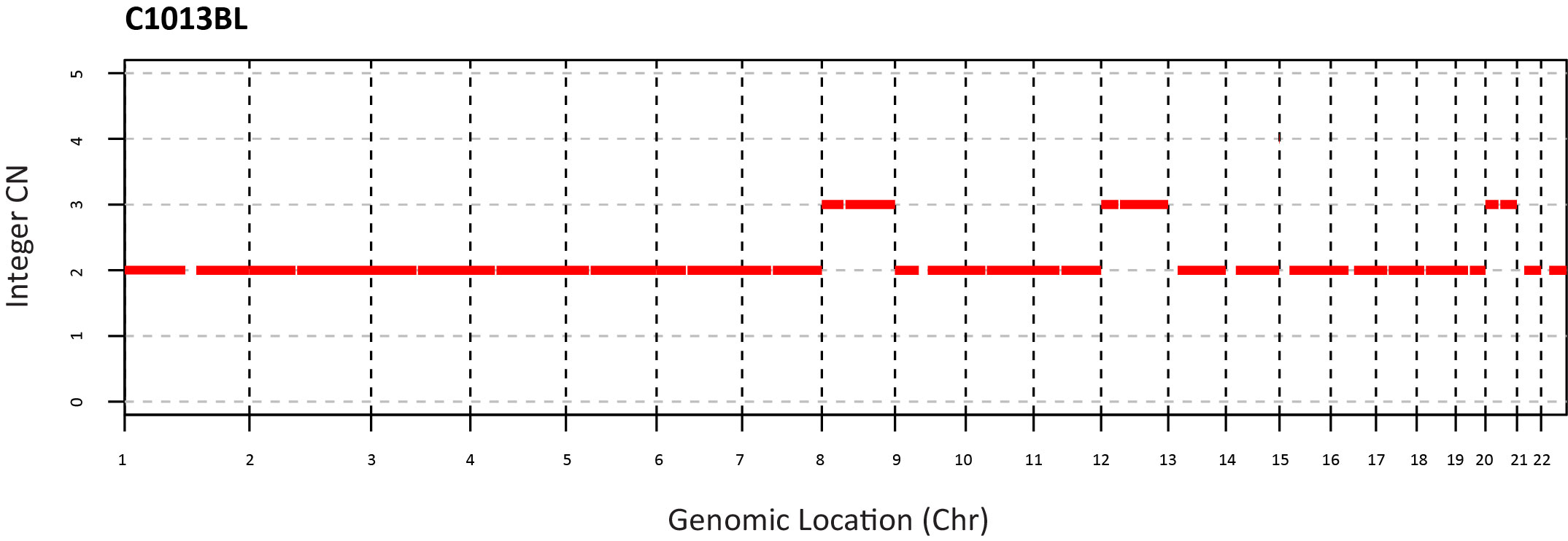


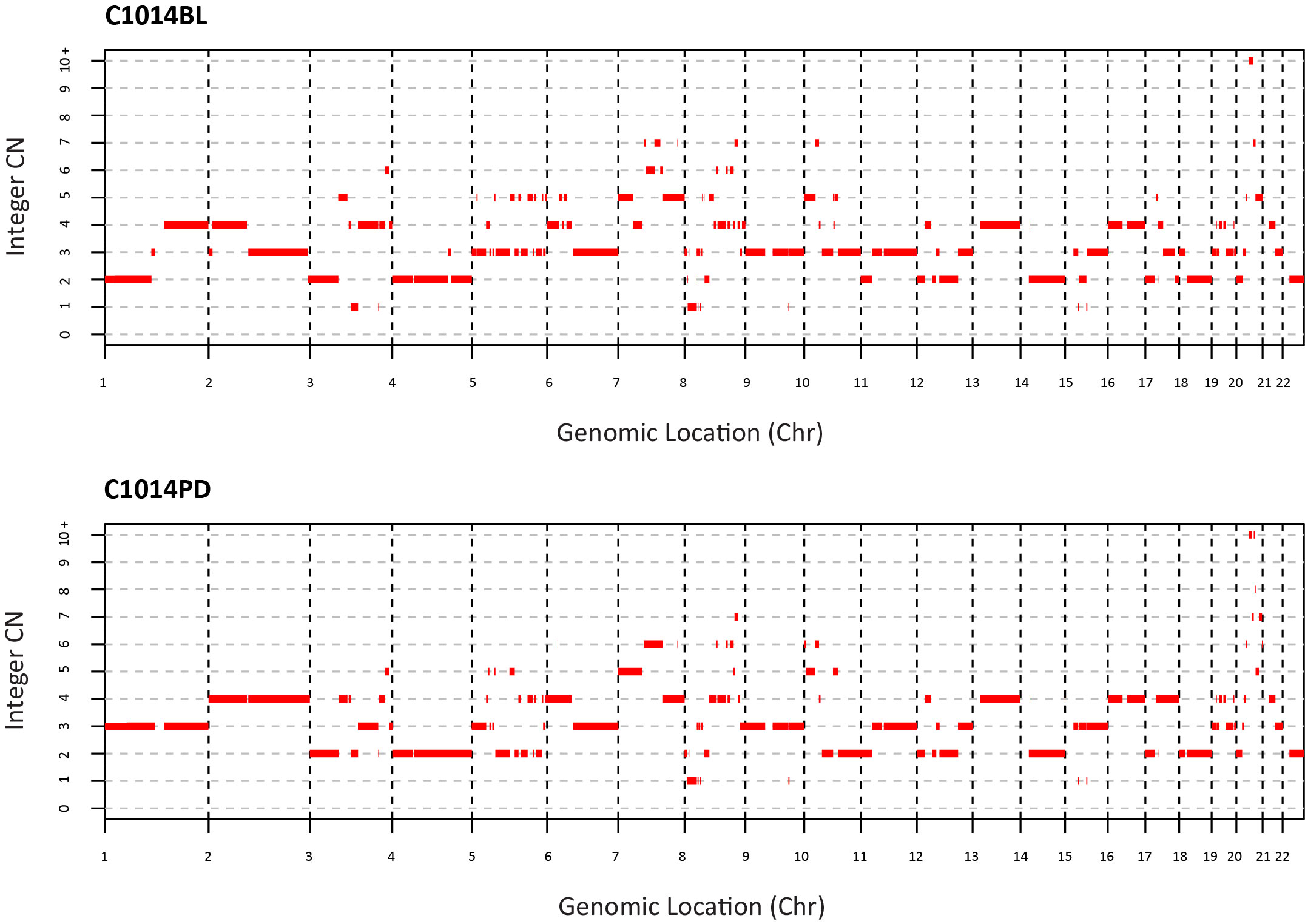


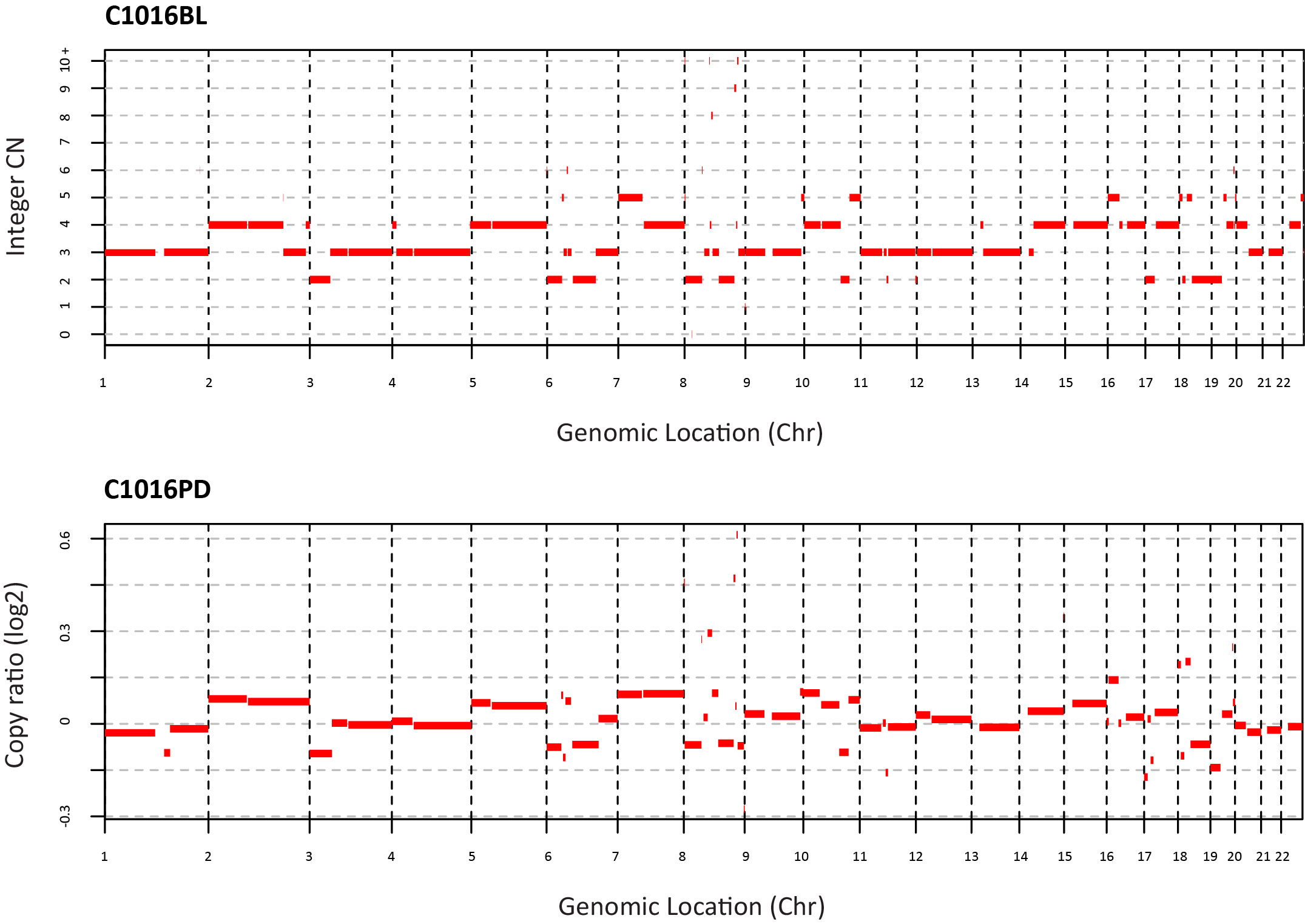


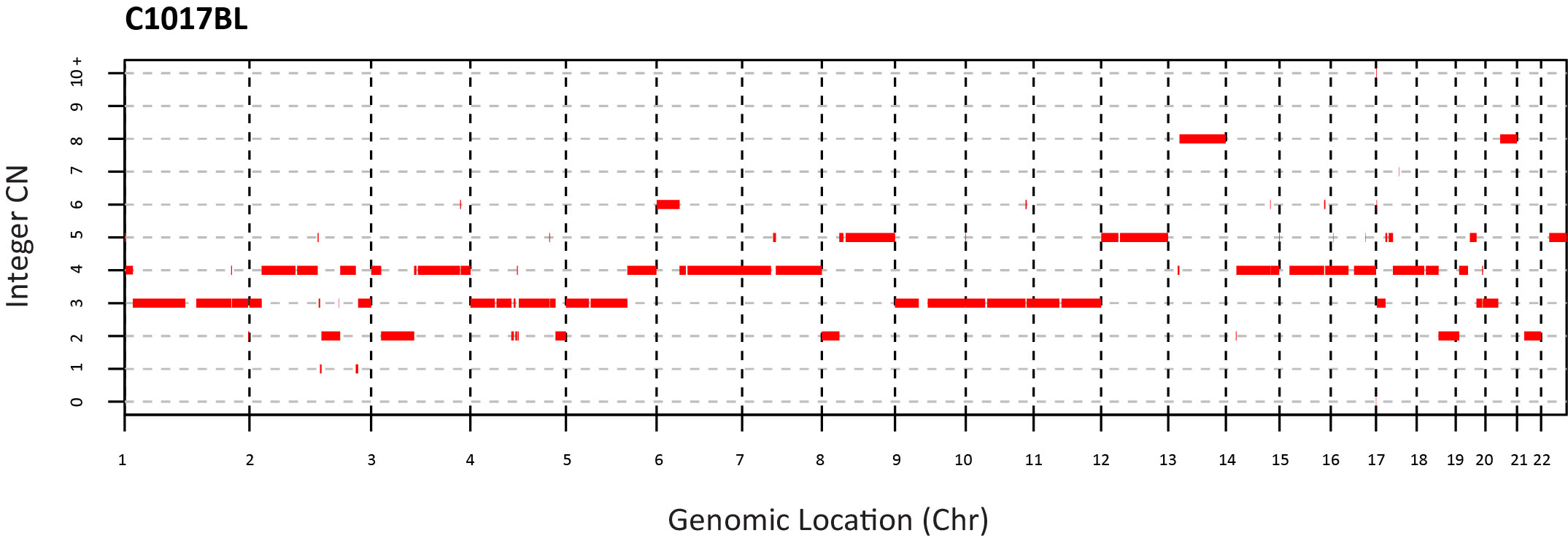


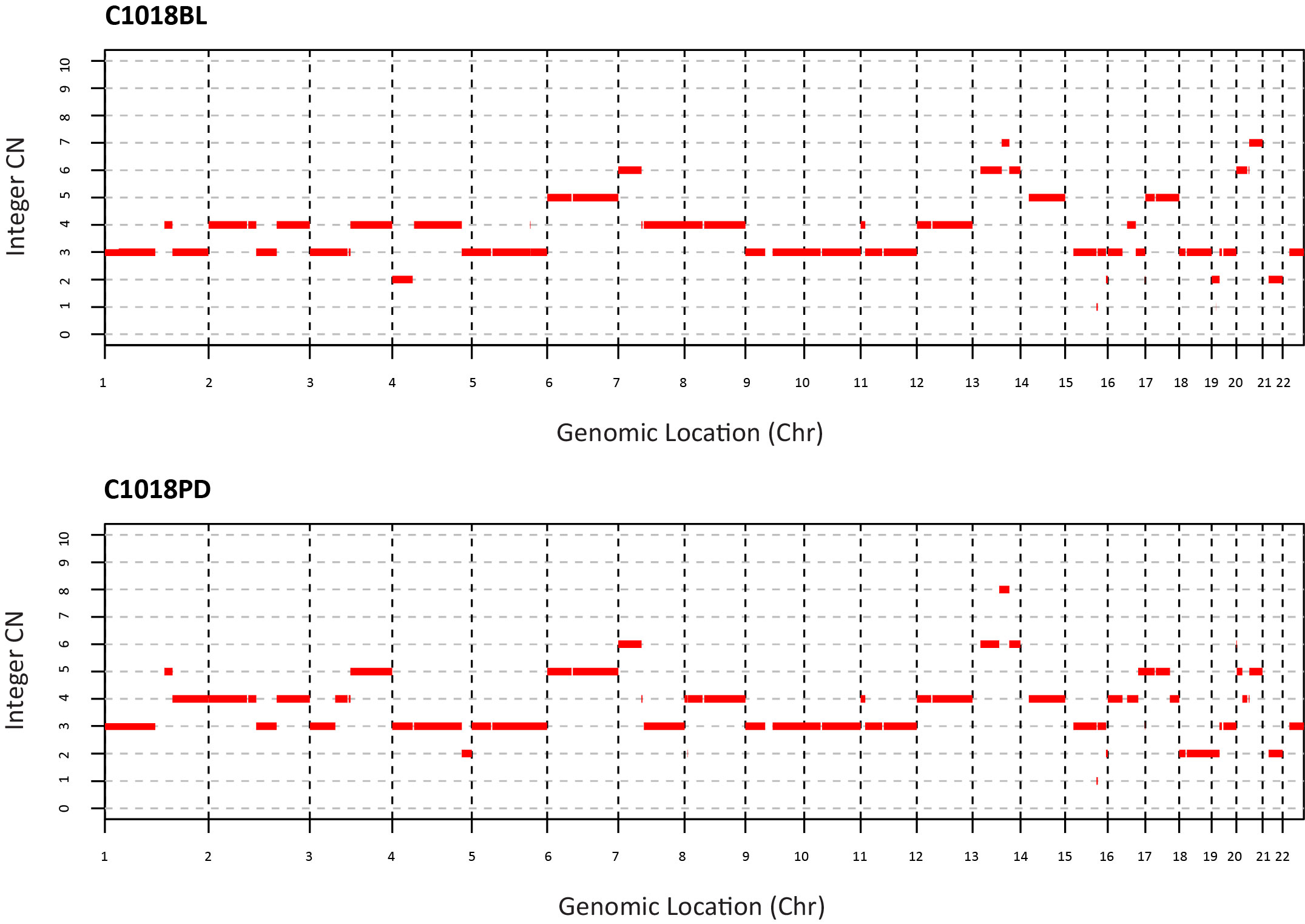


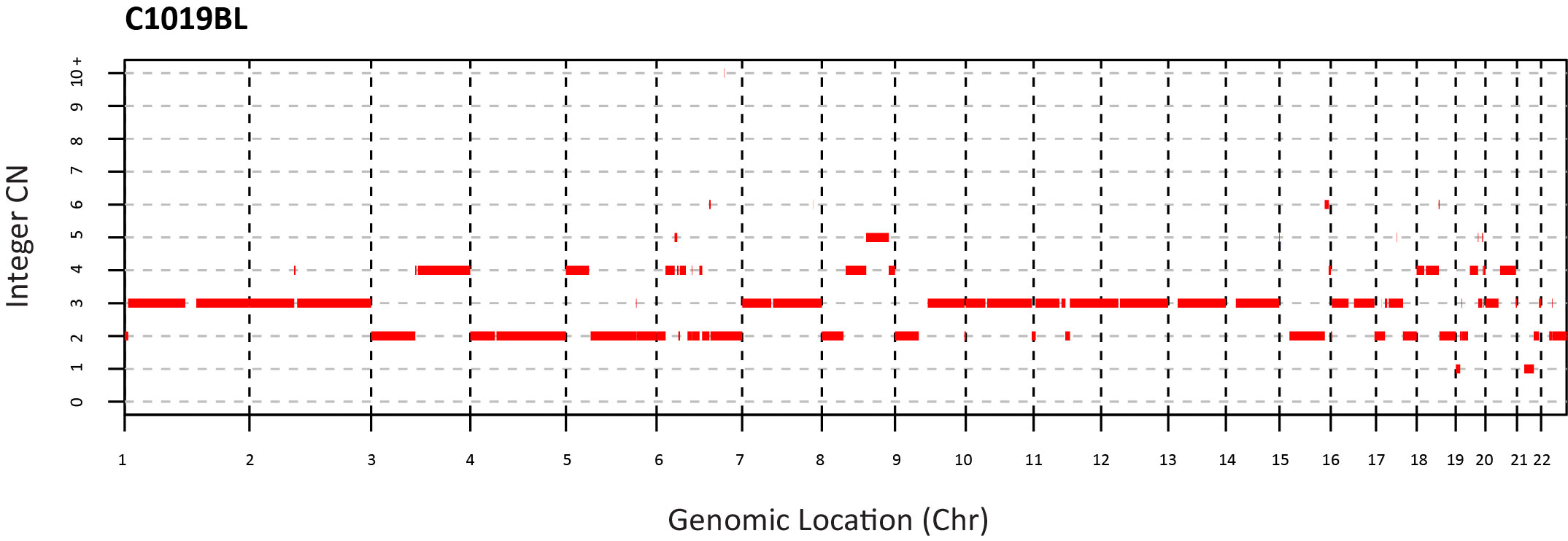


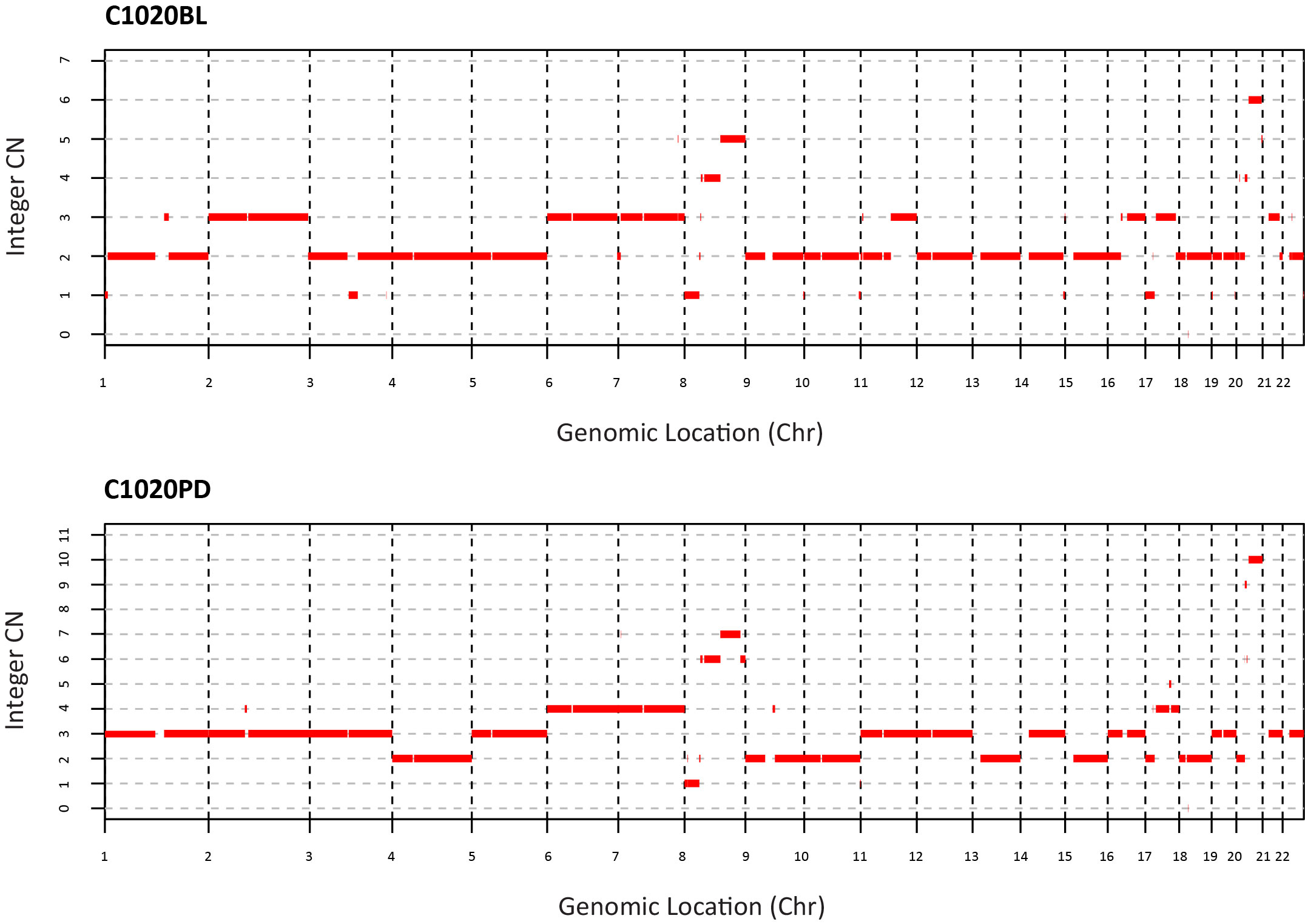


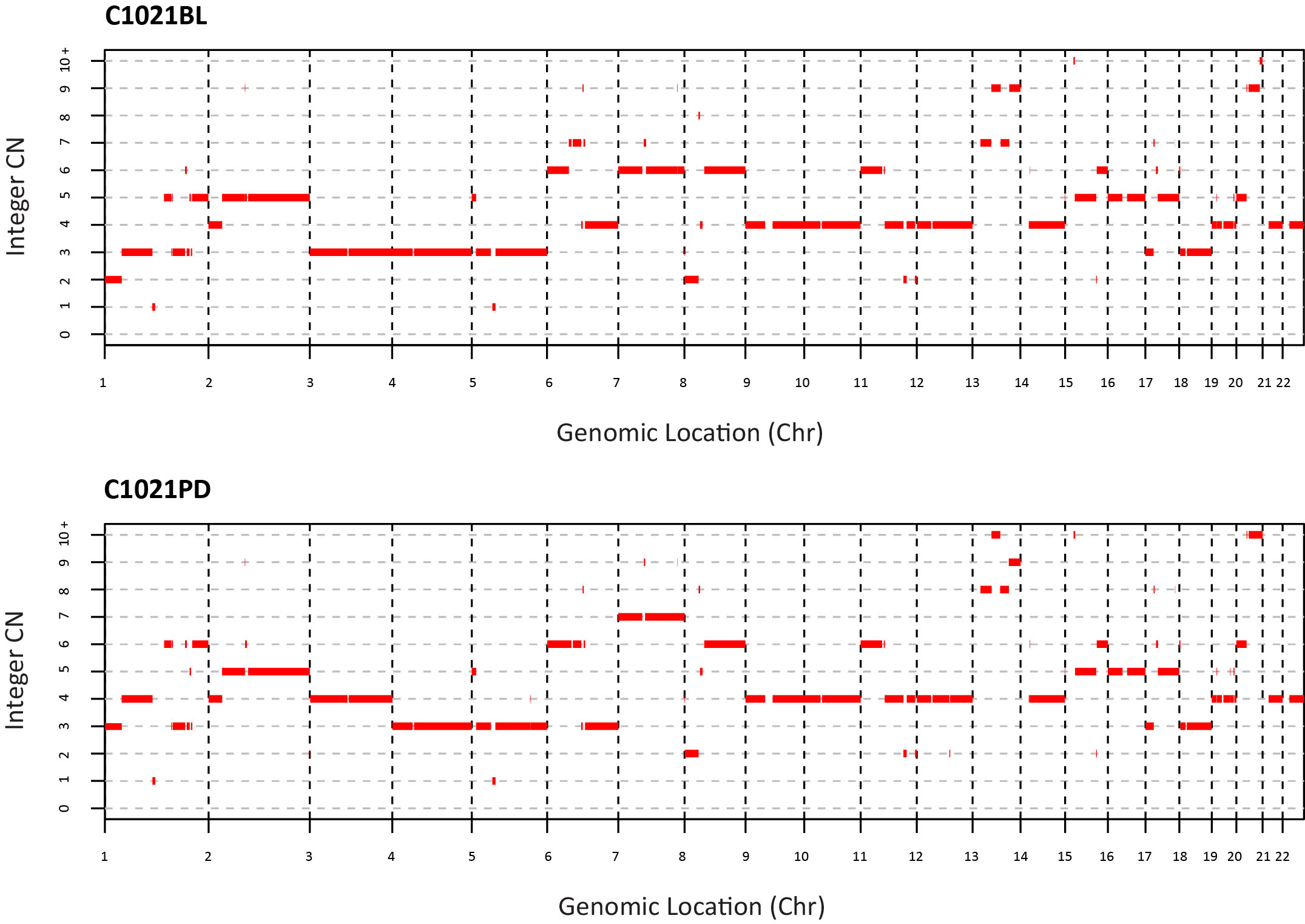


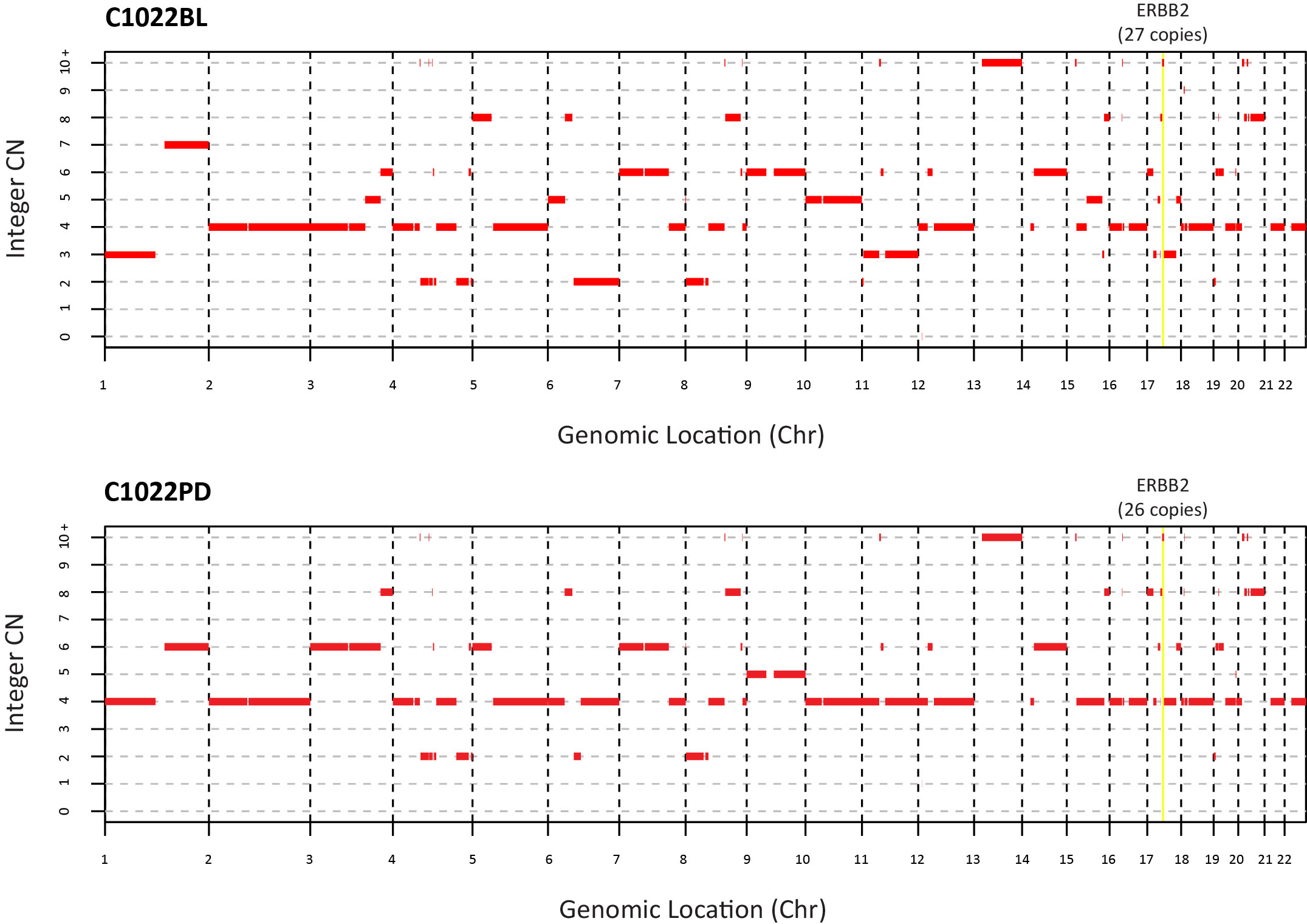


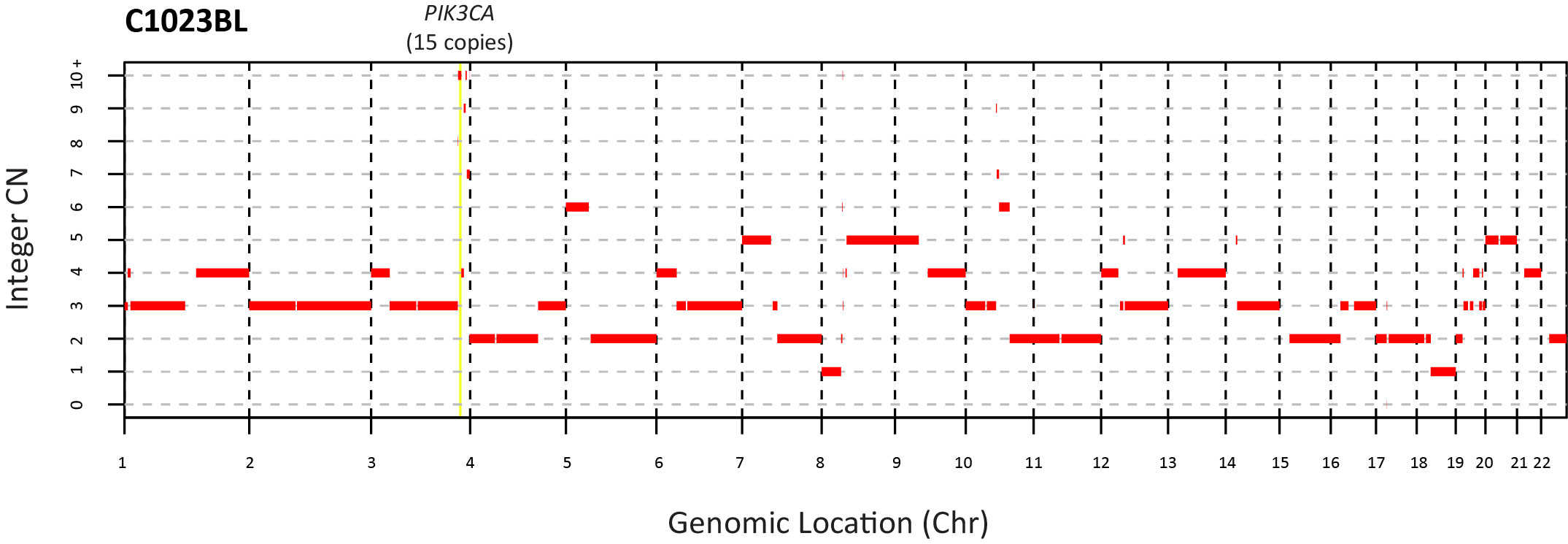


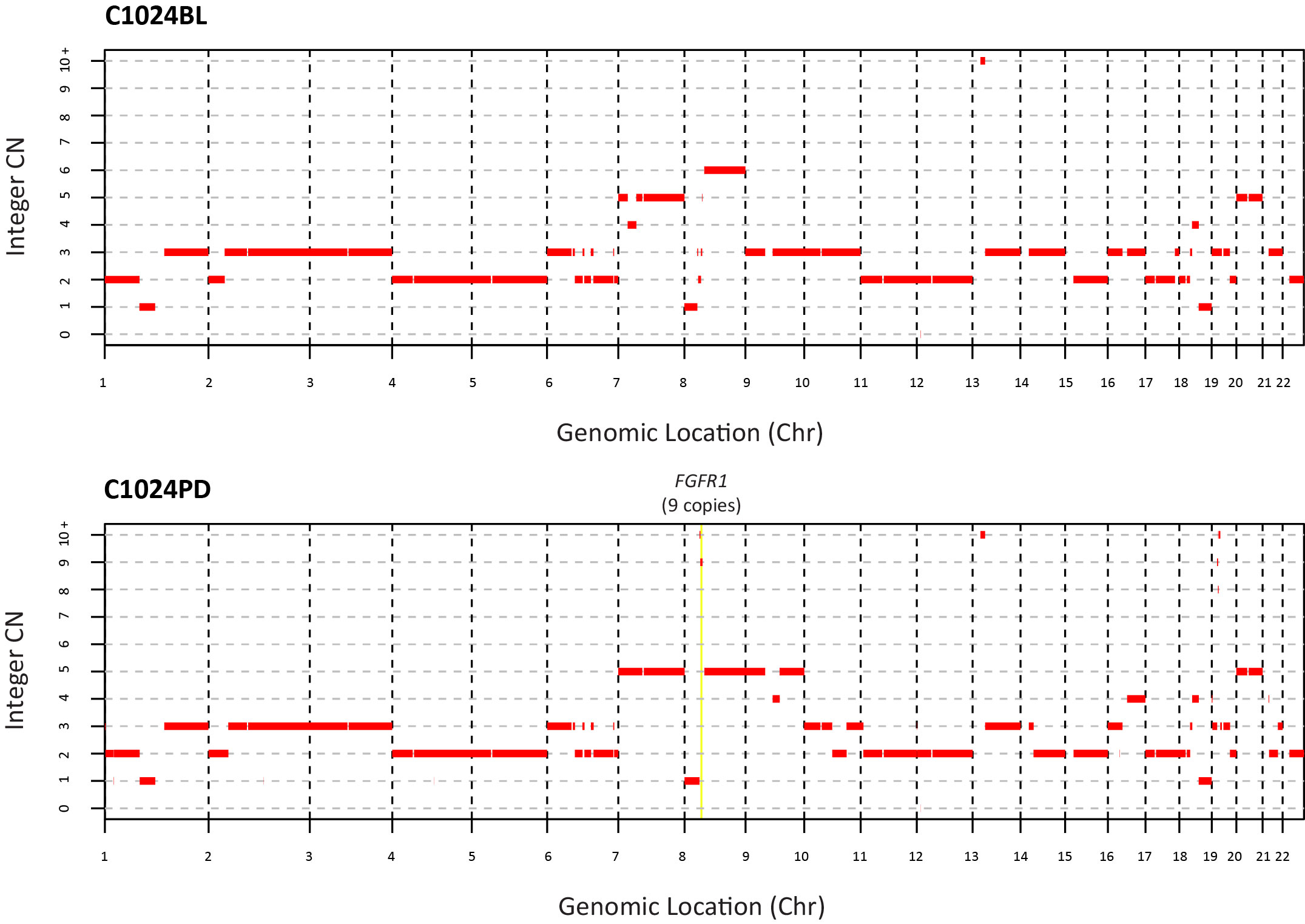


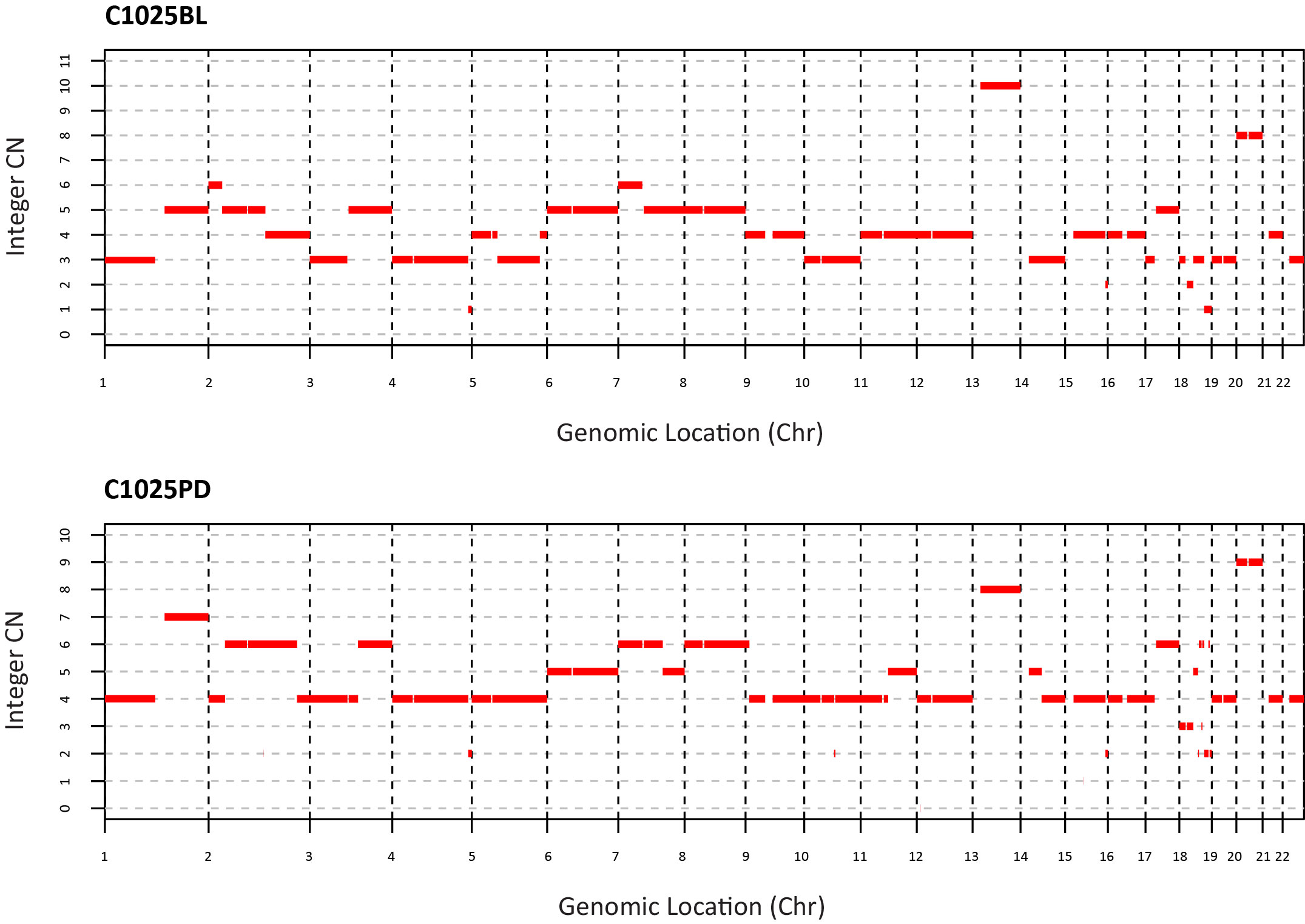


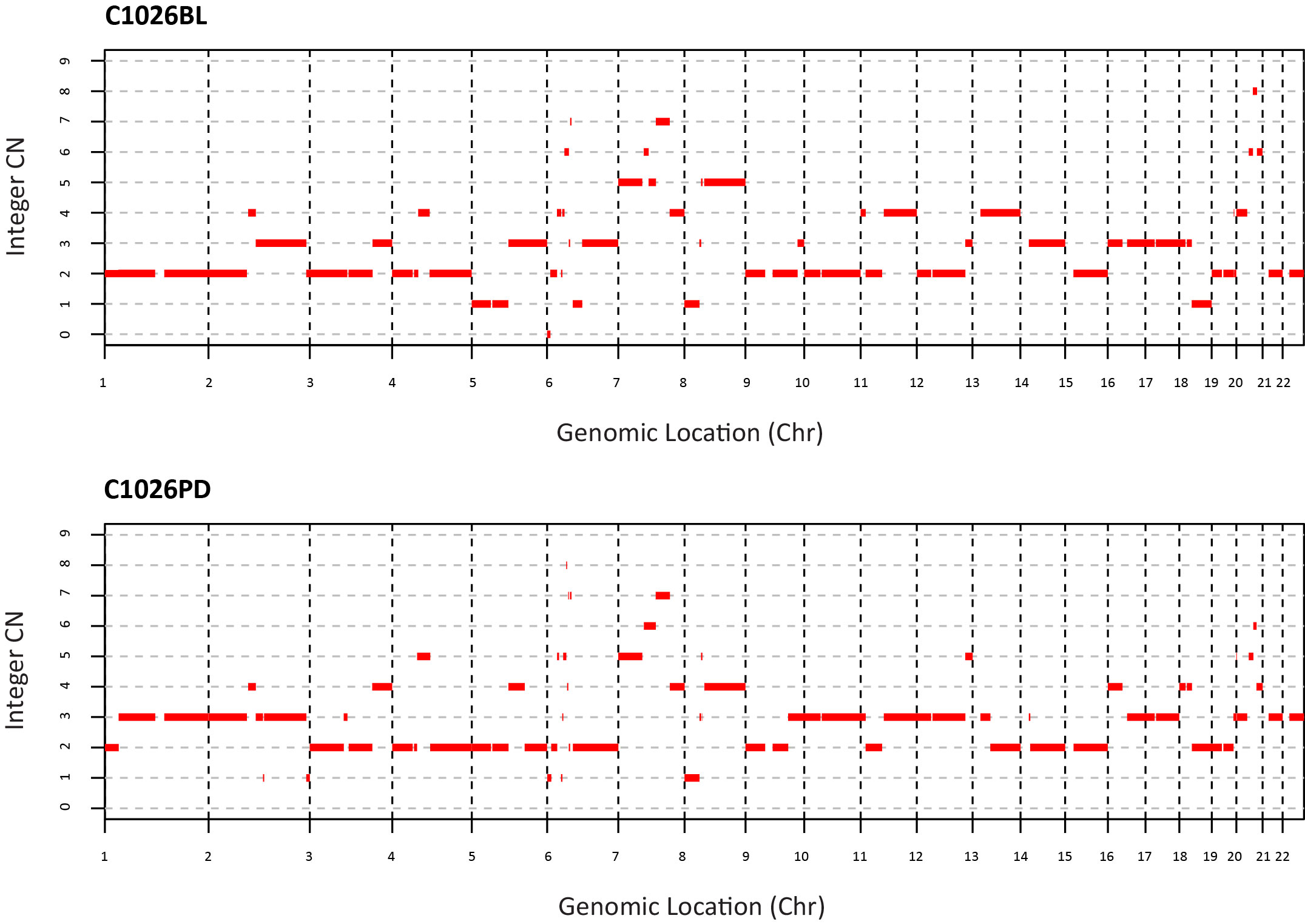


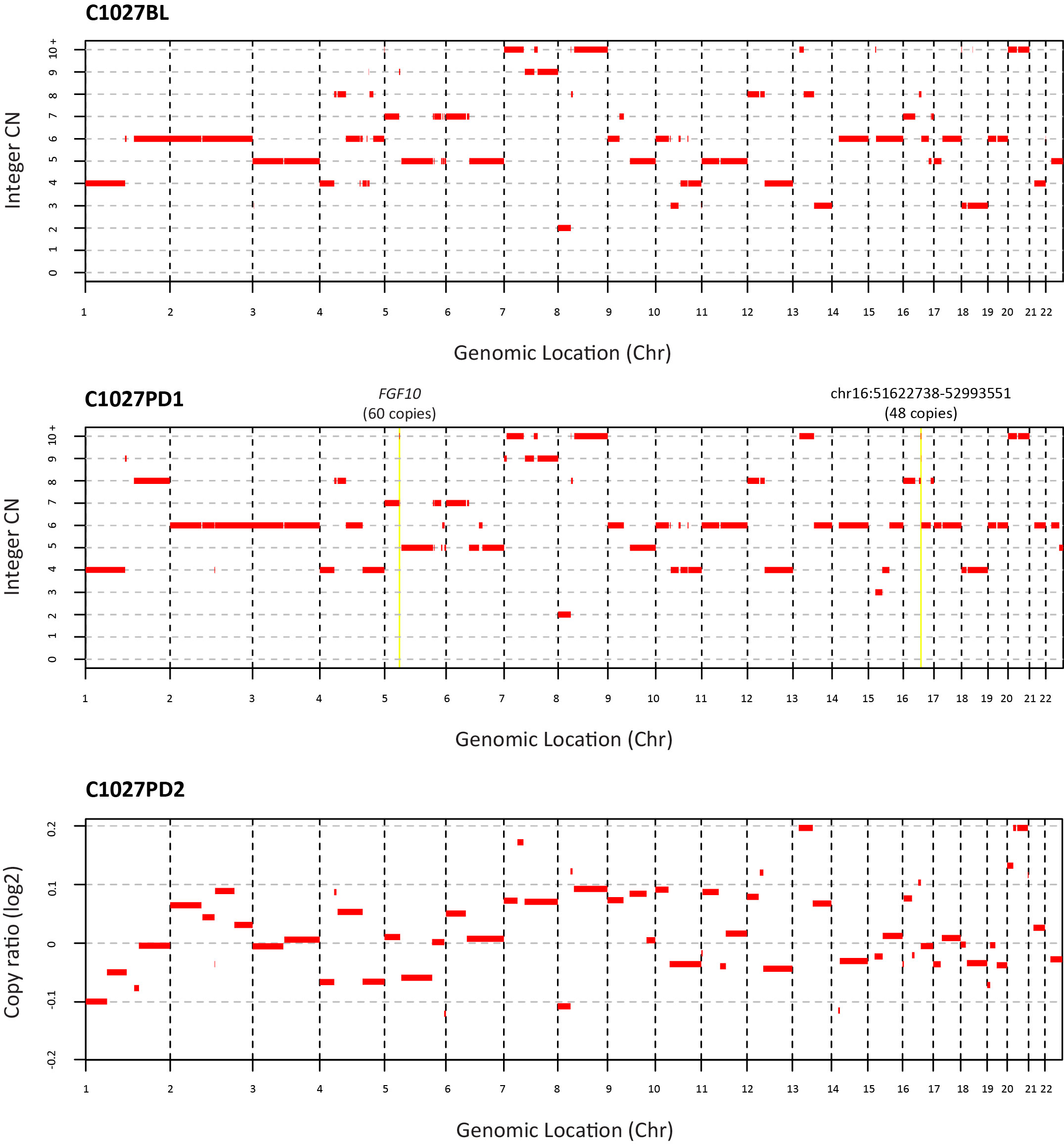


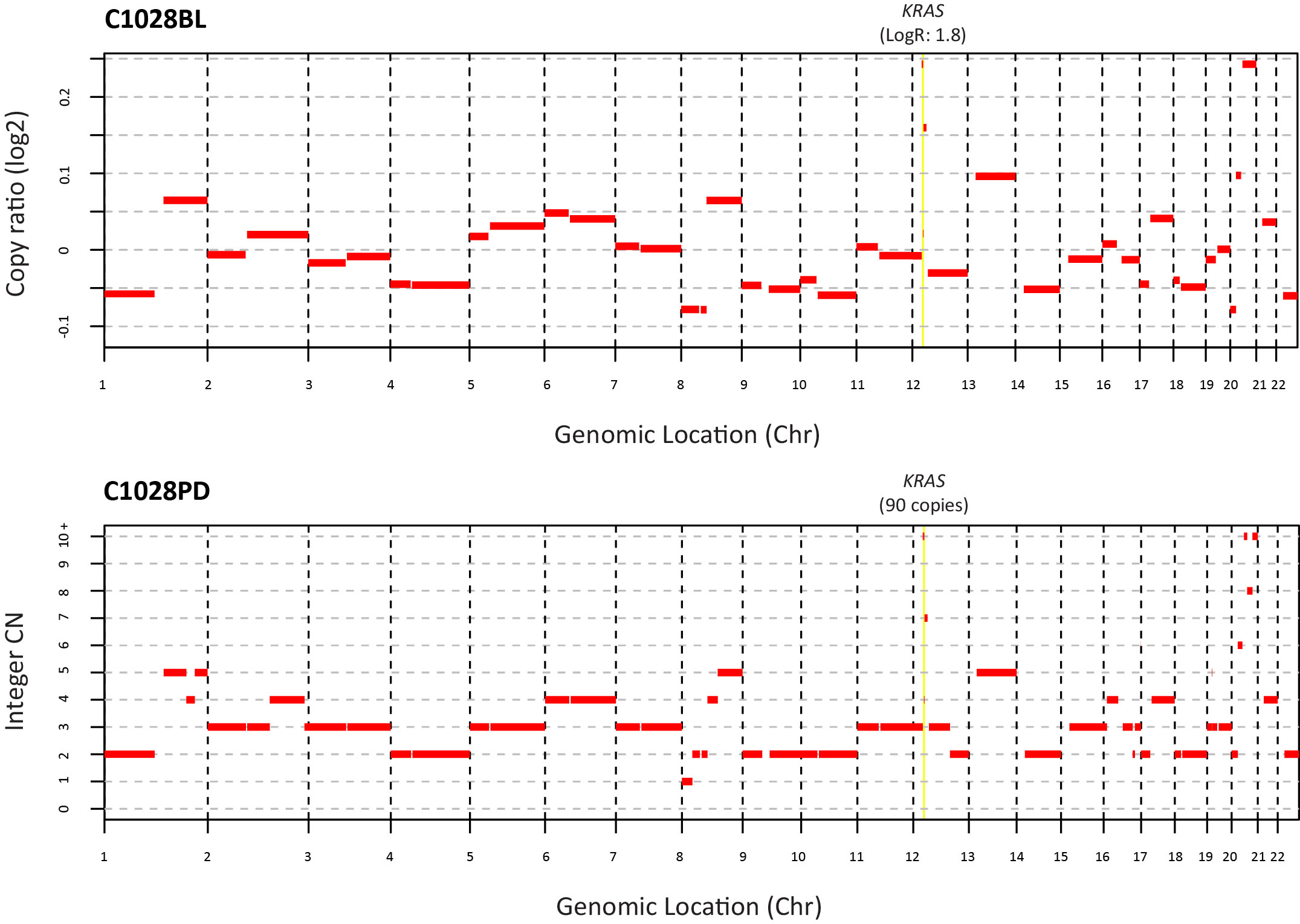


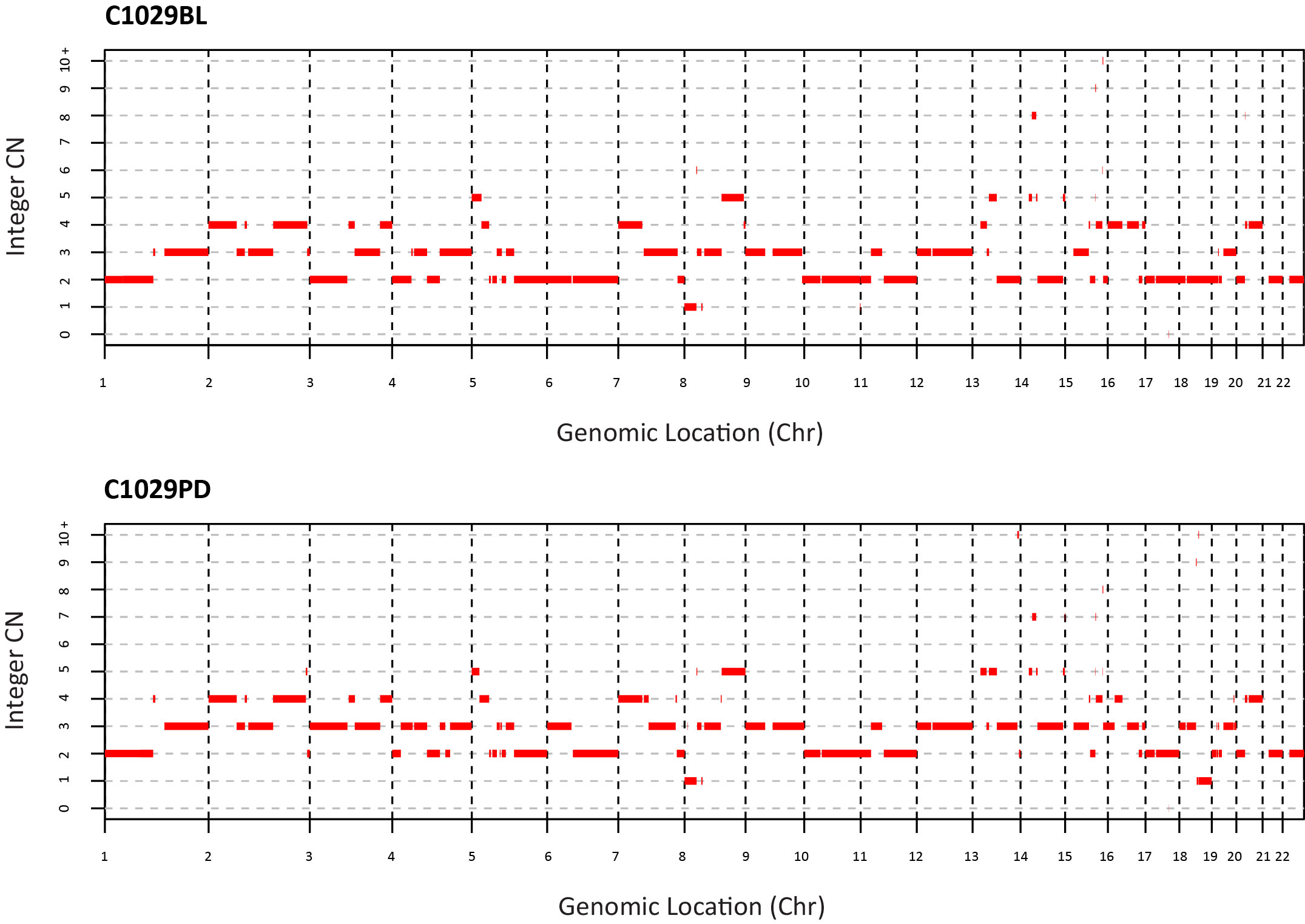


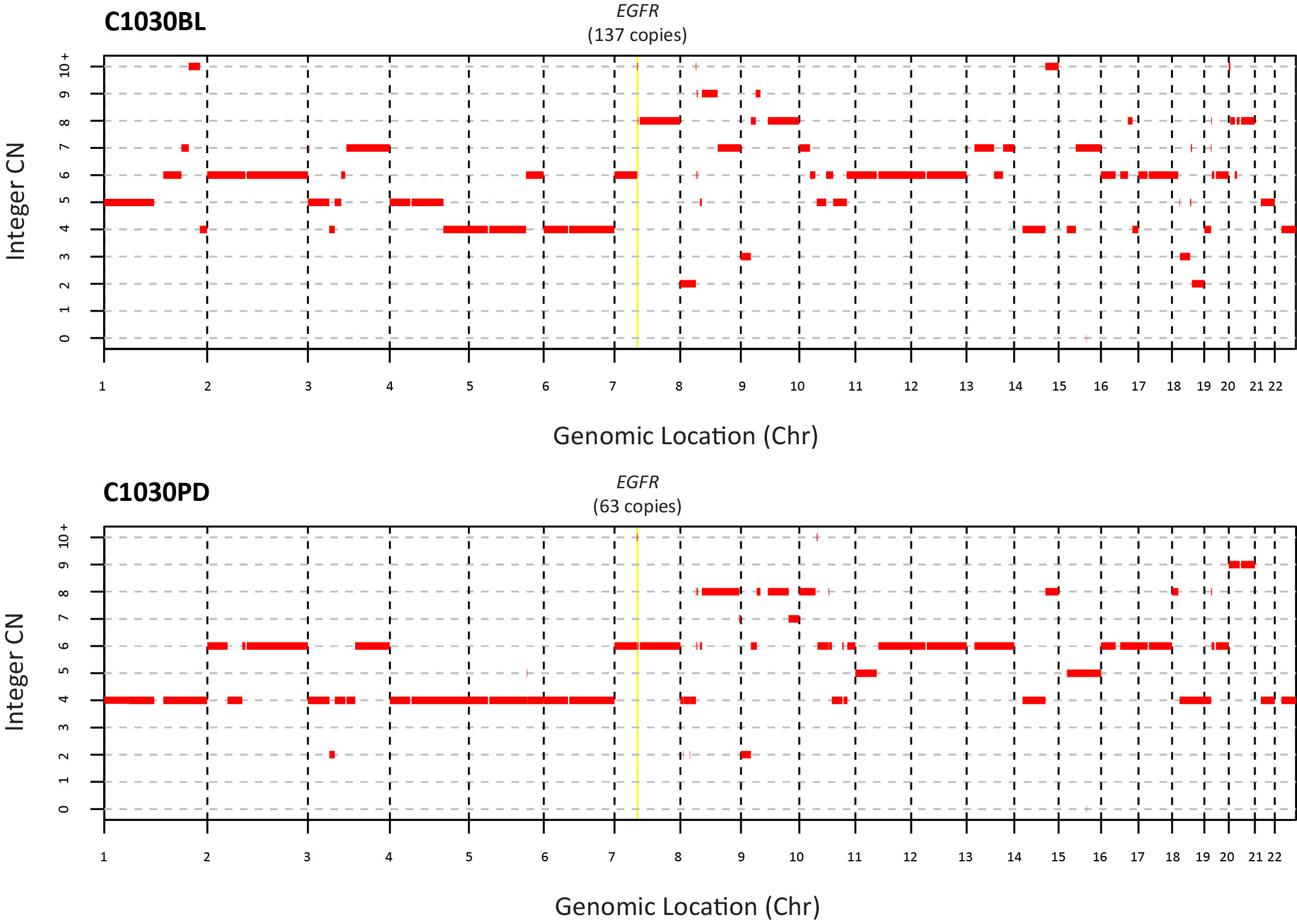


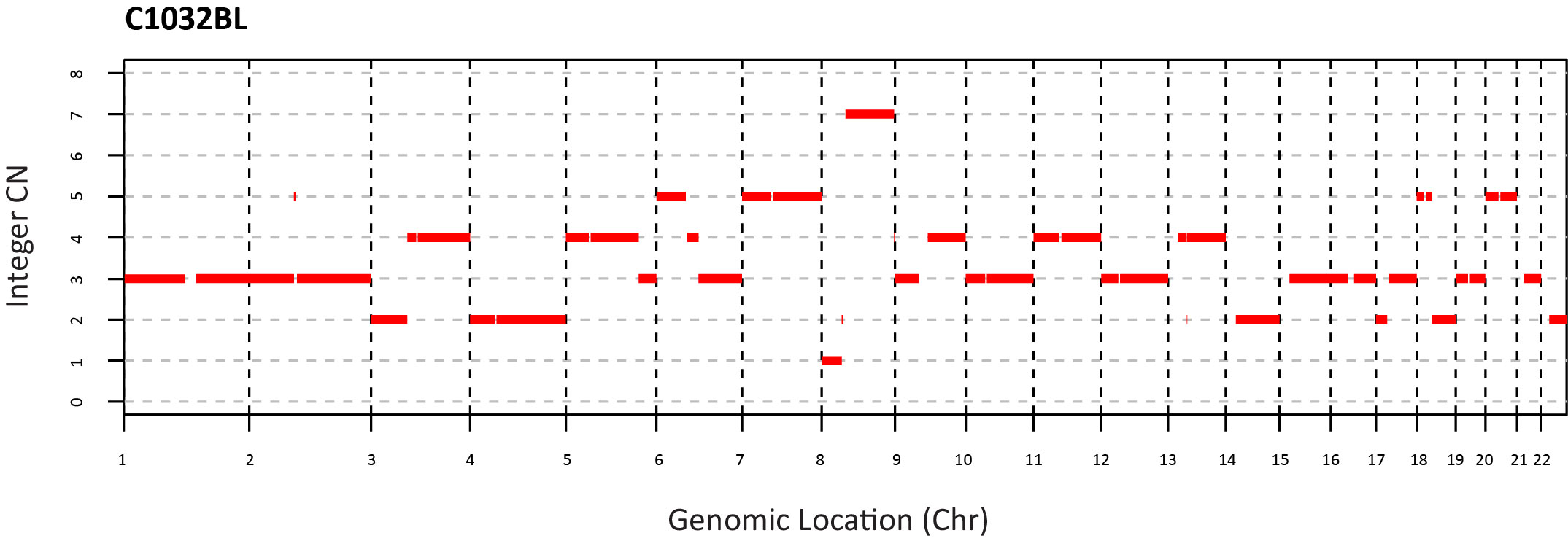


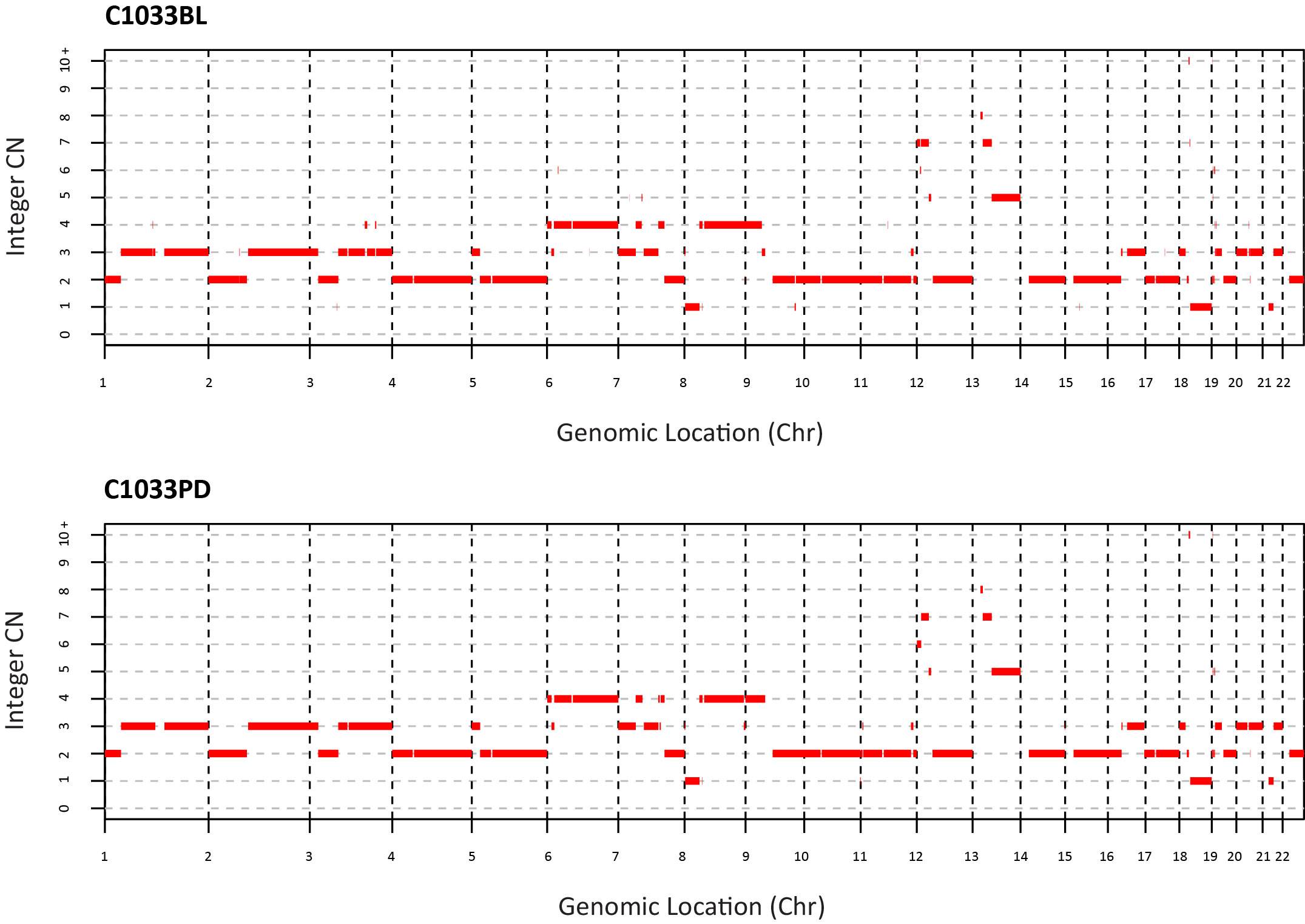


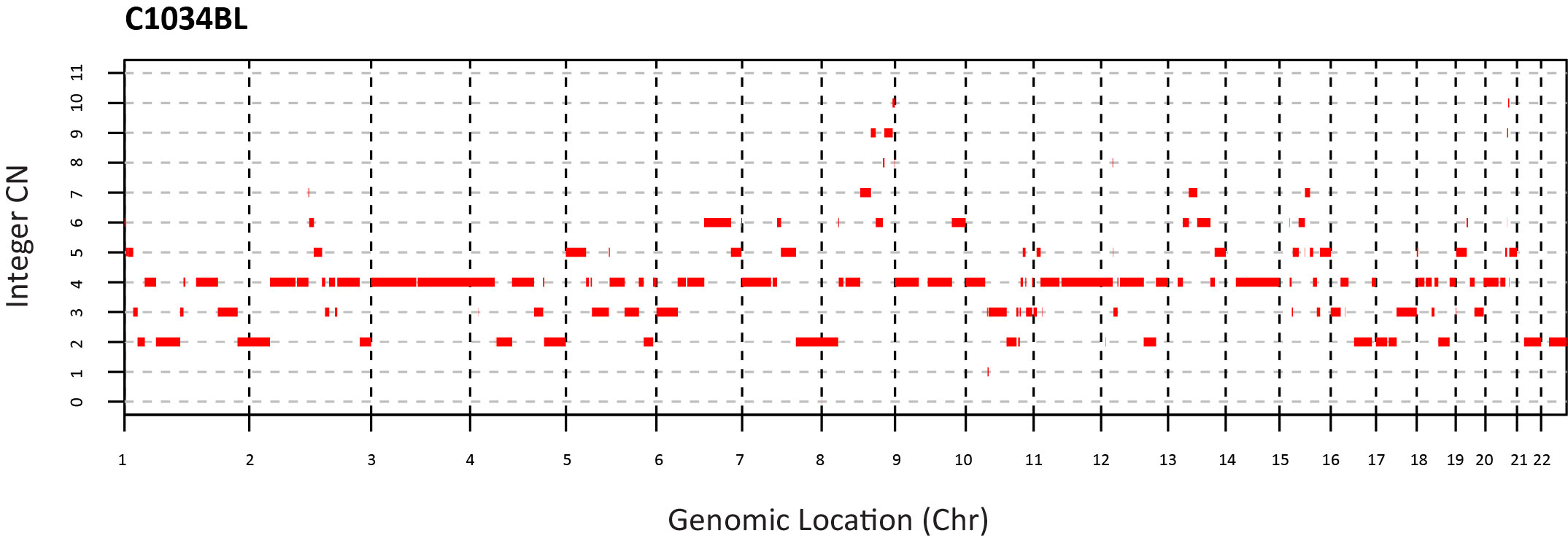


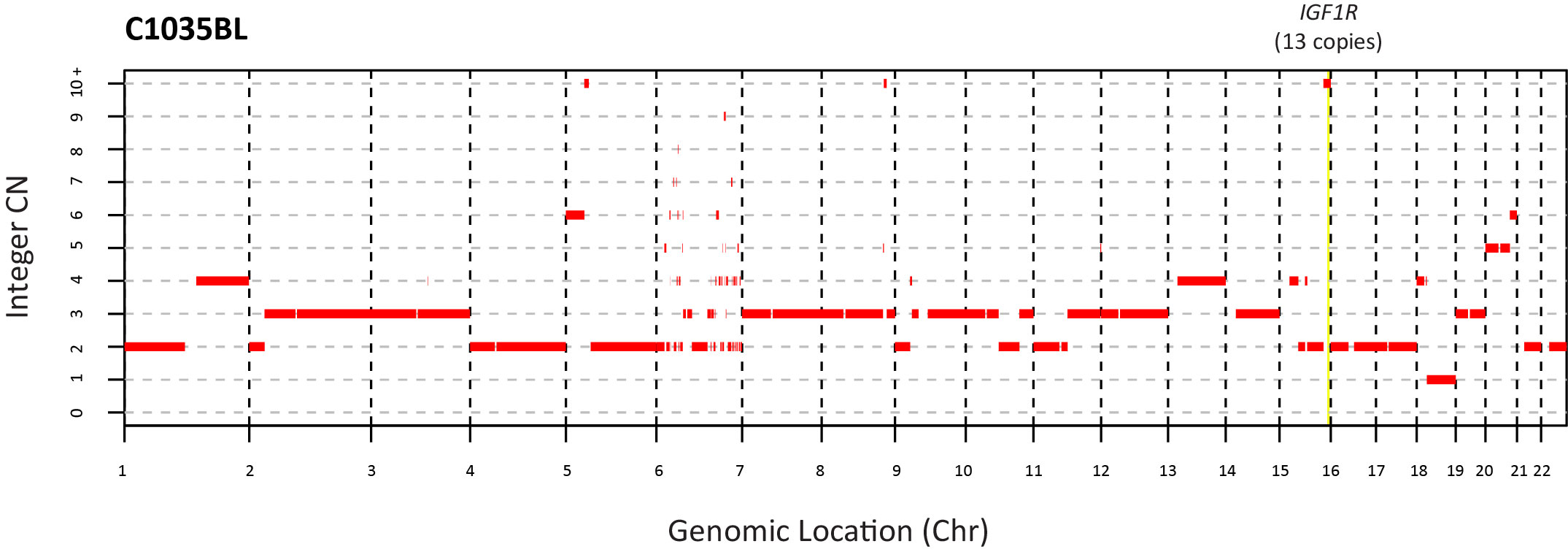


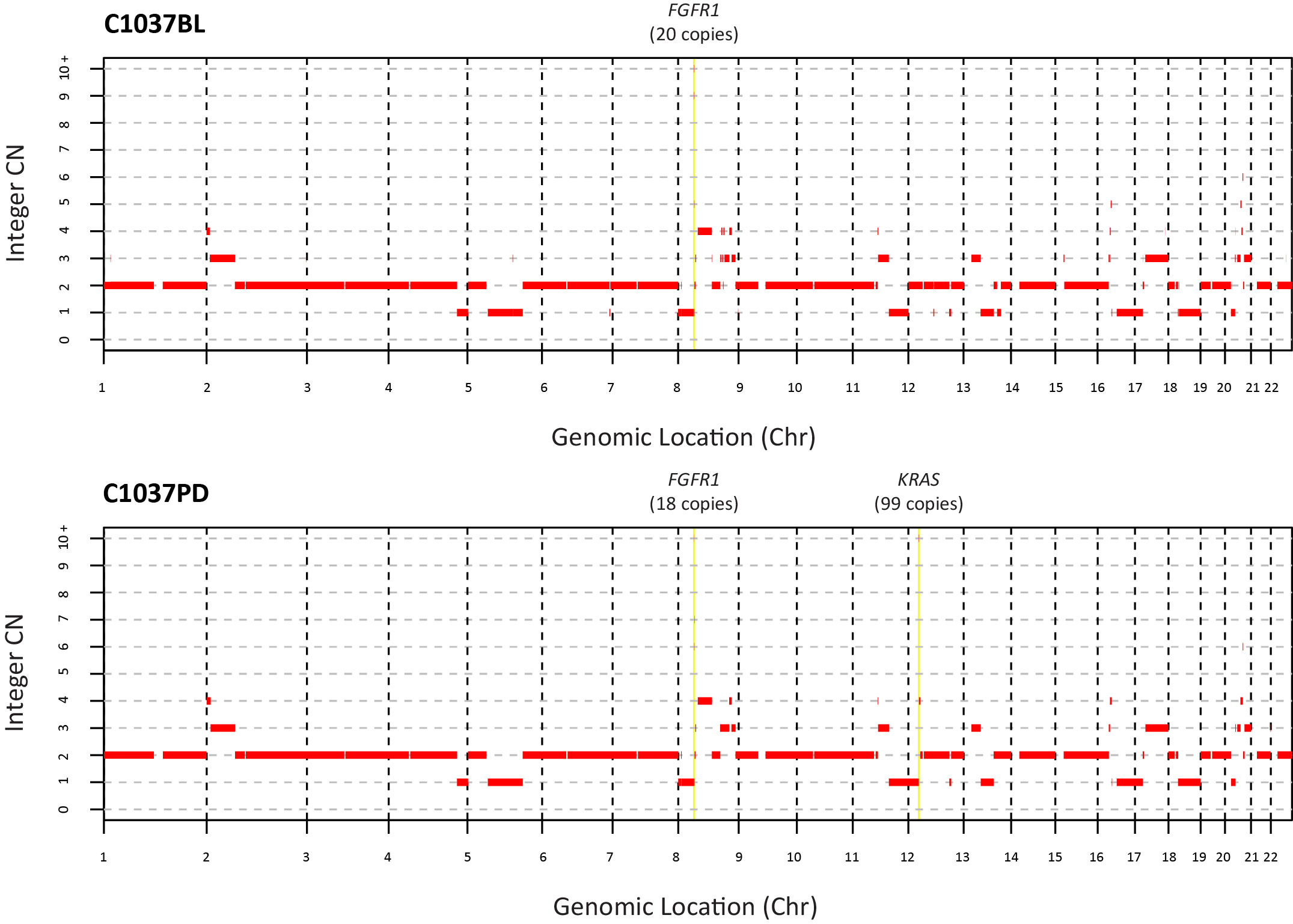


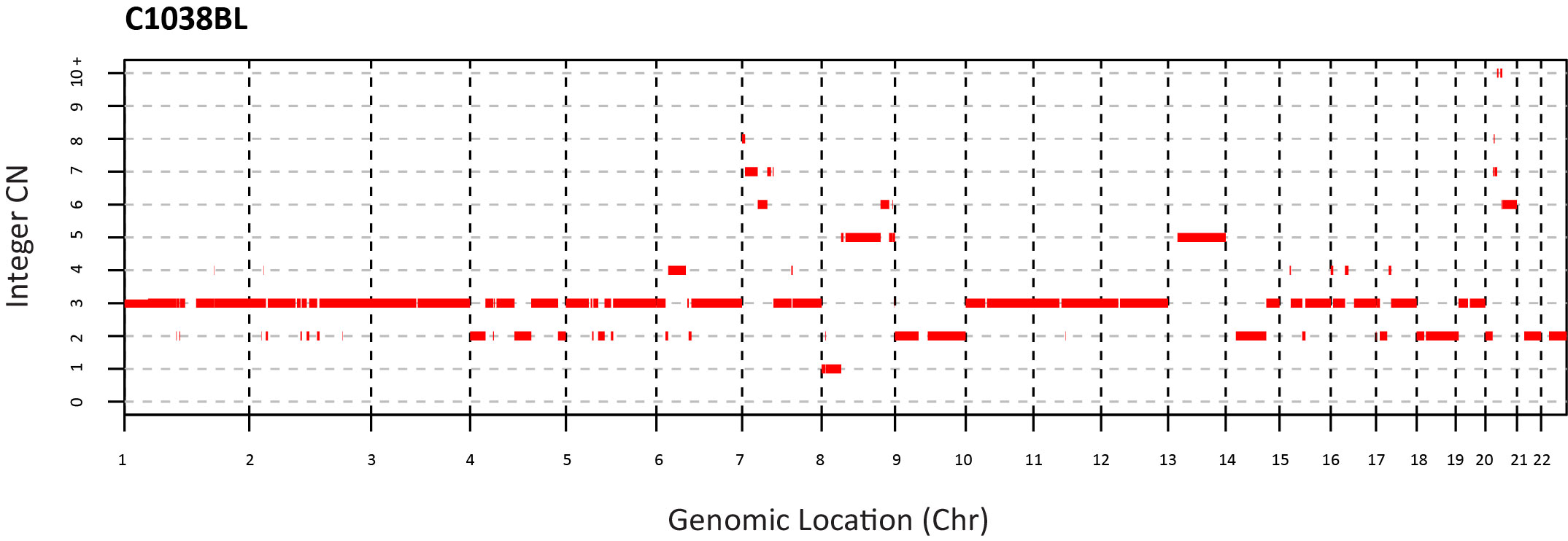


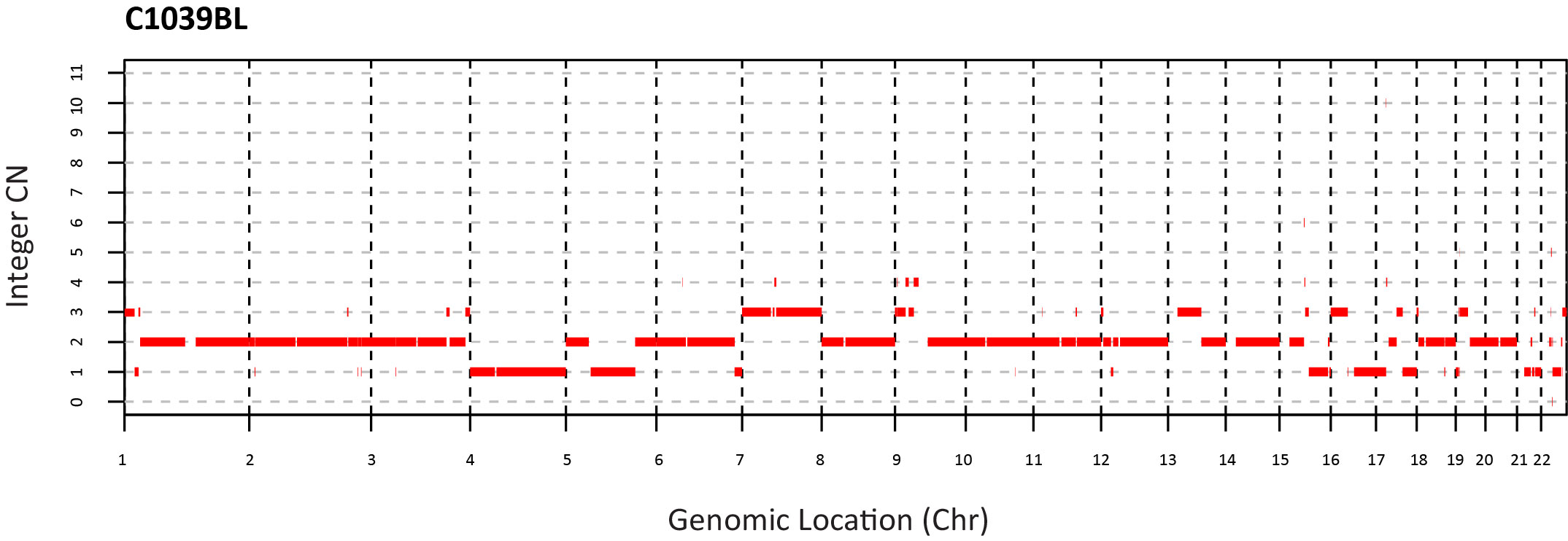


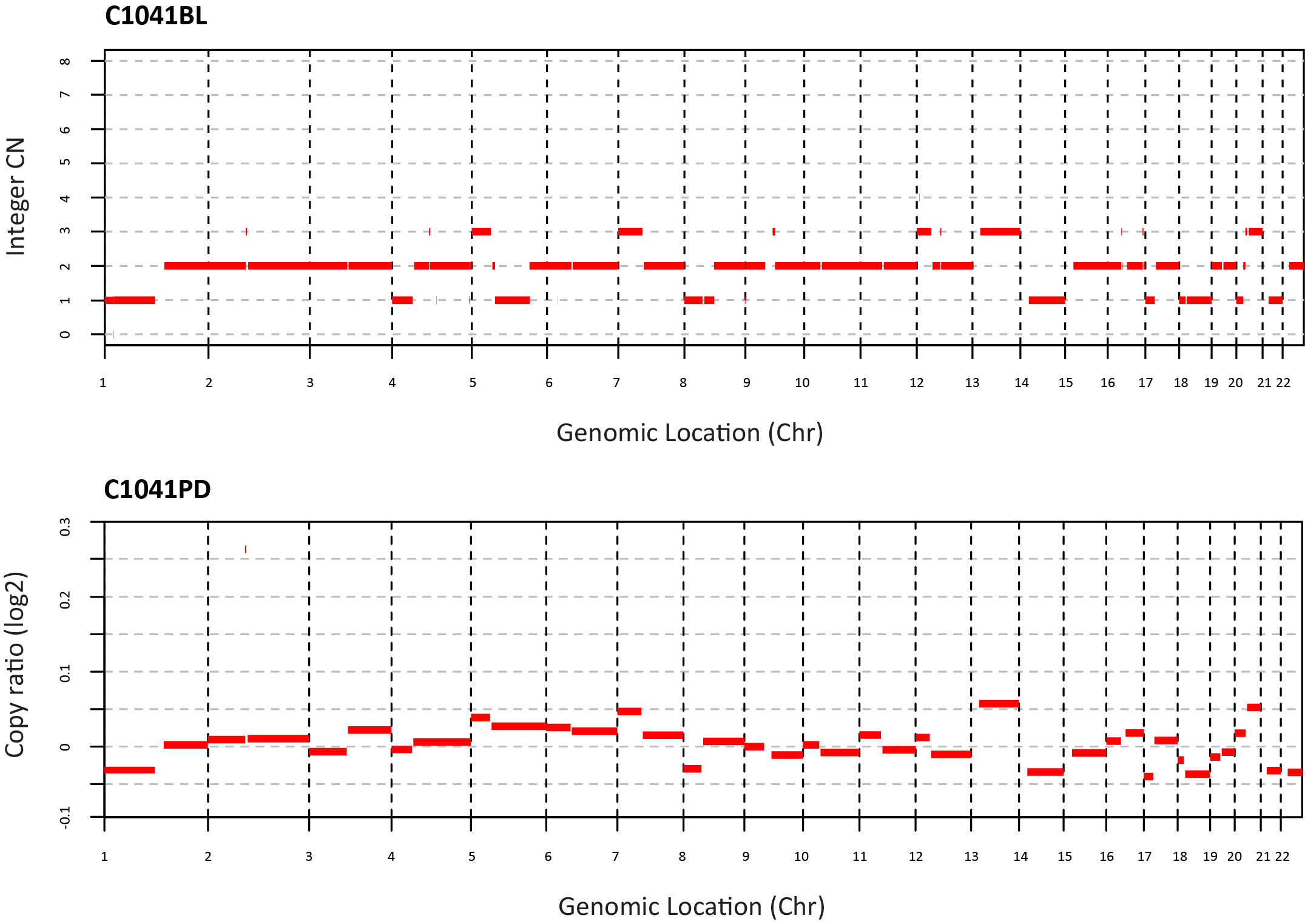


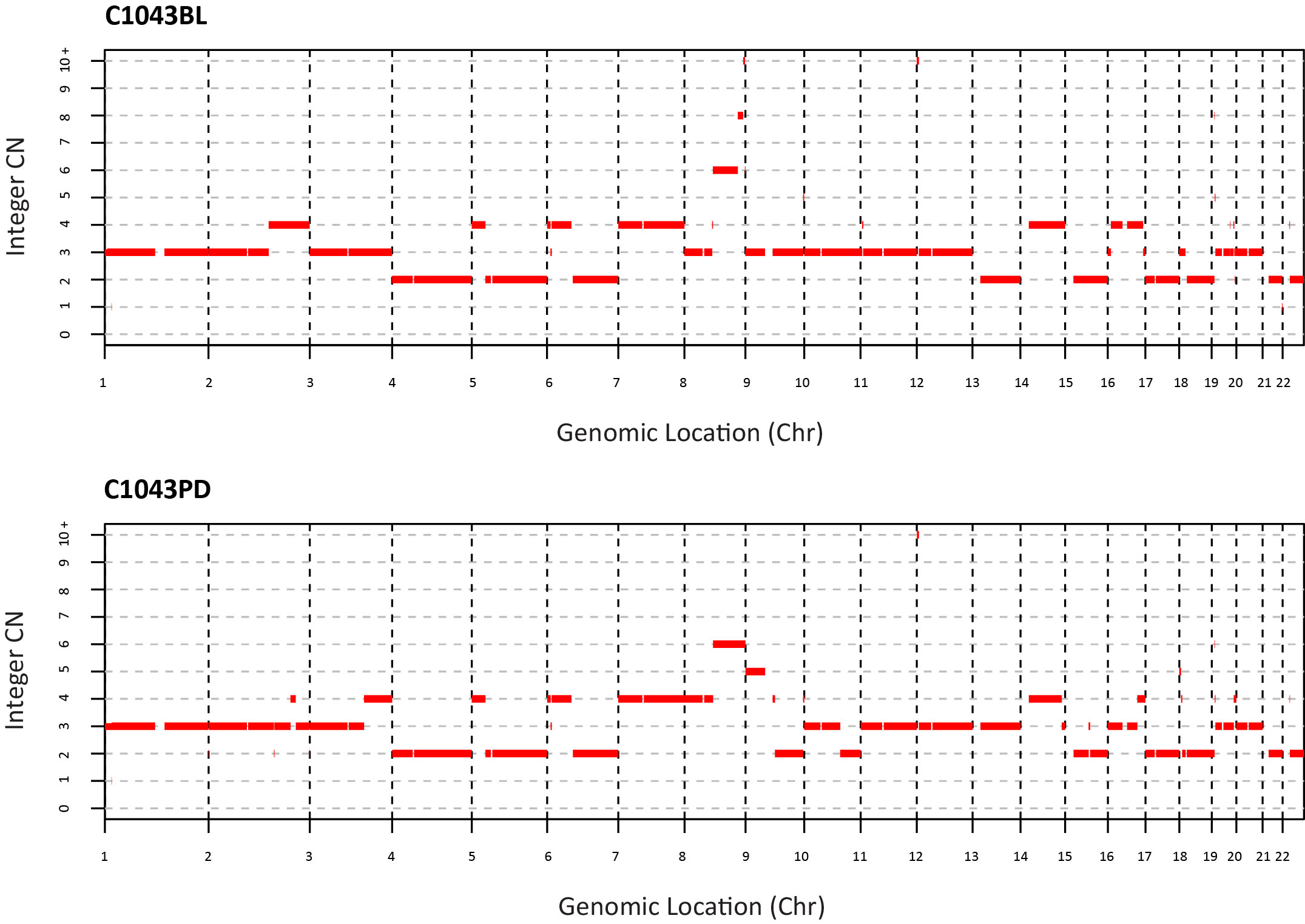


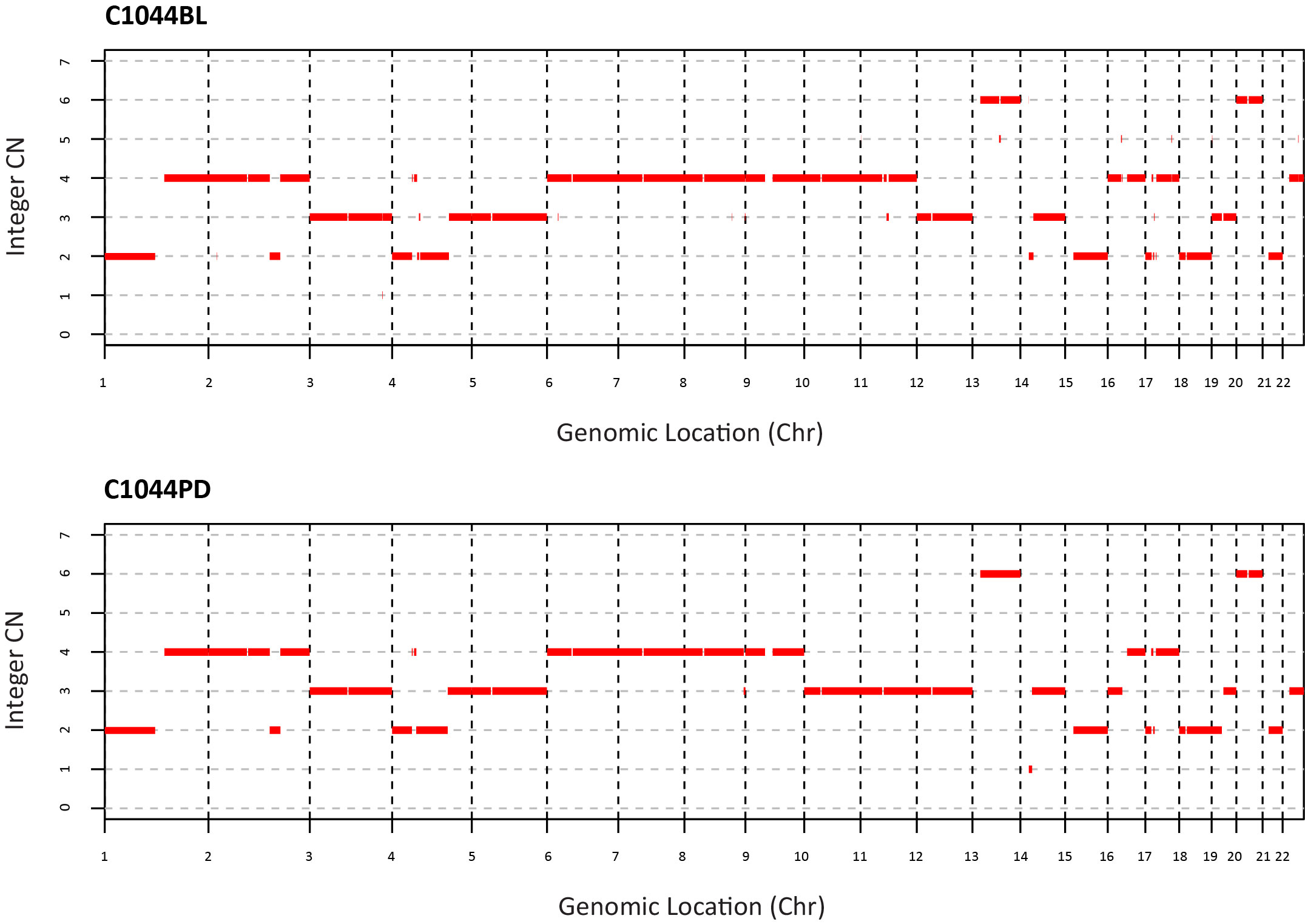


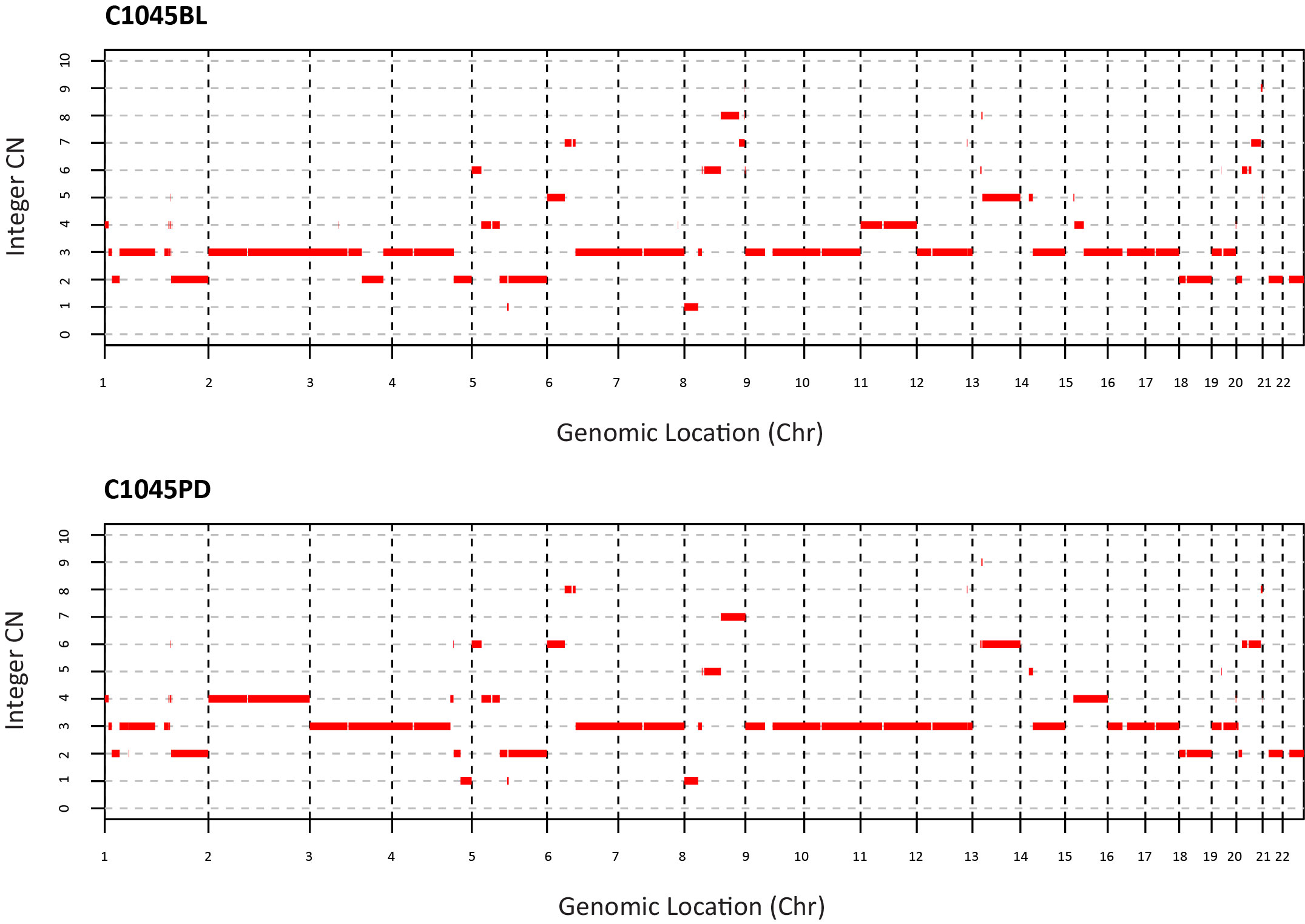


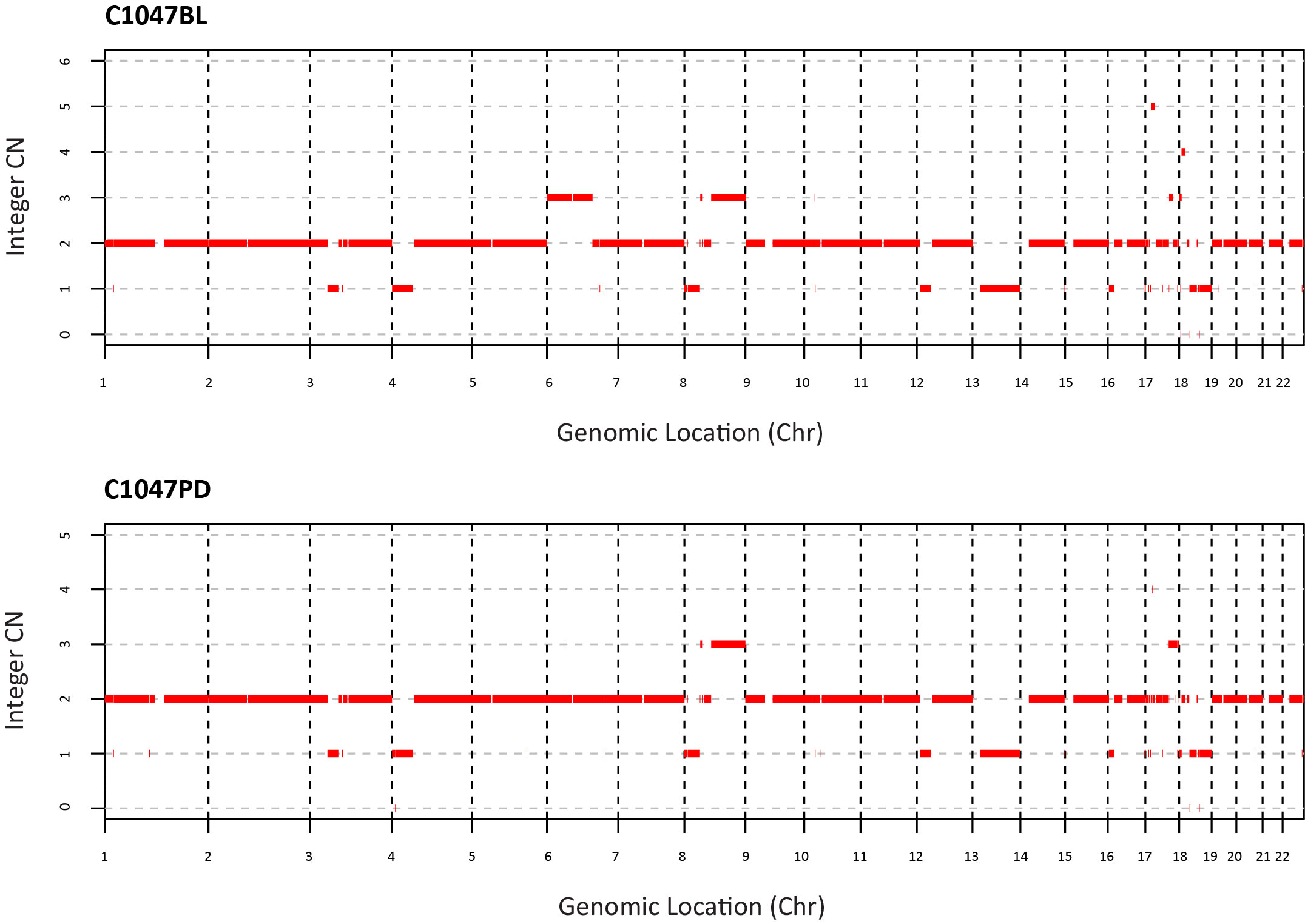

Supplement: Data S1. Genome-wide DNA Copy-Number Profiles of BL and PD Biopsies, Related to Figure 2 [file mmc7.zip › Data Set S1.docx]
