# Supplementary material for: Genomic and Transcriptomic Determinants of Therapy Resistance and Immune Landscape Evolution during Anti-EGFR Treatment in Colorectal Cancer
Source: Cancer Cell. 2019 Jul 8;36(1):35–50.e9. doi: 10.1016/j.ccell.2019.05.013 (PMC6617392; doi:10.1016/j.ccell.2019.05.013)
Supplement: Data S2. Mutation Clonality Assessment, Related to Figure 2 [file mmc8.zip › Data Set S2.docx]

## Data S2: Mutation clonality assessment. Related to Figure 2.

1. Polysomy of KRAS p.A18D mutation in C1033BL


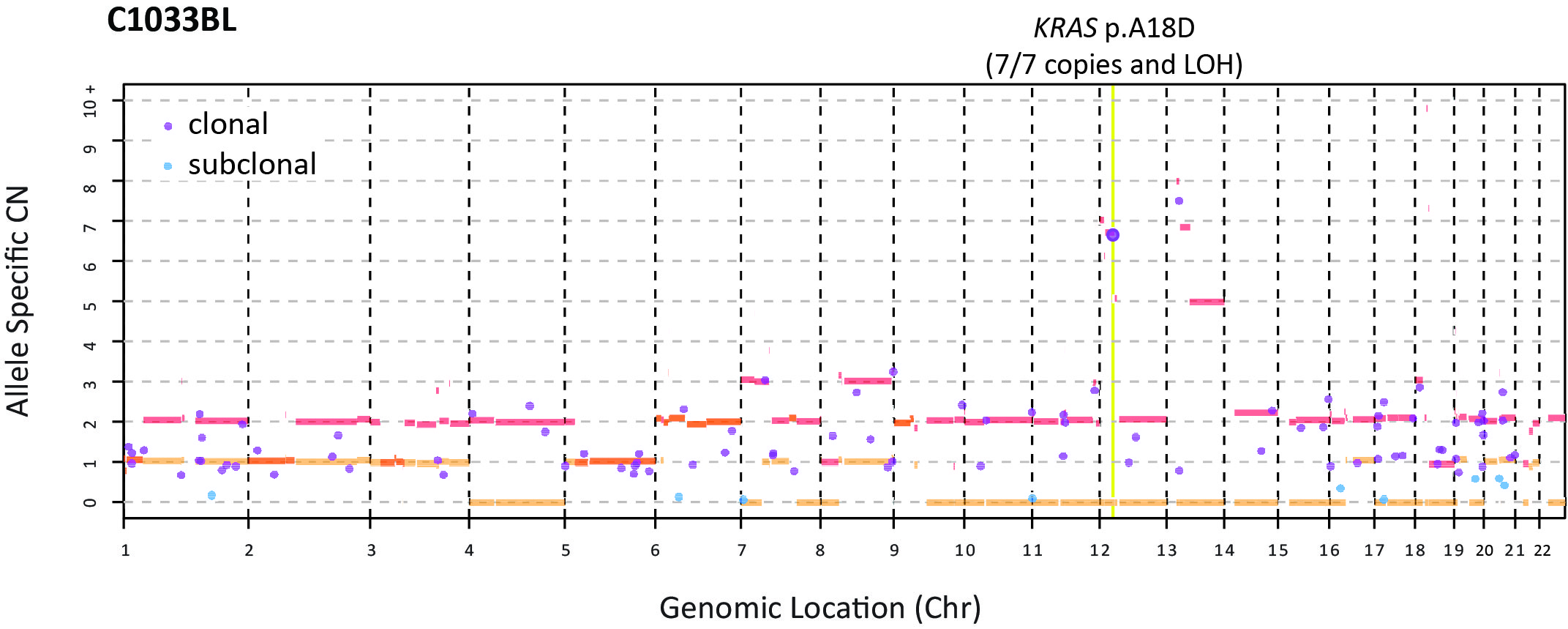


1. Biallelic loss of NF1 in C1021BL and C1045BL


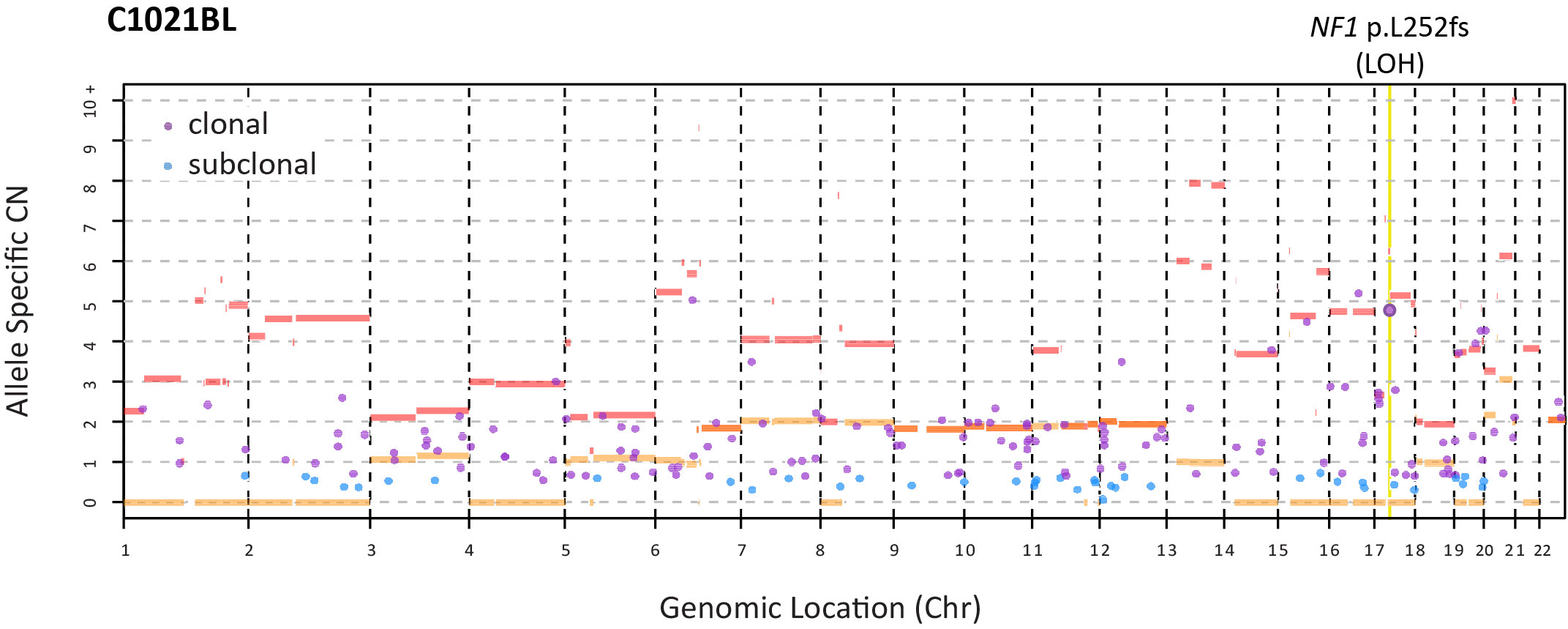


**
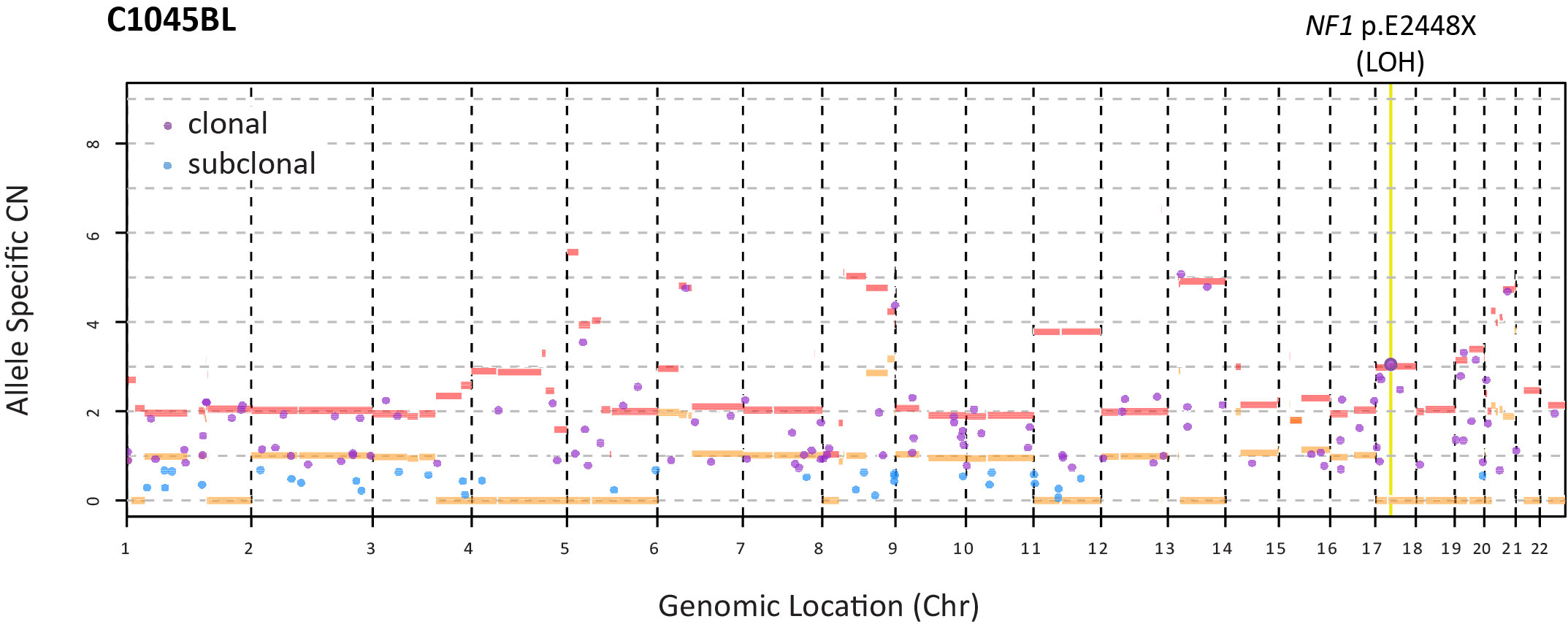
**

1. Concomitant PIK3CA mutation and amplification in C1023BL


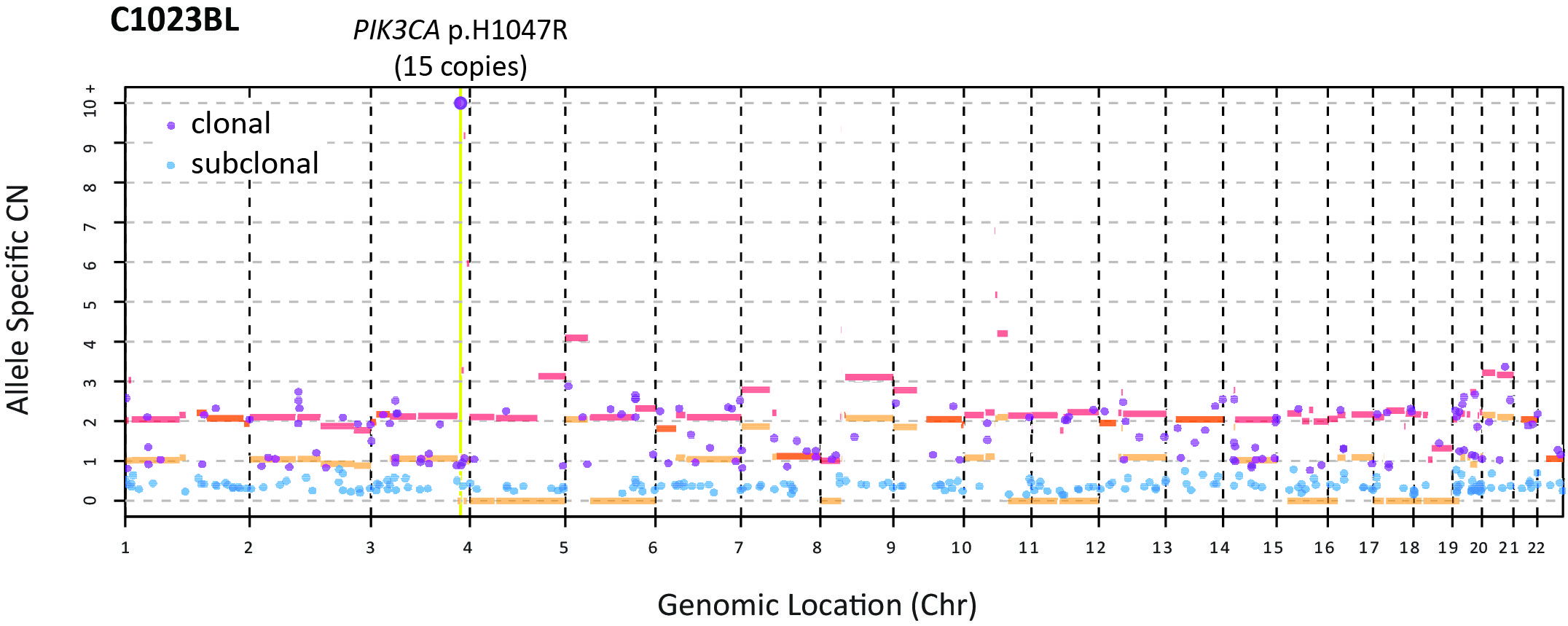


1. Evolution of clonal KRAS and EGFR mutations in PD biopsies in two cases


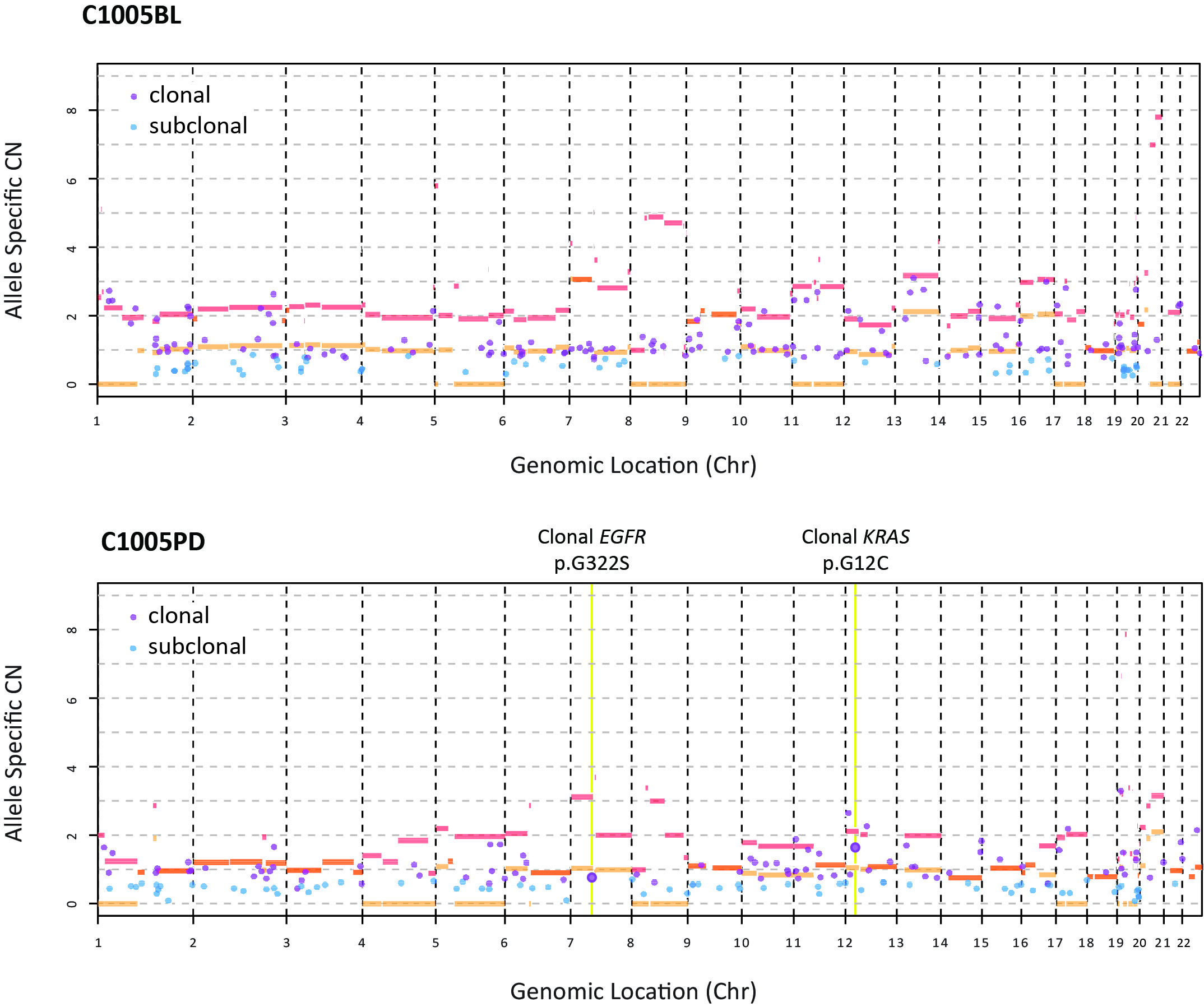


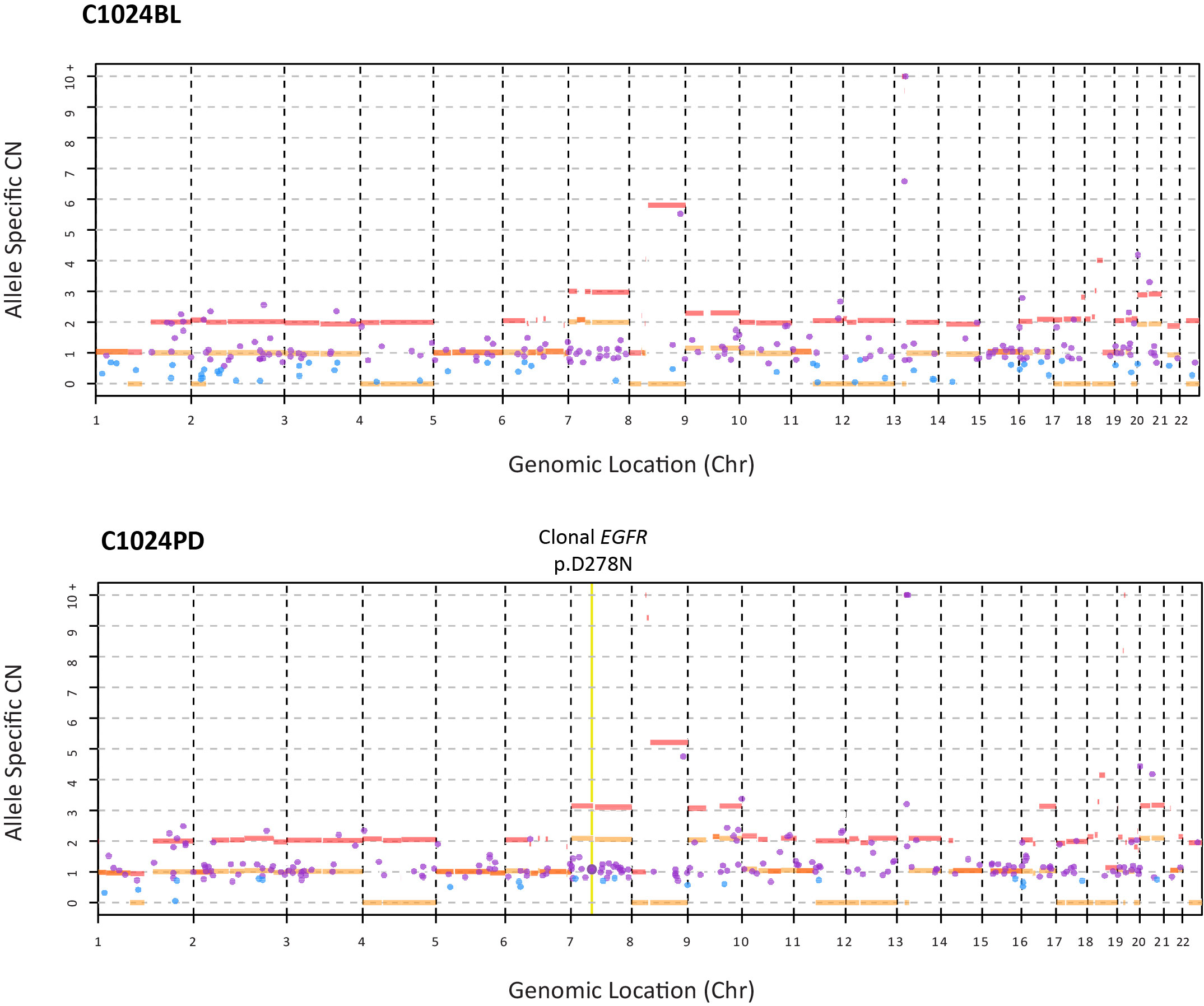


1. Evolution of subclonal KRAS and EGFR mutations in PD biopsies in two cases.
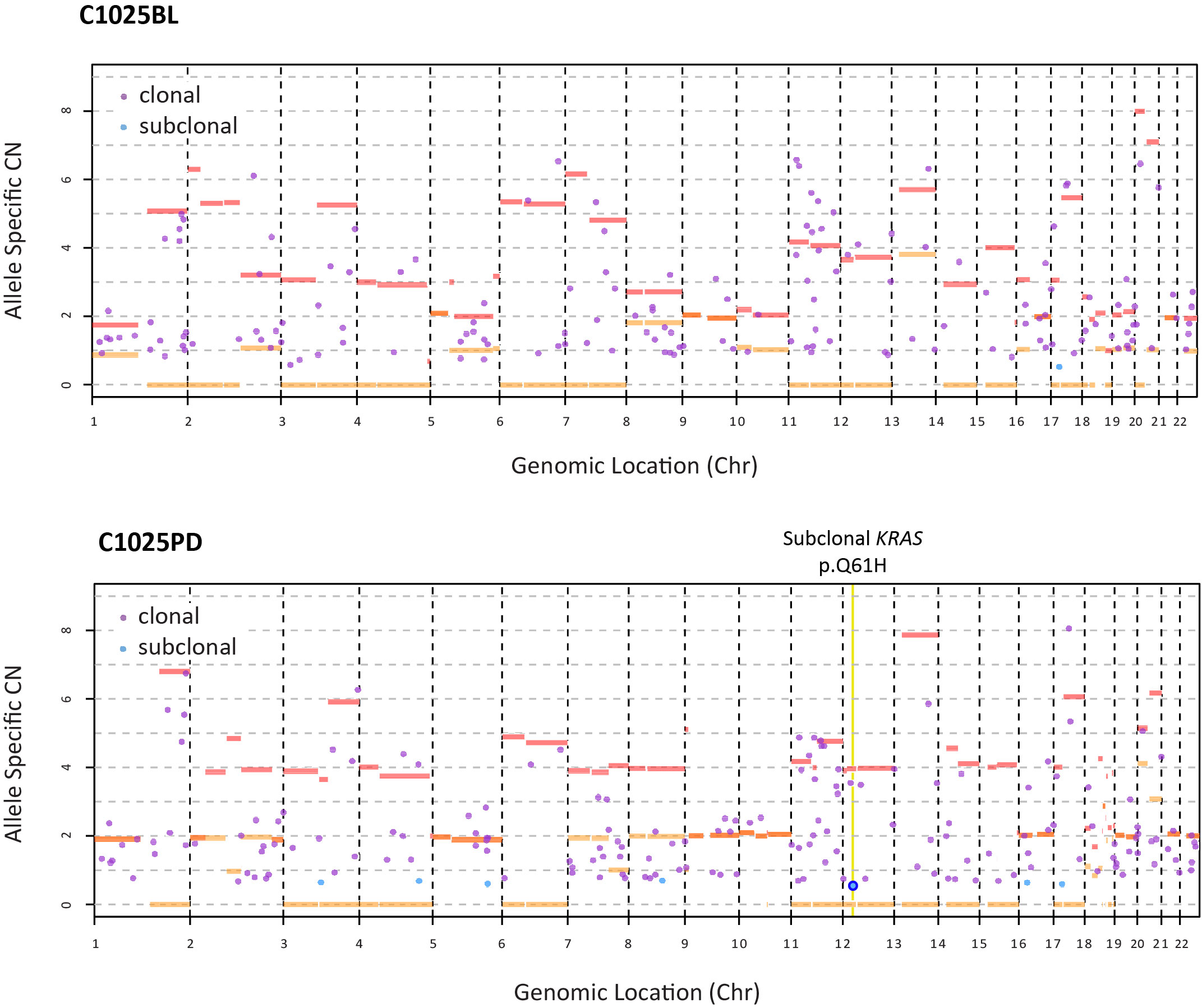


**
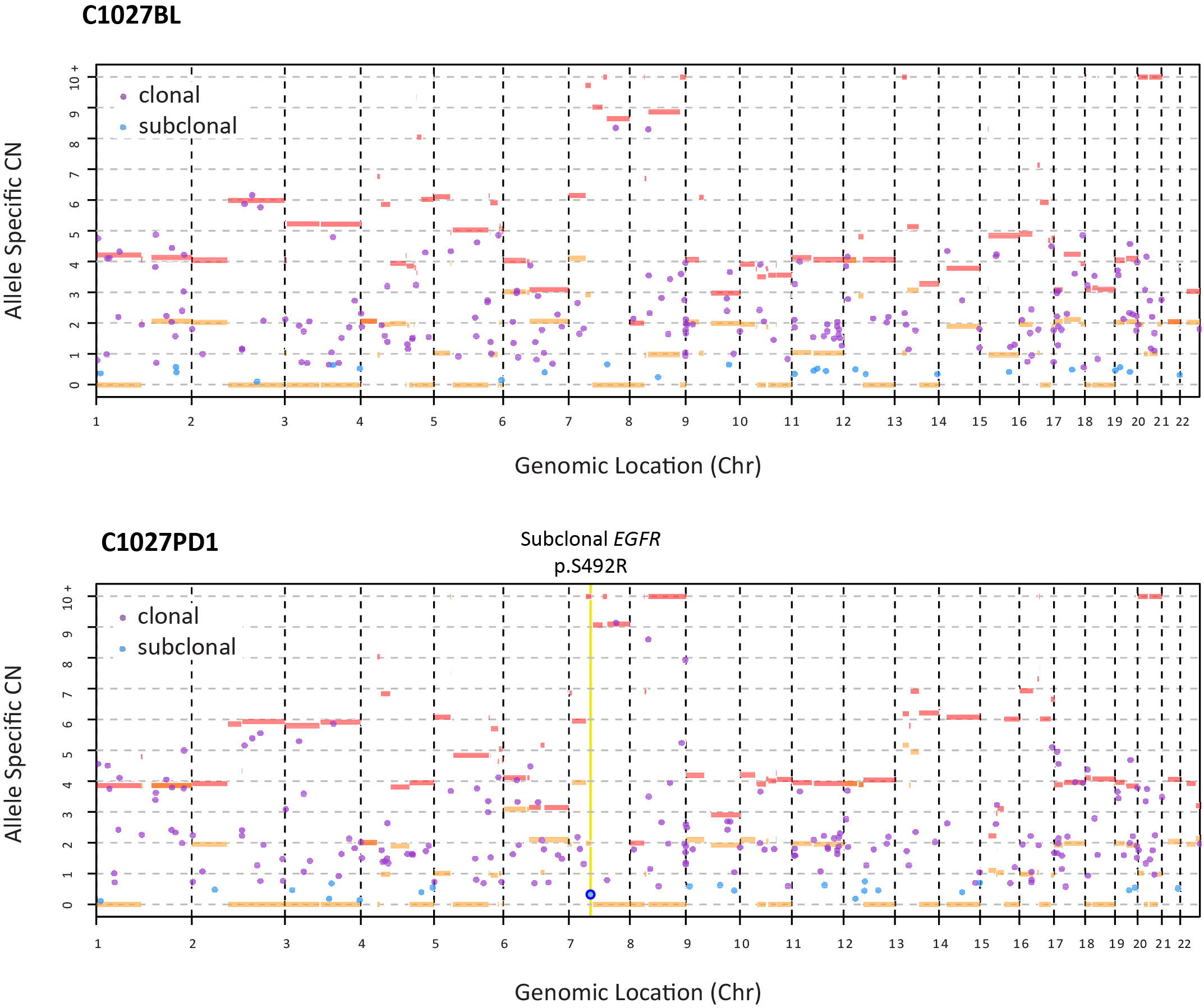
**
