# Supplementary material for: Genomic and Transcriptomic Determinants of Therapy Resistance and Immune Landscape Evolution during Anti-EGFR Treatment in Colorectal Cancer
Source: Cancer Cell. 2019 Jul 8;36(1):35–50.e9. doi: 10.1016/j.ccell.2019.05.013 (PMC6617392; doi:10.1016/j.ccell.2019.05.013)

## Data S3: Copy number profile comparison of biopsy and ctDNA samples. Related to Figure 5.Amplifications acquired at PD that harbour genes encoding for RTKs or members of the RAS/RAF-pathway are labelled with this gene, those not harboring such genes are labelled with the genomic coordinates of the amplicon.


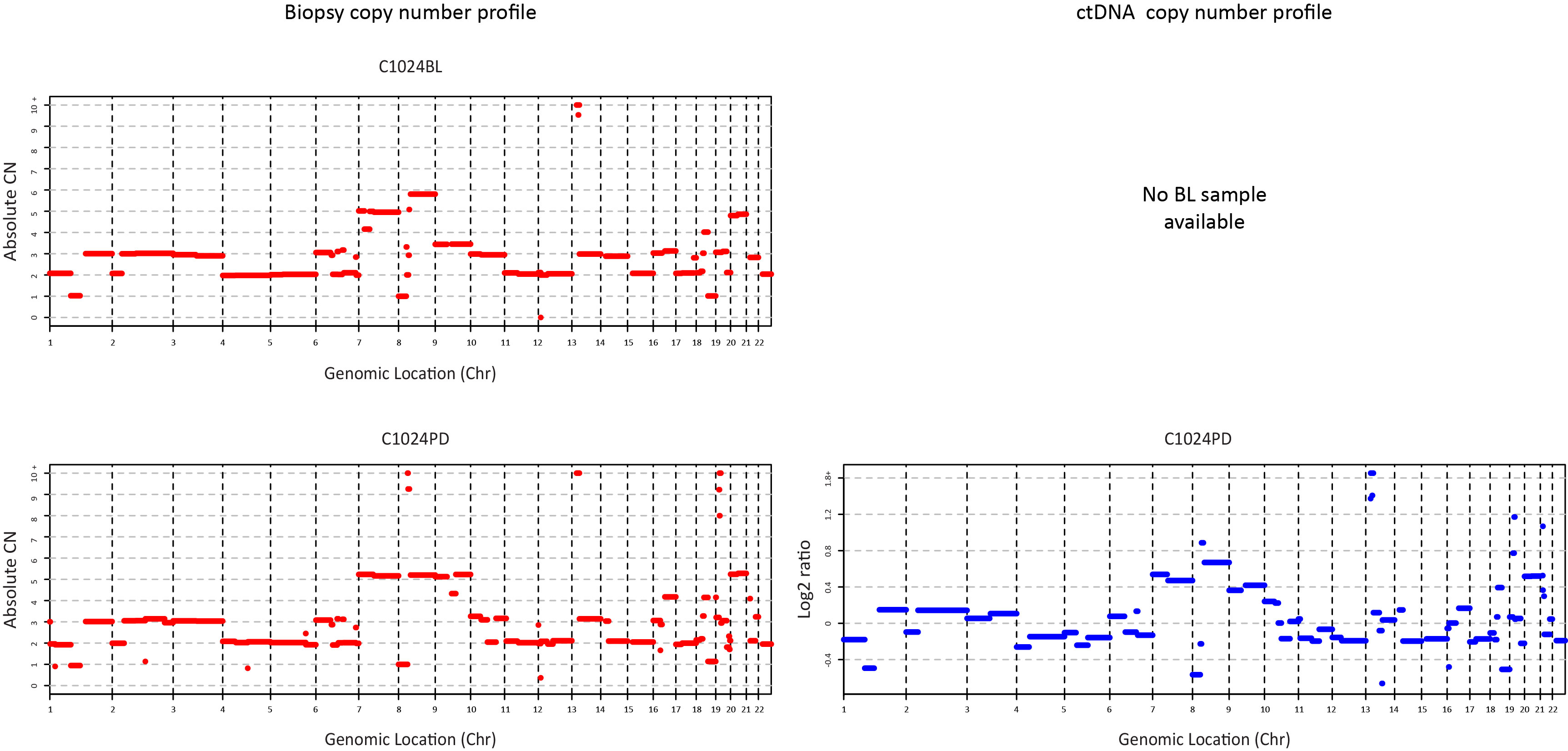


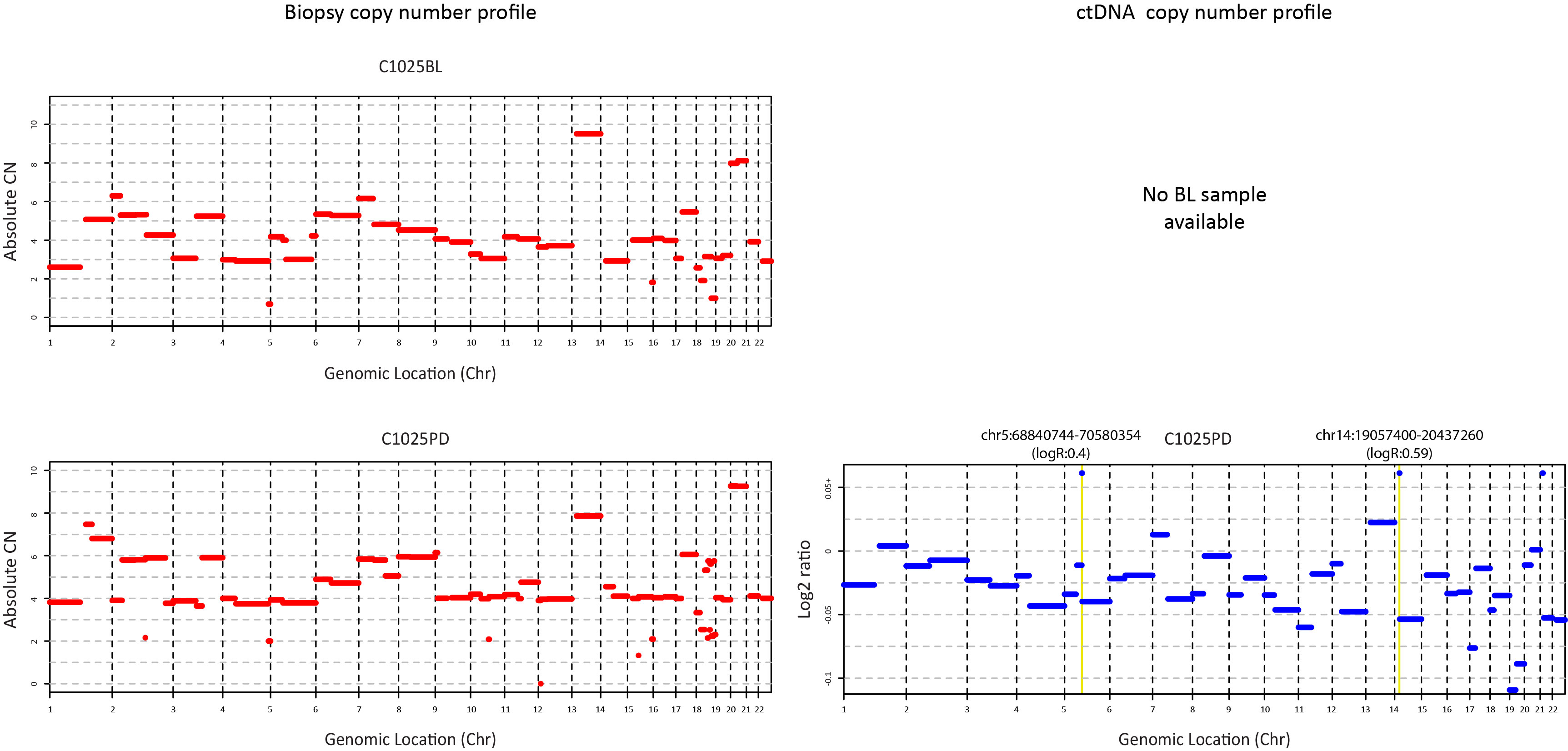


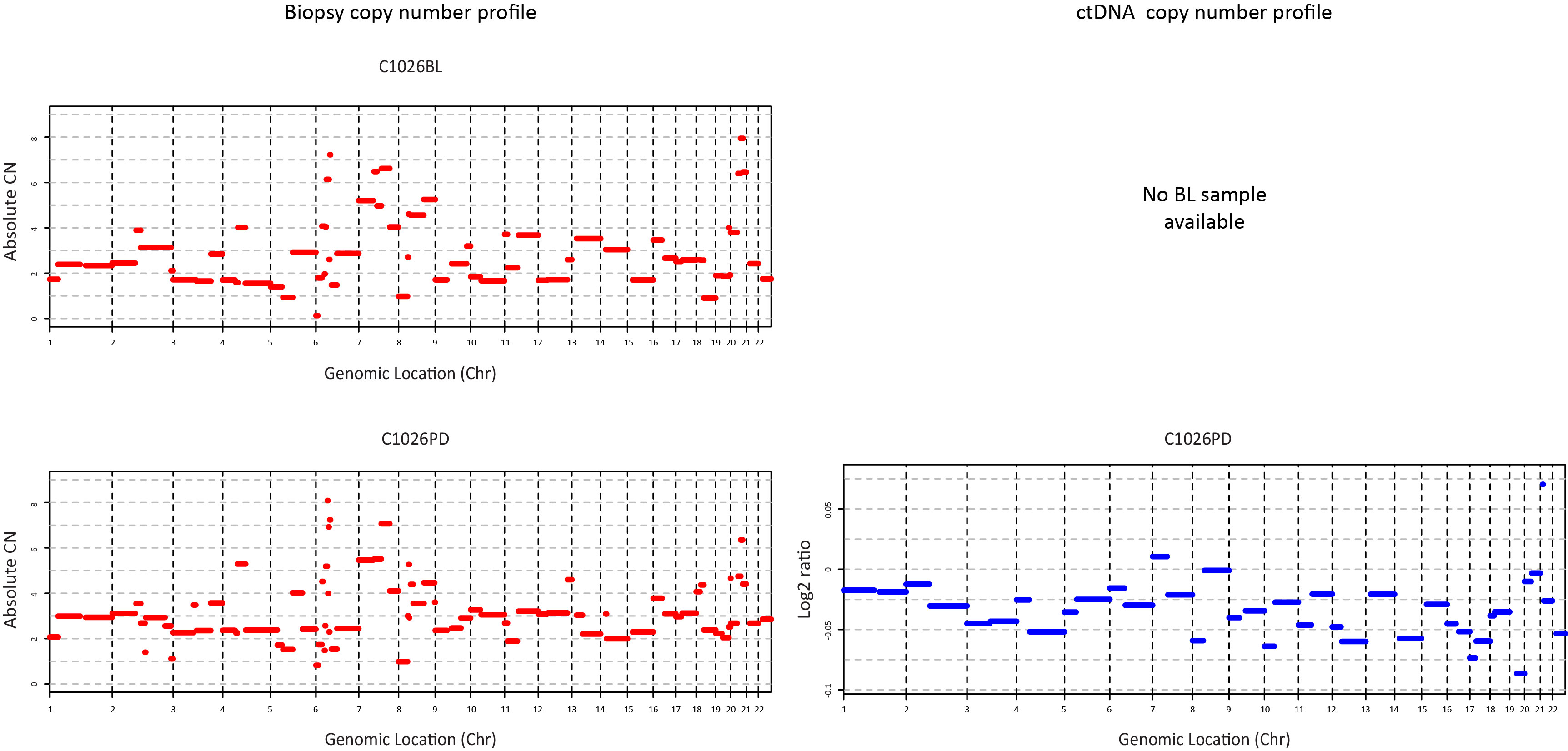


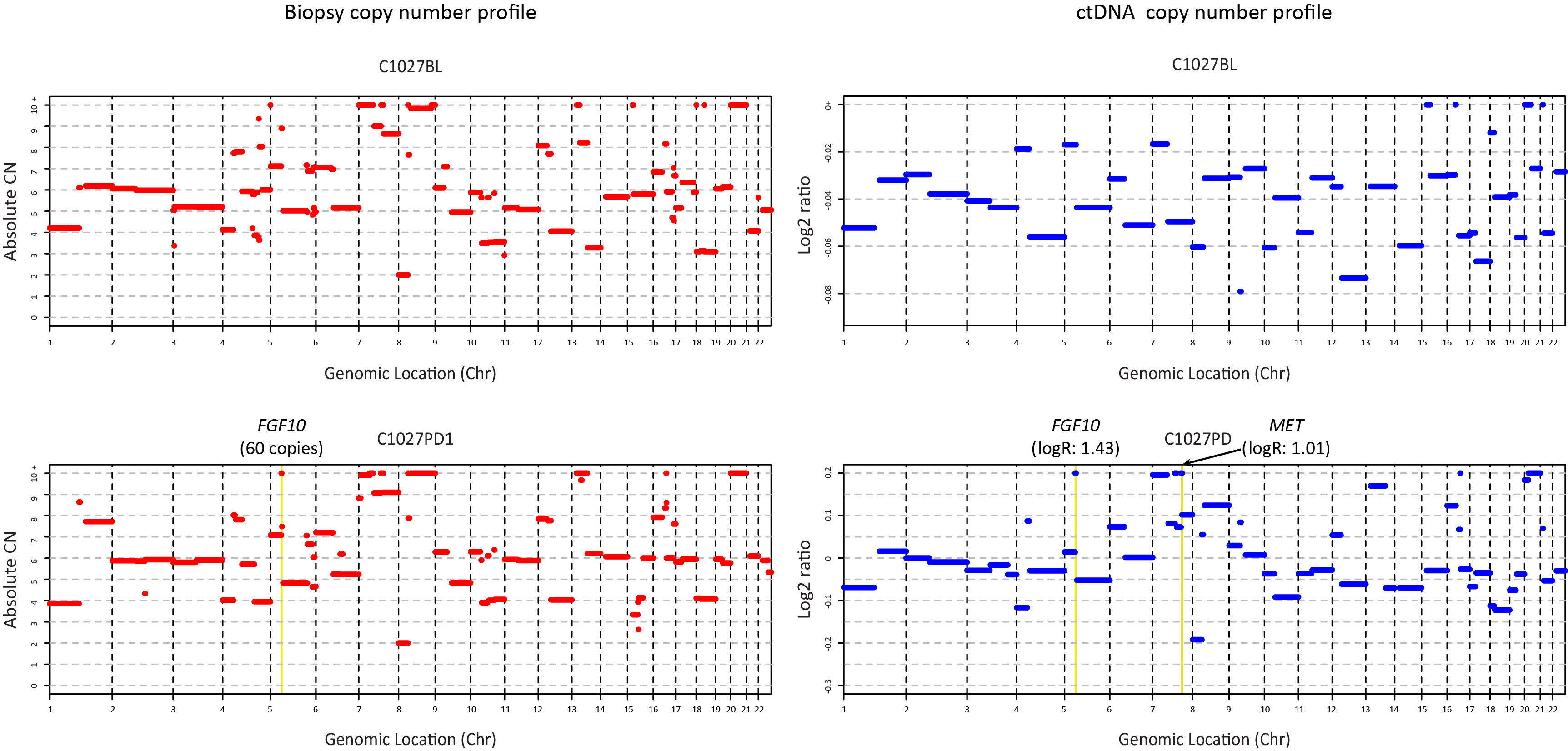


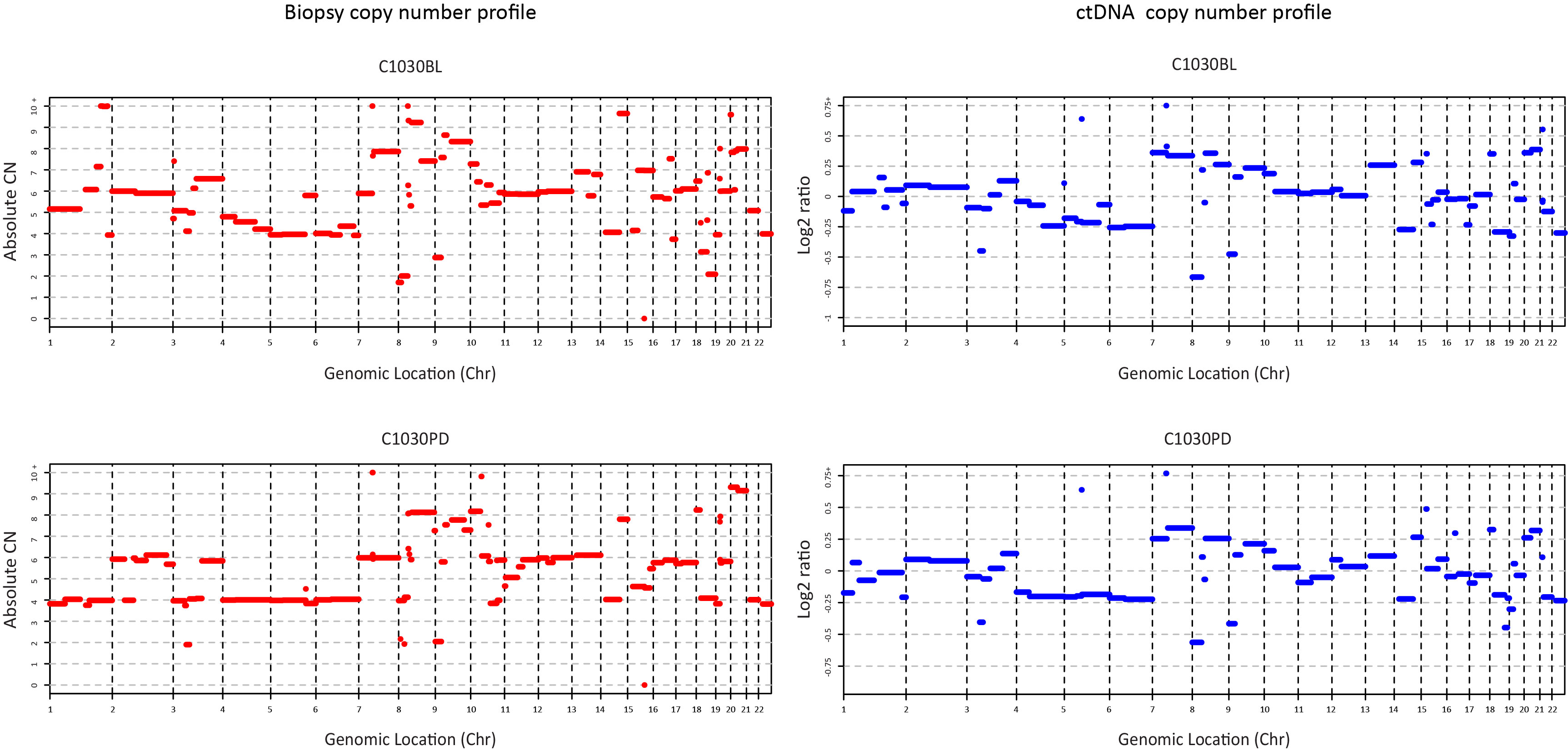


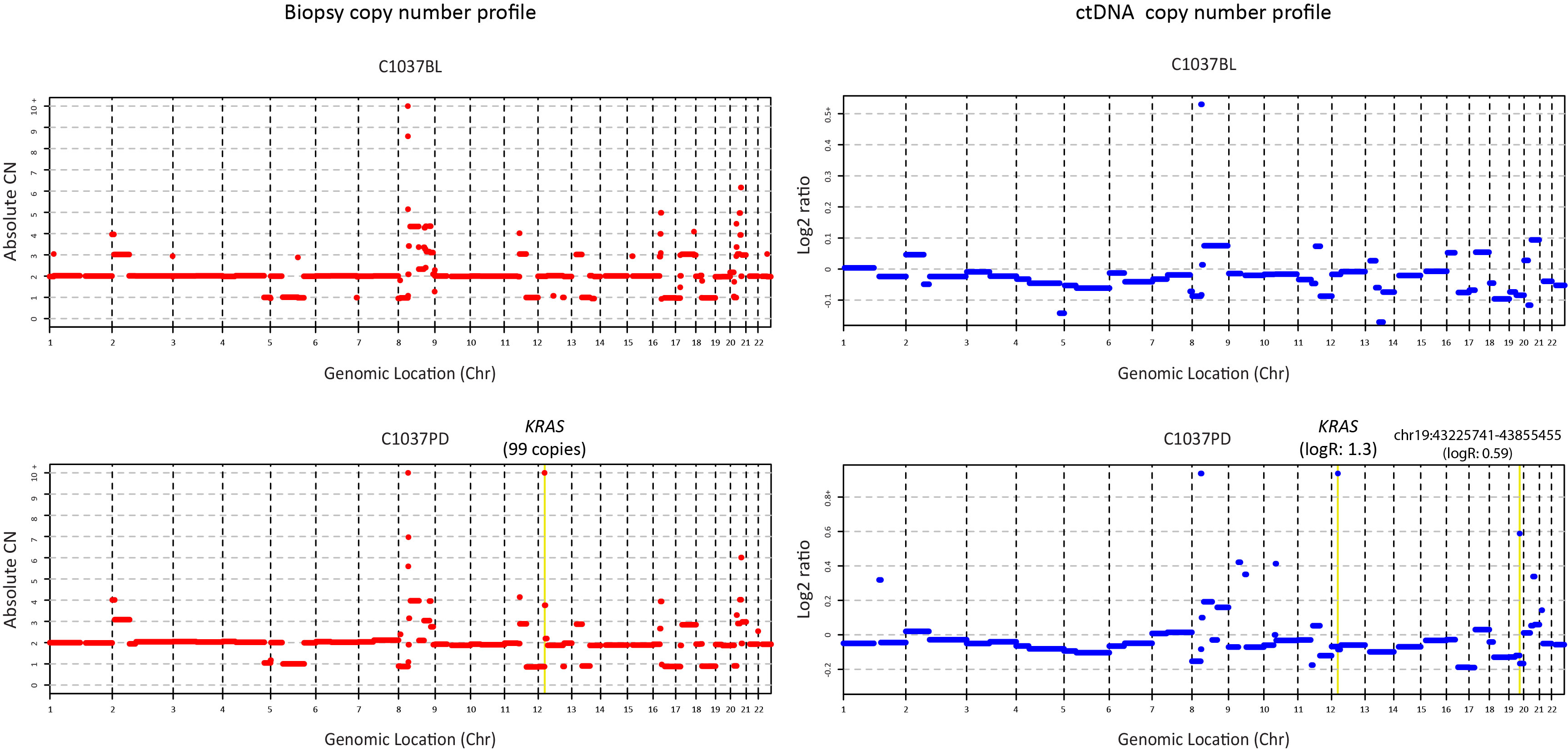


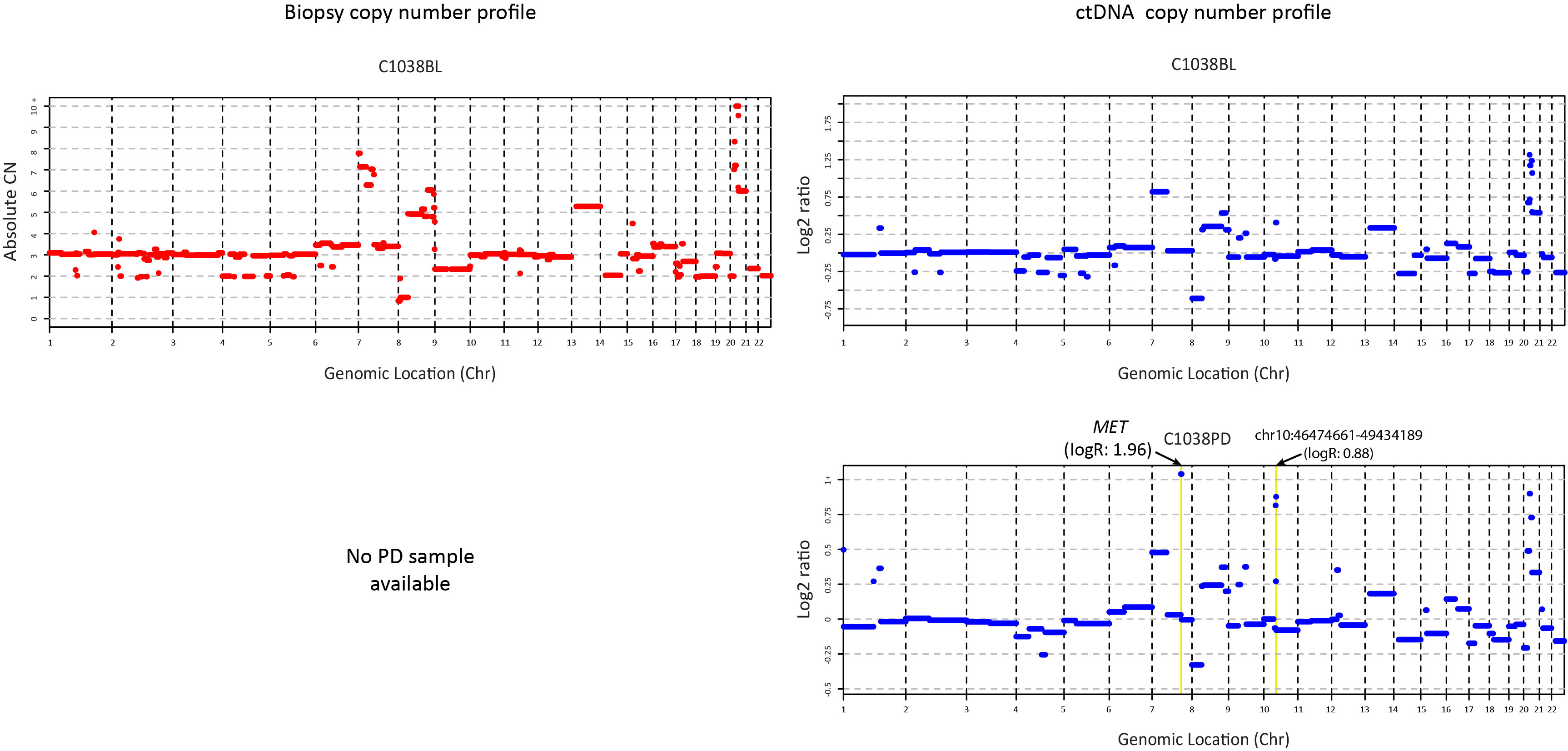


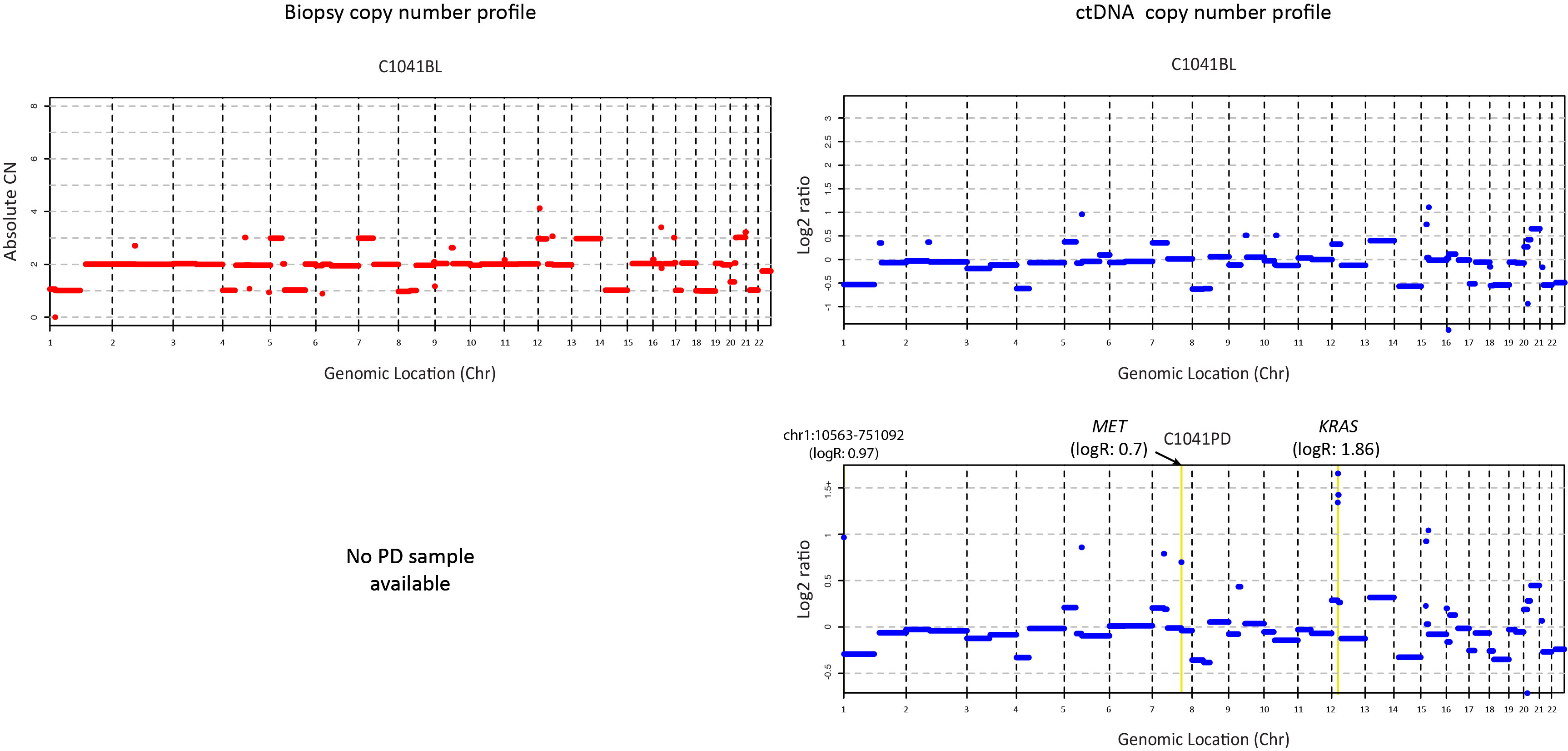


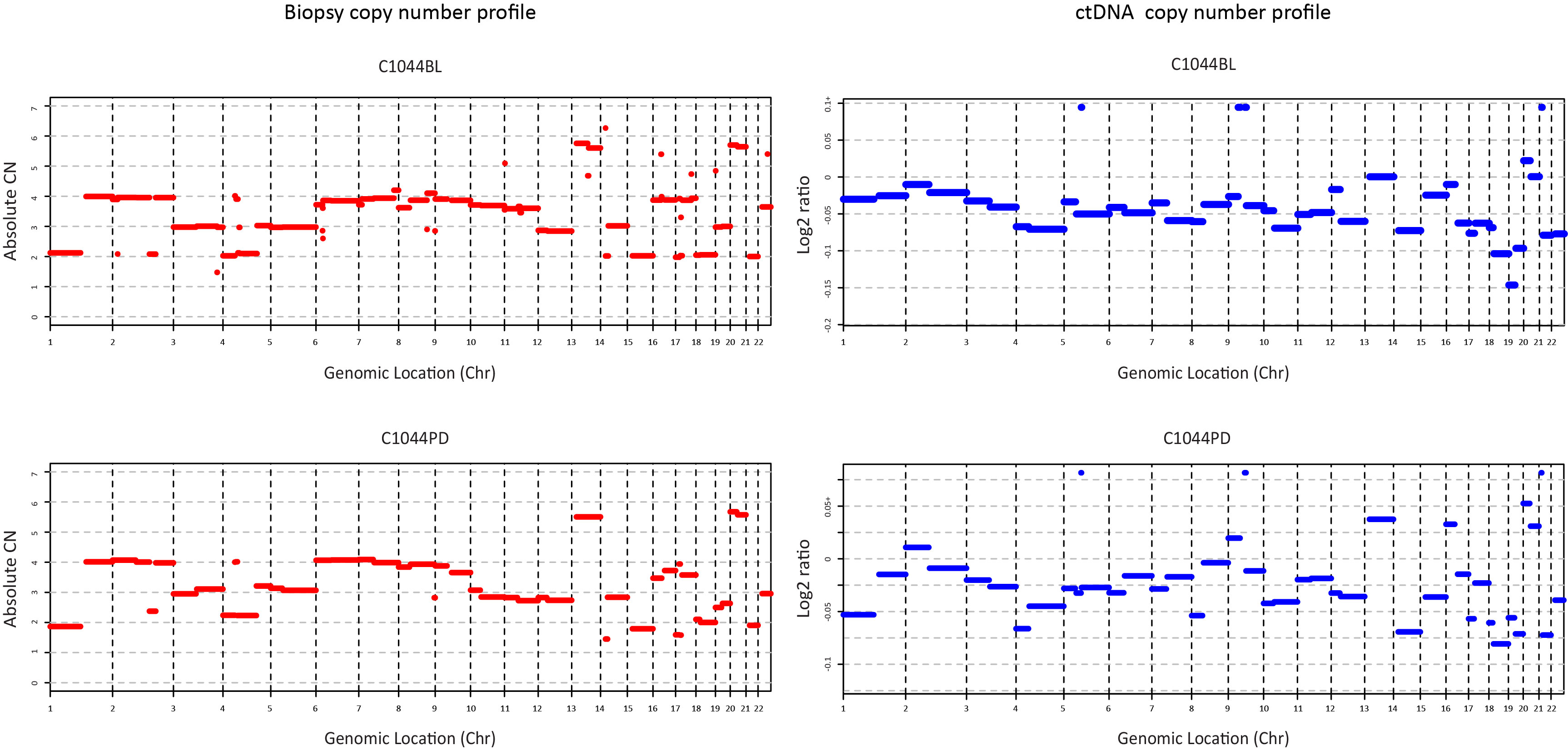

Supplement: Data S3. Copy-Number Profile Comparison of Biopsy and ctDNA Samples, Related to Figure 5 [file mmc9.zip › Data Set S3.docx]
